# Pharmacovigilance-related events, disease burden and overall efficiency of care in european countries, 1990-2021

**DOI:** 10.3389/fphar.2025.1592957

**Published:** 2025-06-27

**Authors:** Lin Yin, Shuzhi Lin, Qian Liu, Xiaoying Zhu, Wei Liu, Yifang Shen, Zimeng Li, Bianling Feng

**Affiliations:** ^1^ The Department of Pharmacy Administration, School of Pharmacy, Xi’an Jiaotong University, Xi’an, Shaanxi, China; ^2^ The Center for Drug Safety and Policy Research, Xi’ an Jiaotong University, Xi’an, Shaanxi, China

**Keywords:** global burden of disease study, pharmacovigilance-related events, quality of care index, data envelopment analysis, european countries

## Abstract

**Objectives:**

This study aimed to evaluate and compare the disease burden of pharmacovigilance-related events in European countries, to identify the factors related to pharmacovigilance-related inputs in various countries, and to analyze and compare the comprehensive benefits of pharmacovigilance-related events in various countries.

**Methods:**

Using the Global Burden of Disease Study 2021 database, we combined information on adverse effects of medical treatment and drug use disorders to identify all pharmacovigilance-related events. We used principal component analysis to synthesize six first-level indicators to compare the burden of disease in each country in each year. We used data envelopment analysis to compare the efficiency of pharmacovigilance in each country.

**Results:**

In 2021, the Quality of care index for pharmacovigilance-related events was highest in Northern and Western European countries. Looking at data from 1990 to 2021, the change node of this index in most countries occurred around 2000 to 2010, and the value changed steadily. Countries with relatively low investment in health resources and less experience in the supervision of adverse drug reactions have higher comprehensive benefits of nursing for pharmacovigilance-related events.

**Conclusion:**

The effect of the development of a system for pharmacovigilance has a hysteresis. The disease burden is affected by various factors such as population aging, human resource investment, and medical and health needs, and the policy is highly dominant.

## 1 Introduction

Pharmacovigilance was developed from drug safety warnings. In the 1960s, it mainly focused on raising public awareness of the risks of drug use. In 1961, the widespread drug-induced injuries caused by thalidomide focused global attention on drug safety. This led to the construction of systems for pre-marketing approval and post-marketing supervision of drugs in various countries. The scope of pharmacovigilance began to expand to involve the identification and monitoring of adverse drug reactions, focusing on drug side effects and drug dependence. In 2002, the World Health Organization (WHO) defined pharmacovigilance as: “Scientific research and activities to detect, evaluate, recognize and prevent adverse drug reactions or any other problems that may be related to drugs”. (Garashi et al., 2022). This definition therefore encompasses adverse drug reactions (ADRs), drug abuse, drug safety, and other aspects of drug use. Since the beginning of the 21st century, the concept of pharmacovigilance has expanded to focus on the monitoring of innovative drugs. In 2013, the United States established the National Drug Early Warning System to track new drugs and drug trends and improve the management of international high-alert drugs. (Artigiani and Wish, 2020). The scope of pharmacovigilance now covers the whole life cycle of drugs and continues to expand.

The increasing economic burden of pharmacovigilance-related diseases has become a public health problem that needs to be addressed urgently. Adverse drug events (ADEs) directly lead to increased medical costs and even disability and death. They are also a global problem. In the United States, preventable ADEs related to injectable drugs in inpatients result in healthcare costs of $2.7 billion to $5.1 billion per year, with an average of $600,000 per hospital. (Lahue et al., 2012). In Germany, the cost of drug-induced illness exceeds $136 billion per year (Mcdonnell, 2004) and a 2016 Canadian study reported that the mortality rate was four times higher for patients with ADEs than without. (Fu et al., 2019). 4.7% of hospital admissions in Spain is caused by preventable ADEs, with each event costing 3,000 euros averagely. (Airaksinen et al., 2007). The economic losses caused by ADRs worldwide are approximately $42 billion per year. (Yang et al., 2023). There are 237 million medication errors (MEs) in the UK each year, with avoidable ADEs costing the UK healthcare service £98,462,582 each year and resulting in 1,708 deaths. (Elliott et al., 2021). Medication errors and diagnostic errors are the two most common injuries in Australia, (Fu et al., 2019), and there is a 20.4% incidence of MEs in geriatric units in Indonesia. (Fu et al., 2019). In 2023, the economic loss due to perioperative MEs in the US amounted to $5.33 billion. (Langlieb et al., 2023). The total number of disability-adjusted life years (DALYs) directly caused by drug use disorder (DUD) reached 20 million in 2010, accounting for 0.8% of the total global burden of disease. (Wang et al., 2015). This figure also continues to grow, with opioid abuse being the main contributing factor. (Wang et al., 2015).

European countries are at the forefront of pharmacovigilance development, with most establishing robust regulatory systems. While economically advanced nations like Germany, France, and Sweden excel in this area, developing economies such as Bulgaria and Romania face disparities in resource allocation and implementation effectiveness. This contrast reflects the global variability in pharmacovigilance progress, making European countries typical of the development of pharmacovigilance globally.

Recent studies on pharmacovigilance often concentrate on specific diseases, with few comprehensive analyses available. In this paper, we used the database from the Global Burden of Disease Study 2021 (GBD, 2021) to create a new indicator of disease burden related to pharmacovigilance-related events. This brings together data on disease burden of the adverse effects of medical treatment (AEMT) and drug use disorders that meet the definition of pharmacovigilance. We also assessed the efficiency of pharmacovigilance systems across various countries, offering insights for nations at different developmental stages. This approach aims to guide countries in effectively utilizing limited resources to enhance their pharmacovigilance systems and reduce the burden of pharmacovigilance-related diseases.

## 2 Materials and methods

### 2.1 Data sources

The database from the Global Burden of Disease Study 2021 is held by the Institute for Health Metrics and Evaluation (IHME) at the University of Washington. It includes the burden of disease for 371 common diseases or injuries in 204 countries or regions around the world, with data taken from sources such as national and regional censuses, and vital registration systems. Diseases are coded using the International Classification of Diseases (ICD) system. Data cleansing and dissemination has been approved by the University of Washington Institutional Review Board, (GBD, 2021), and the data are internationally recognized.

### 2.2 Methods

This study screened data on two pharmacovigilance-related conditions: adverse effects of medical treatment and drug use disorders. Pharmacovigilance fully encompasses both conditions (Drug use disorders, 2020; Adverse effects of medical treatment, 2007) and we drew on a previous study combining sub-causes to analyze the burden of disease. (Golabi et al., 2021). We calculated 95% confidence intervals based on standard errors, and after obtaining the width of 95% of the uncertainty interval divided by 1.96 × 2, (Golabi et al., 2021), we combined the two causes to determine the burden of disease of pharmacovigilance-related events. The ICD-10 and ICD-9 codes corresponding to these conditions are detailed in Supplementary Appendix S1.

We selected the age-standardized values per 100,000 population for a total of 44 countries in Central Europe, Eastern Europe and Western Europe for whom data were available in the Global Burden of Disease database from 1990 to 2021. We used principal component analysis (PCA) to synthesize six measures to provide a Quality of Care Index (QCI), a comprehensive indicator of the burden of disease associated with pharmacovigilance-related events.

PCA is a data dimensionality reduction method. It allows researchers to maximize the information in the original data while highlighting the characteristics of the data. We calculated four secondary indicators (the Mortality to Incidence Ratio, the ratio of DALYs to prevalence, the ratio of prevalence to incidence, and the ratio of Years of life lost to Years lived with disability) and then performed PCA. The resulting score was the QCI value. We adjusted the QCI to give a value of between 0 and 100, and a higher value represents better quality of care. The formula is as follows, where x represents different countries. (Iezadi et al., 2023).The data processing steps for principal component analysis are shown in Supplementary Appendix S5.

$$MIR\left(x\right)=\frac{\text{Death}\left(x\right)}{Incidence\left(x\right)}$$

$$DALYs\;to\;Prevalence\left(x\right)=\frac{\text{DALYs}\left(x\right)}{Prevalence\left(x\right)}$$

$$Prevalence\;to\;Incidence\left(x\right)=\frac{Prevalence\left(x\right)}{Incidence\left(x\right)}$$

$$YLL\;to\;YLD\left(x\right)=\frac{YLL\left(x\right)}{YLD\left(x\right)}$$

$$QCI\left(x\right)=\frac{\left[PCAscore\left(x\right)-\text{minPCAscore}\right]}{\left[maxPCAscore-minPCAscore\right]}$$

Data envelopment analysis (DEA) is a quantitative analysis method based on linear planning. It is widely used in performance evaluation, and to find efficiency in resource allocation, by considering and analyzing the input and output indicators of the decision-making unit (DMU). We used the Banker, Charnes and Cooper model of DEA, which is input-oriented and considers the variability of scale returns. It is more flexible than many alternatives. (Liu et al., 2017).

max θ , subject to:

$$\sum_{j=1}^{n}\lambda_{j}x_{ij}\leq \theta x_{io},\forall i$$

$$\sum_{j=1}^{n}\lambda_{j}y_{rj}\geq y_{ro},\forall r$$

$$\sum_{j=1}^{n}\lambda_{j}=1$$

$$\lambda_{j}\geq 0,\forall j$$

where $x_{ij}$ and $y_{rj}$ represent the *i*th input and the *r*th output of the *j*th DMU, $\lambda_{j}$ are weight variables that represent the contributions of different DMUs; θ is the efficiency score of the DMU, which represents the proportion of input that needs to be reduced; and constraints $\sum_{j=1}^{n}\lambda_{j}=1$ represent variable scale compensation.

Drawing on the core pharmacovigilance indicators of WHO (Zeng, 2016) and practical experience of pharmacovigilance work, we collected relevant indicators of the pharmacovigilance system for 44 countries. These included the time since joining the WHO pharmacovigilance monitoring center, Uppsala Monitoring Centre (UMC) (UMCTIME); *per capita* domestic general government health expenditure (GOV), *per capita* health expenditure (MED), tertiary education gross enrolment ratio (EDU), number of doctors per 10,000 people (DOC), number of pharmacists per 10,000 people (PHAR) and number of nurses per 10,000 people (NUR), which are shown in Table 1. Variance inflation factors (VIFs) were used to analyze the multicollinearity of the indicators (Song et al., 2024), while the indicators were preprocessed using multiple covariance analysis. Six indicators were finally retained and each treatment is shown in Supplementary Appendix S2.

**TABLE 1:** Input-output evaluation indicators.

Indicator dimensions	Evaluation indicators
Input indicators	Time of joining the UMC
	Per capita domestic general government health expenditure (current dollars)
	Gross enrolment ratio in tertiary education
	Doctors per 10,000 population (persons)
	Pharmacists per 10,000 population (persons)
	Nurses per 10,000 population (persons)
Output indicator	QCI

Incorporated data are for 2021. UMC, Uppsala Monitoring Centre; QCI, quality of care index.

The data from this study can be obtained from the Global Health Data Exchange (GHDx), (Global Burden of Disease Study, 2021), World Bank Open Data (World Bank Open Data, 2020) and the Global Health Observatory (WHO GHO) (Global Health Observatory, 2022) open access. Data analysis used the open-source software R 4.3.3.

## 3 Results

### 3.1 Disease burden of pharmacovigilance-related events

**FIGURE 1:**
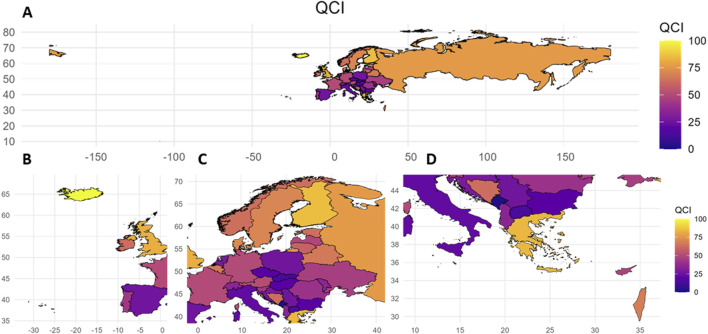
Quality of care index values for European countries, 2021. QCI, Quality of care index. A: Overall situation in European countries; B: Situation in Western European countries; C: Situation in Central European countries; D: Situation in Southern European countries.

The values and QCIs of pharmacovigilance-related events from 1990 to 2021 are shown in Supplementary Appendix S3. There were some interesting findings. For example, across 44 European countries in 2021, the QCI values were higher in Northern and Western European countries, with the top five being Iceland (68.62), Greece (60.73), Finland (58.81), the Russian Federation (53.34) and the United Kingdom (51.38). Specifically, among these countries, Greece belongs to the Southern and Eastern European countries. These countries therefore have better quality of care and relatively light disease burden from pharmacovigilance-related events. Andorra (0), San Marino (3.08), Montenegro (5.20), Slovakia (11.30) and Italy (11.99) all had lower QCI values and poorer pharmacovigilance-related quality of care (Supplementary Appendix S3; Figure 1).

From 1990 to 2021, the QCI of various countries changed to varying degrees. For example, Andorra’s QCI decreased from 3.41 in 1990 to 0.00 in 2021, when it was the lowest QCI value. Countries with a similar significant decline were San Marino (−74.39%), Italy (−55.04%), Bosnia and Herzegovina (−46.50%), Switzerland (−43.59%), Cyprus (−42.50%), Spain (−40.63%), and France (−39.29%). Bosnia and Herzegovina decreased from its highest value of 89.52 in 1990 to 47.89 in 2021, showing a significant downward trend.

**FIGURE 2:**
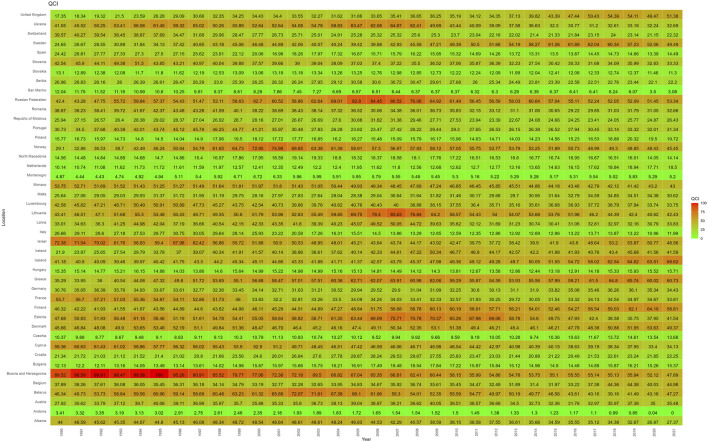
Heat map distribution of QCI values for 44 European countries (1990–2021). Higher QCI values represent better pharmacovigilance care. QCI, Quality of care index.

Of the countries that showed sustained growth in their QCI from 1990 to 2021, the UK increased its QCI from 17.35 to 51.38, with a steady growth rate of 196.21%. Sweden fluctuated from 24.65 to 49.49, with a growth rate of 100.76%, especially in the early 2000s. Ireland fluctuated from 21.89 to 41.59, with a growth rate of 89.94%, and significant growth in the early 2000s. Other countries with a significant growth trend were the Netherlands (82.46%), Greece (72.12%), Iceland (66.62%), Norway (56.17%) and Finland (45.86%). Generally speaking, the change node for the QCI in most countries occurred around 2000 to 2010, and the value changed steadily, with only a few countries showing fluctuating changes over time (Figure 2).

### 3.2 Data envelopment analysis results

Considering the availability of data, we included cross-sectional data from 36 countries in 2021 for data envelopment analysis, with QCI as the output indicator and DOC, PHAR, NUR, GOV, EDU and UMCTIME as input indicators. Investments in pharmacovigilance resources vary between countries, and the descriptive analyses are shown in Supplementary Appendix S2.

**FIGURE 3:**
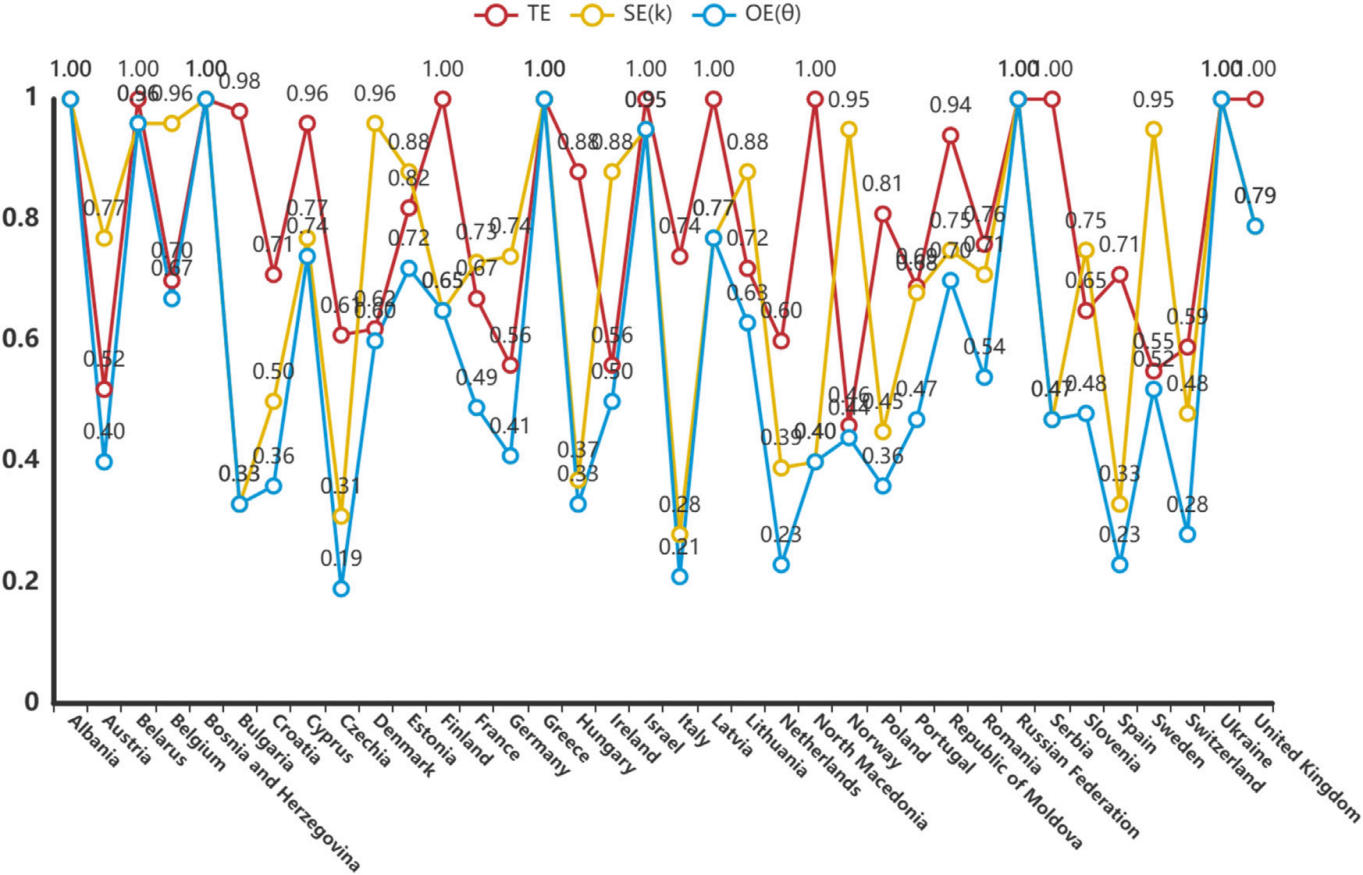
Efficiency of pharmacovigilance-related events care in 36 European countries, 2021. TE, technical efficiency; SE: scale efficiency; OE: overall efficiency.

The DEA model was used to analyze the technical efficiency (TE), scale efficiency (SE) and overall efficiency (OE) of 36 countries. Countries with strong efficient DEA included Albania, Bosnia and Herzegovina, Greece, the Russian Federation and Ukraine. These countries obtained a value of one for TE, SE and OE, indicating that they are in the optimal state in terms of rational use and scale of pharmacovigilance resources. Countries with non-DEA efficiency showed varying degrees of inefficiency. For example, Austria had a TE was 0.517, SE of 0.774, and OE of 0.4, indicating that the country has significant room for improvement in the technology and scale of resource utilization in its pharmacovigilance system. The OE of countries such as Germany and France were also lower than 1, reflecting the underutilization of factors and the increasing returns to scale that could be achieved. Overall, the Czech Republic, Italy, the Netherlands, Spain, Switzerland, Bulgaria, Hungary, Croatia and Poland have lower OE. The analysis of the relaxation variable S− further revealed the redundancy of resources in the pharmacovigilance system. For example, the S− in the UK is 2830.323, indicating that more efficiency could be achieved by reducing inputs: that is, there is a waste of pharmacovigilance resources. (Table 2; Figure 3).

**TABLE 2:** Analysis of the effectiveness of quality of care in 36 countries.

Country	TE	SE(k)	OE(θ)	S-	S+	Efficiency
Albania	1	1	1	0	0	Strong DEA efficiency
Austria	0.517	0.774	0.4	1270.765	0	non-DEA efficiency
Belarus	1	0.959	0.959	35.799	0	non-DEA efficiency
Belgium	0.697	0.965	0.672	2416.574	0	non-DEA efficiency
Bosnia and Herzegovina	1	1	1	0	0	Strong DEA efficiency
Bulgaria	0.985	0.332	0.327	8.064	0	non-DEA efficiency
Croatia	0.706	0.504	0.356	256.451	0	non-DEA efficiency
Cyprus	0.961	0.769	0.739	1763.715	0	non-DEA efficiency
Czechia	0.612	0.309	0.189	327.138	0	non-DEA efficiency
Denmark	0.621	0.962	0.597	2363.572	0	non-DEA efficiency
Estonia	0.822	0.883	0.725	770.874	0	non-DEA efficiency
Finland	1	0.65	0.65	1859.332	0	non-DEA efficiency
France	0.672	0.729	0.49	1537.212	0	non-DEA efficiency
Germany	0.562	0.736	0.414	1667.556	0	non-DEA efficiency
Greece	1	1	1	0	0	Strong DEA efficiency
Hungary	0.884	0.373	0.33	168.178	0	non-DEA efficiency
Ireland	0.565	0.878	0.496	1777.146	0	non-DEA efficiency
Israel	1	0.948	0.948	815.365	0	non-DEA efficiency
Italy	0.736	0.284	0.209	324.58	0	non-DEA efficiency
Latvia	1	0.769	0.769	817.881	0	non-DEA efficiency
Lithuania	0.716	0.88	0.631	244.995	0	non-DEA efficiency
Netherlands	0.604	0.389	0.235	727.705	0	non-DEA efficiency
North Macedonia	1	0.4	0.4	5.615	0	non-DEA efficiency
Norway	0.462	0.954	0.44	2059.047	0	non-DEA efficiency
Poland	0.807	0.451	0.364	152.014	0	non-DEA efficiency
Portugal	0.693	0.678	0.47	173.49	0	non-DEA efficiency
Republic of Moldova	0.939	0.749	0.704	6.294	0	non-DEA efficiency
Romania	0.764	0.706	0.539	168.908	0	non-DEA efficiency
Russian Federation	1	1	1	0	0	Strong DEA efficiency
Serbia	1	0.465	0.465	25.304	0	non-DEA efficiency
Slovenia	0.649	0.747	0.485	692.928	0	non-DEA efficiency
Spain	0.707	0.333	0.235	353.078	0	non-DEA efficiency
Sweden	0.553	0.947	0.524	1928.168	0	non-DEA efficiency
Switzerland	0.587	0.481	0.282	408.046	0	non-DEA efficiency
Ukraine	1	1	1	0	0	Strong DEA efficiency
United Kingdom	1	0.785	0.785	2830.323	0	non-DEA efficiency

The redundancy of countries on different elements is shown in Supplementary Appendix S4 and could steer countries towards adjusting their resource allocation. The output deficiencies analysis showed that the output deficiencies rate was close to zero in all the countries studied, indicating that their output efficiency was high.

The meaning of the relaxation variable S- is ‘to achieve the target efficiency when the input is reduced’, and the meaning of the relaxation variable S+ is to be ‘to achieve the target efficiency when the output is increased’. If the OE = 1 and S- and S+ are both 0, then ‘Strong DEA efficiency’. TE, technical efficiency; SE: scale efficiency; OE: overall efficiency.

There were significant national differences in the return to scale. Albania, Bosnia and Herzegovina, Greece, the Russian Federation, and Ukraine all had a return to scale coefficient of 1, indicating that the scale gains in pharmacovigilance care quality in these countries are constant and have reached the optimal state of efficiency. Denmark (1.013), Sweden (1.033), the United Kingdom (1.073), Finland (1.228) and Belarus (1.234) all showed diminishing returns to scale, indicating that the current pharmacovigilance system is too large, and downsizing could improve efficiency. In contrast, countries such as Norway (0.949), Germany (0.719), France (0.706), Portugal (0.588) and Switzerland (0.466) showed increasing returns to scale, meaning they could increase their returns by scaling up their pharmacovigilance system. (Table 3).

**TABLE 3:** Return to scale in European countries.

Country	Coefficient of return to scale	Type of returns to scale
Albania	1	Constant returns to scale
Bosnia and Herzegovina	1	
Greece	1	
Russian Federation	1	
Ukraine	1	
Austria	0.701	Increasing returns to scale
Belgium	0.939	
Bulgaria	0.276	
Croatia	0.448	
Cyprus	0.637	
Czechia	0.278	
Estonia	0.83	
France	0.706	
Germany	0.719	
Hungary	0.299	
Ireland	0.868	
Israel	0.915	
Italy	0.226	
Latvia	0.632	
Lithuania	0.835	
Netherlands	0.384	
North Macedonia	0.305	
Norway	0.949	
Poland	0.39	
Portugal	0.588	
Republic of Moldova	0.665	
Romania	0.681	
Serbia	0.442	
Slovenia	0.696	
Spain	0.275	
Switzerland	0.466	
Belarus	1.234	Diminishing returns to scale
Denmark	1.013	
Finland	1.228	
Sweden	1.033	
United Kingdom	1.073	

## 4 Discussion

Most of the current studies using the Global Burden of Disease databases select first-level research indicators, such as incidence. The results of different disease burden studies are therefore neither universal nor comparable. In this study, QCI was a combination of six first-level indicators, and the two types of events or conditions, AEMT and DUD, were combined into a single set of pharmacovigilance-related events. This meant that our results were more comprehensive and representative.

We found that in 2021, the quality of pharmacovigilance care was better in Northern and Western Europe than in other areas. Drug abuse is an important component of pharmacovigilance-related disorders. A 2018 study found that more than a quarter of adults aged 15–64 in the EU had used illegal drugs. (Organization for Economic Co-operation and Development, 2018). The availability and misuse of new psychoactive substances remains a major public health challenge in Europe, with rates of abuse in Eastern and South-Eastern Europe significantly higher than the global average. (United Nations, 2015). This may be related to the involvement of organised criminal groups in the manufacture and tracing of these substances in these areas. Studies have shown that underinvestment in healthcare and pharmaceuticals in Southern and Eastern European countries has led to worsening health outcomes and increased household burdens (Yfantopoulos, 2023). Greece has a nationwide network of drug abuse prevention centers to cope with the increase in the number of young and adult drug users, perhaps explaining its better performance in the QCI. (Dritsas and Theodoratou, 2017). As the EU integration process progresses, the traditional health regulatory system is also gradually being replaced by a market-based system, which may lead to more complex and unpredictable drug use patterns in some countries. (Jensen and Berner, 1965).

Interestingly, our findings revealed that countries with relatively low investments in health resources and limited experience in ADR regulation often demonstrated more efficient pharmacovigilance systems. For instance, among the countries noted for optimal efficiency, Albania became a member of the UMC in 2020. Similarly, Bosnia and Herzegovina, which established a national pharmacovigilance center, remained largely inactive in this area until 2017. (Martin et al., 2020). The United Kingdom joined the UMC in 1968, (UMC, 2023), and its QCI increased steadily from 1990 to 2021, but its score for efficiency was not high. A similar picture was found in many other countries and for other factors. This suggests that the effect of building a pharmacovigilance system has a hysteresis, which is consistent with the conclusions of previous studies. (Lin et al., 2024). The assessment of the pharmacovigilance system needs to be carried out after a certain number of ADR reports, and therefore the short-term efficiency of pharmacovigilance is significant in countries with little data and a fledgling pharmacovigilance system, such as Albania. However, the status of pharmacovigilance in these countries needs long-term observation and follow-up studies to verify its ongoing effectiveness and sustainability. In conclusion, the disease burden of pharmacovigilance-related events is relatively heavy, and care is relatively inefficient in the more developed countries. However, the disease burden shows a tendency to improve with the development of a pharmacovigilance system.

Economically developed countries often have more resources for drug development and clinical trials, allowing more diseases to be diagnosed and treated. However, this also means that more drugs are being used, increasing the risk of drug-related adverse reactions and drug interactions. The low fertility rate in European countries has led to an increasingly aging population. (Population structure and ageing) It is known that poor medication adherence, reduced risk awareness of drug use, and multi-drug sharing among older people increase the burden of pharmacovigilance-related events. One study from Sweden reported that the incidence of potential drug–drug interactions among older people was 31%. (Krupa et al., 2021). Factors such as the increase in the development and use of new drugs, and the improvement and strict implementation of pharmacovigilance systems, are also important factors in increasing the burden of disease. The tragedies related to drug safety problems in the 20th century prompted some countries to pay much more attention to the safety of new drugs. Germany, Italy and Spain established new ADR reporting systems in the late 1960s and 1970s. (Wang, 1996). France established a voluntary reporting system in 1979. (Tian, 2007). The WHO did not recommend that countries should establish national pharmacovigilance centers until 2002, when pharmacovigilance began to be promoted and standardized worldwide. (Qin et al., 2013). The European Medicines Agency launched new pharmacovigilance regulations in 2012, set up the pharmacovigilance database EudraVigilance, and in 2013 developed new Pharmacovigilance Practice Guidelines. (European Medicines Agency website, 2012). The timing of these developments in pharmacovigilance is a strong explanation for the concentration of QCI changes around 2000 to 2010 in various countries. In other words, the policy dominance of the disease burden of pharmacovigilance is strong.

From the perspective of resources for pharmacovigilance, the DEA results show that, apart from the most efficient countries, there is redundancy in the input of doctors, nurses and pharmacists in most countries, i.e., there may be insufficient working hours, improper staffing or increased costs of team coordination and communication. Improvements are therefore possible to coordination and organization management. However, lack of knowledge and training is one of the reasons for many ADEs, and education for junior physicians is considered key to improving prescription safety. (Maxwell et al., 2007). The UK’s report on reducing serious adverse drug reactions through the Yellow Card Scheme highlights the importance of nurses in drug safety monitoring. (Griffith, 2013). Patients and the public also play a role in pharmacovigilance, although this role is not well understood. This leads to a lack of proper supervision of the safe use of drugs, affecting the development of pharmacovigilance. (Gildeeva and Belostotsky, 2017). The European Medicines Agency has developed prescription guidelines and patient alert cards for specific drugs, and patient medication safety awareness assessments were conducted in France, Germany, Spain, and the United Kingdom between 2014 and 2015. (Zografo et al., 2022). Some countries with insufficient pharmacovigilance could increase the enrollment rate in higher education and strengthen public awareness education to improve pharmacovigilance.

This research had some limitations. First, the results will be affected by the data quality of the Global Burden of Disease 2021. It is a high quality study, but its internal estimation methods and assumptions still have some limitations when applied to the complex real world. Second, the definition and classification of pharmacovigilance-related diseases draws on the Global Burden of Disease database, which may lead to difficulties in comparing our findings with the results of other studies. Third, the pharmacovigilance nursing input factors included in this paper may not fully cover all the resources used in pharmacovigilance.

## 5 Conclusion

The effect of developing pharmacovigilance has a lagging effect. Many countries with better pharmacovigilance show a heavier burden of disease of pharmacovigilance-related events, while the countries that have just established pharmacovigilance laws and regulations show a low disease burden and high nursing efficiency because of the small number of reports, alongside other reasons. In general, the disease burden of pharmacovigilance-related events is affected by a variety of factors such as age of the population, medical and health needs, human resource investment, and the development of a regulatory system, including in particular laws and regulations.

## Data Availability

The original contributions presented in the study are included in the article/Supplementary Material, further inquiries can be directed to the corresponding author.
