# Supplementary material for: Pharmacovigilance-related events, disease burden and overall efficiency of care in european countries, 1990-2021
Source: Front Pharmacol. 2025 Jun 27;16:1592957. doi: 10.3389/fphar.2025.1592957 (PMC12245805; doi:10.3389/fphar.2025.1592957)
Supplement: Supplementary file 1 [file Supplementaryfile1.docx]

**Appendix 1.** International Classification of Diseases (ICD) codes mapped to non-fatal causes and injuries in the GBD 2021

| **Cause Name** | **ICD10** | **ICD9** |
| --- | --- | --- |
| Adverse effects of medical treatment | D69.5-D69.59, D70.1-D70.2, D78-D78.89, D89.81-D89.813, E03.2, E06.4, E09-E09.9, E16.0, E23.1, E24.2, E27.3, E36-E36.8, E66.1, E87.0-E87.99, E89-E89.9, G21.0-G21.19, G24.0-G24.09, G25.1, G25.4, G25.6-G25.79, G62.0, G72.0, G93.7, G96.0, G96.11, G97-G97.9, H05.33-H05.339, H05.42-H05.53, H59-H59.89, H91.0-H91.09, H95-H95.9, I95.2-I95.81, I97-I97.9, J70-J70.4, J95-J95.9, K08.5-K08.59, K43-K43.9, K52.0, K62.7, K68.11, K91-K91.9, K94-K95.89, L23.3, L27.0-L27.1, L56.0-L56.1, L64.0, L76-L76.82, M10.2-M10.29, M87.1-M87.19, M96-M96.9, N14-N14.4, N30.4-N30.41, N46.021, N46.121, N52.2-N52.39, N65-N65.1, N99-N99.9, P93-P93.8, P96.2, P96.5, R50.2-R50.83, Y40-Y84.9, Y88-Y88.3, Z21.0, Z42-Z51.9, Z88-Z94.0, Z94.6-Z99.9 | 244.0-244.1, 244.3, 251.3, 253.7, 279.5-279.53, 331.81, 333.92, 349-349.9, 357.6, 359.24, 379.6-379.63, 440.3-440.32, 457.0, 458.2-458.29, 518.6-518.7, 519.0-519.1, 525.6-525.79, 526.62-526.63, 530.86-530.87, 536.4-536.49, 539-539.9, 551.2-551.29, 552.2-552.29, 553.2-553.29, 564.2-564.4, 569.6-569.8, 579.3, 595.82, 596.81-596.83, 598.2, 612-612.1, 779.4-779.5, 780.62-780.66, 995.89, E870-E876.9, E878-E879.9, E930-E949.9, V44-V45, V45.2-V45.4, V45.7, V45.77, V45.79-V45.8, V45.87-V45.89 |
| Drug use disorders | F11-F19.99, P96.1, R78.1-R78.9 | 292-292.9, 304-304.93, 305.1-305.93, E850.0-E850.29, V15.8-V15.83, V15.85-V15.86 |
