# Supplementary material for: Pharmacovigilance-related events, disease burden and overall efficiency of care in european countries, 1990-2021
Source: Front Pharmacol. 2025 Jun 27;16:1592957. doi: 10.3389/fphar.2025.1592957 (PMC12245805; doi:10.3389/fphar.2025.1592957)
Supplement: Supplementary file 2 [file Supplementaryfile5.docx]

**Appendix 5 Data processing steps prior to principal component analysis**

Obtain age-standardized primary indicators for the included countries in the GBD database and calculate the four secondary indicators.

$$MIR(x)=\frac{\mathrm{Death}(x)}{Incidence(x)}$$

$$DALYs to Prevalence(x)=\frac{\mathrm{DALYs}(x)}{Prevalence(x)}$$

$$Prevalence to Incidence(x)=\frac{Prevalence(x)}{Incidence(x)}$$

$$YLL to YLD(x)=\frac{YLL(x)}{YLD(x)}$$

Using principal component analysis, the secondary indicators will be integrated into a single index from 0-100, the Quality of Care Index (QCI). The principal component analysis method yields the formula for calculating the QCI for this thesis as:

$$\mathrm{PC}A_{\mathrm{score}}\left( x \right)= 0.585\zeta MIR\left( x \right)+ 0.597\zeta YLL to YLD \left( x \right)-. 0.012\zeta DALY to Prevalence\left( x \right)+ 0.549\zeta Prevalence to Incidence(x)$$

Before calculating the QCI, all secondary indicators are be first transformed to standard (ζ) -1 to 1 spectrum by this formula：

$$\zeta MIR (x)= \frac{MIR\left( x \right) - \mu}{\sigma}$$

Where x is the data point (e.g. age-standardized MIR of Albania in 1990 for both sexes), μ is the mean, and σ is the standard deviation of the variable in data.

QCI was retrieved by re-scaling the PCAscore into 0-100 spectrum.

$$QCI \left( x \right)= \frac{[PCA_{score} (x) - min PCA_{score} ]}{[max PCA_{score} - min PCA_{score} )}$$
