# Supplementary material for: Pharmacovigilance-related events, disease burden and overall efficiency of care in european countries, 1990-2021
Source: Front Pharmacol. 2025 Jun 27;16:1592957. doi: 10.3389/fphar.2025.1592957 (PMC12245805; doi:10.3389/fphar.2025.1592957)
Supplement: Supplementary file 3 [file Supplementaryfile3.docx]

**Appendix 3 Table 1.** The values and QCIs of pharmacovigilance-related events from 1990 to 2021

| **location** | **year** | **DALY** | **Death** | **Incidence** | **prevalence** | **YLD** | **YLL** | **MIR** | **DALYstoPrevalence** | **PrevalencetoIncidence** | **YLLtoYLD** | **QCI** |
| --- | --- | --- | --- | --- | --- | --- | --- | --- | --- | --- | --- | --- |
| Albania | 1990 | 151.25 | 2.03 | 377.06 | 502.46 | 60.31 | 90.93 | 0.01 | 0.30 | 1.33 | 1.51 | 44.00 |
| Albania | 1991 | 157.46 | 2.11 | 377.33 | 503.08 | 60.74 | 96.72 | 0.01 | 0.31 | 1.33 | 1.59 | 46.59 |
| Albania | 1992 | 156.54 | 2.06 | 377.93 | 504.25 | 61.13 | 95.41 | 0.01 | 0.31 | 1.33 | 1.56 | 45.62 |
| Albania | 1993 | 157.62 | 2.04 | 378.82 | 505.84 | 61.88 | 95.75 | 0.01 | 0.31 | 1.34 | 1.55 | 45.35 |
| Albania | 1994 | 157.98 | 2.01 | 379.96 | 507.74 | 62.68 | 95.30 | 0.01 | 0.31 | 1.34 | 1.52 | 44.67 |
| Albania | 1995 | 159.48 | 2.04 | 381.32 | 509.83 | 63.43 | 96.05 | 0.01 | 0.31 | 1.34 | 1.51 | 44.80 |
| Albania | 1996 | 162.09 | 2.08 | 383.18 | 511.44 | 64.75 | 97.34 | 0.01 | 0.32 | 1.33 | 1.50 | 45.13 |
| Albania | 1997 | 167.36 | 2.14 | 385.57 | 512.24 | 66.78 | 100.58 | 0.01 | 0.33 | 1.33 | 1.51 | 46.08 |
| Albania | 1998 | 175.53 | 2.24 | 388.18 | 512.91 | 68.79 | 106.74 | 0.01 | 0.34 | 1.32 | 1.55 | 48.34 |
| Albania | 1999 | 179.50 | 2.29 | 390.69 | 514.07 | 70.82 | 108.68 | 0.01 | 0.35 | 1.32 | 1.53 | 48.72 |
| Albania | 2000 | 181.24 | 2.31 | 392.70 | 516.12 | 72.21 | 109.03 | 0.01 | 0.35 | 1.31 | 1.51 | 48.54 |
| Albania | 2001 | 183.43 | 2.34 | 394.29 | 518.64 | 73.42 | 110.01 | 0.01 | 0.35 | 1.32 | 1.50 | 48.64 |
| Albania | 2002 | 184.48 | 2.37 | 395.82 | 520.98 | 74.18 | 110.30 | 0.01 | 0.35 | 1.32 | 1.49 | 48.61 |
| Albania | 2003 | 185.65 | 2.40 | 397.31 | 523.29 | 74.97 | 110.68 | 0.01 | 0.35 | 1.32 | 1.48 | 48.61 |
| Albania | 2004 | 186.30 | 2.40 | 398.83 | 525.77 | 75.81 | 110.49 | 0.01 | 0.35 | 1.32 | 1.46 | 48.24 |
| Albania | 2005 | 184.84 | 2.35 | 400.43 | 528.60 | 76.75 | 108.09 | 0.01 | 0.35 | 1.32 | 1.41 | 46.83 |
| Albania | 2006 | 182.19 | 2.26 | 402.69 | 532.51 | 78.17 | 104.02 | 0.01 | 0.34 | 1.32 | 1.33 | 44.53 |
| Albania | 2007 | 179.46 | 2.16 | 405.78 | 537.62 | 79.33 | 100.13 | 0.01 | 0.33 | 1.32 | 1.26 | 42.29 |
| Albania | 2008 | 177.74 | 2.12 | 409.14 | 543.07 | 80.73 | 97.01 | 0.01 | 0.33 | 1.33 | 1.20 | 40.57 |
| Albania | 2009 | 175.14 | 2.05 | 412.22 | 548.04 | 82.04 | 93.09 | 0.00 | 0.32 | 1.33 | 1.13 | 38.59 |
| Albania | 2010 | 175.66 | 2.05 | 414.47 | 551.72 | 83.00 | 92.66 | 0.00 | 0.32 | 1.33 | 1.12 | 38.15 |
| Albania | 2011 | 178.23 | 2.07 | 416.06 | 554.19 | 83.88 | 94.35 | 0.00 | 0.32 | 1.33 | 1.12 | 38.58 |
| Albania | 2012 | 176.55 | 2.05 | 417.54 | 556.25 | 84.53 | 92.02 | 0.00 | 0.32 | 1.33 | 1.09 | 37.55 |
| Albania | 2013 | 175.03 | 2.02 | 418.95 | 558.09 | 85.15 | 89.88 | 0.00 | 0.31 | 1.33 | 1.06 | 36.61 |
| Albania | 2014 | 173.02 | 1.99 | 420.32 | 559.84 | 85.39 | 87.63 | 0.00 | 0.31 | 1.33 | 1.03 | 35.66 |
| Albania | 2015 | 171.02 | 1.96 | 421.66 | 561.62 | 85.90 | 85.12 | 0.00 | 0.30 | 1.33 | 0.99 | 34.59 |
| Albania | 2016 | 174.62 | 2.01 | 423.23 | 564.05 | 86.57 | 88.05 | 0.00 | 0.31 | 1.33 | 1.02 | 35.55 |
| Albania | 2017 | 175.41 | 1.99 | 425.12 | 567.27 | 87.75 | 87.67 | 0.00 | 0.31 | 1.33 | 1.00 | 35.12 |
| Albania | 2018 | 175.08 | 1.96 | 426.94 | 570.34 | 88.65 | 86.43 | 0.00 | 0.31 | 1.34 | 0.98 | 34.38 |
| Albania | 2019 | 171.53 | 1.88 | 428.31 | 572.40 | 89.17 | 82.36 | 0.00 | 0.30 | 1.34 | 0.92 | 32.67 |
| Albania | 2020 | 156.39 | 1.63 | 430.53 | 574.38 | 89.06 | 67.33 | 0.00 | 0.27 | 1.33 | 0.76 | 26.87 |
| Albania | 2021 | 157.39 | 1.65 | 431.42 | 572.70 | 88.84 | 68.55 | 0.00 | 0.27 | 1.33 | 0.77 | 27.37 |
| Andorra | 1990 | 114.99 | 0.61 | 391.70 | 877.97 | 96.29 | 18.71 | 0.00 | 0.13 | 2.24 | 0.19 | 3.41 |
| Andorra | 1991 | 115.35 | 0.60 | 391.90 | 879.97 | 96.88 | 18.48 | 0.00 | 0.13 | 2.25 | 0.19 | 3.32 |
| Andorra | 1992 | 115.61 | 0.60 | 391.82 | 879.25 | 97.06 | 18.55 | 0.00 | 0.13 | 2.24 | 0.19 | 3.35 |
| Andorra | 1993 | 115.55 | 0.59 | 391.95 | 879.91 | 97.59 | 17.97 | 0.00 | 0.13 | 2.24 | 0.18 | 3.19 |
| Andorra | 1994 | 115.47 | 0.59 | 392.31 | 879.09 | 97.76 | 17.71 | 0.00 | 0.13 | 2.24 | 0.18 | 3.13 |
| Andorra | 1995 | 115.26 | 0.58 | 392.44 | 877.72 | 97.86 | 17.40 | 0.00 | 0.13 | 2.24 | 0.18 | 3.02 |
| Andorra | 1996 | 115.12 | 0.57 | 392.77 | 876.72 | 98.05 | 17.07 | 0.00 | 0.13 | 2.23 | 0.17 | 2.91 |
| Andorra | 1997 | 114.62 | 0.56 | 394.02 | 877.78 | 97.91 | 16.72 | 0.00 | 0.13 | 2.23 | 0.17 | 2.75 |
| Andorra | 1998 | 114.78 | 0.55 | 395.23 | 878.62 | 98.46 | 16.33 | 0.00 | 0.13 | 2.22 | 0.17 | 2.61 |
| Andorra | 1999 | 114.64 | 0.54 | 396.57 | 880.66 | 98.65 | 15.99 | 0.00 | 0.13 | 2.22 | 0.16 | 2.46 |
| Andorra | 2000 | 114.84 | 0.53 | 397.47 | 881.37 | 99.15 | 15.69 | 0.00 | 0.13 | 2.22 | 0.16 | 2.35 |
| Andorra | 2001 | 115.09 | 0.51 | 398.23 | 881.95 | 99.78 | 15.31 | 0.00 | 0.13 | 2.21 | 0.15 | 2.18 |
| Andorra | 2002 | 115.02 | 0.49 | 399.53 | 884.26 | 100.30 | 14.72 | 0.00 | 0.13 | 2.21 | 0.15 | 1.93 |
| Andorra | 2003 | 115.69 | 0.48 | 400.74 | 887.07 | 100.98 | 14.72 | 0.00 | 0.13 | 2.21 | 0.15 | 1.89 |
| Andorra | 2004 | 116.39 | 0.48 | 401.92 | 891.21 | 101.70 | 14.69 | 0.00 | 0.13 | 2.22 | 0.14 | 1.83 |
| Andorra | 2005 | 116.64 | 0.46 | 402.71 | 893.90 | 102.15 | 14.49 | 0.00 | 0.13 | 2.22 | 0.14 | 1.72 |
| Andorra | 2006 | 117.22 | 0.46 | 403.04 | 896.87 | 102.93 | 14.29 | 0.00 | 0.13 | 2.23 | 0.14 | 1.65 |
| Andorra | 2007 | 118.08 | 0.45 | 403.35 | 899.77 | 104.09 | 13.99 | 0.00 | 0.13 | 2.23 | 0.13 | 1.54 |
| Andorra | 2008 | 118.71 | 0.45 | 403.53 | 901.53 | 104.89 | 13.82 | 0.00 | 0.13 | 2.23 | 0.13 | 1.54 |
| Andorra | 2009 | 119.10 | 0.45 | 403.56 | 902.76 | 105.49 | 13.61 | 0.00 | 0.13 | 2.24 | 0.13 | 1.52 |
| Andorra | 2010 | 119.41 | 0.45 | 403.54 | 903.47 | 105.95 | 13.46 | 0.00 | 0.13 | 2.24 | 0.13 | 1.50 |
| Andorra | 2011 | 118.95 | 0.44 | 402.94 | 900.85 | 105.70 | 13.25 | 0.00 | 0.13 | 2.24 | 0.13 | 1.45 |
| Andorra | 2012 | 118.63 | 0.44 | 401.82 | 902.07 | 105.49 | 13.14 | 0.00 | 0.13 | 2.24 | 0.12 | 1.38 |
| Andorra | 2013 | 118.21 | 0.44 | 400.21 | 901.27 | 105.25 | 12.96 | 0.00 | 0.13 | 2.25 | 0.12 | 1.33 |
| Andorra | 2014 | 117.75 | 0.44 | 398.75 | 901.17 | 104.83 | 12.92 | 0.00 | 0.13 | 2.26 | 0.12 | 1.30 |
| Andorra | 2015 | 117.29 | 0.43 | 398.22 | 901.67 | 104.47 | 12.83 | 0.00 | 0.13 | 2.26 | 0.12 | 1.23 |
| Andorra | 2016 | 116.68 | 0.43 | 398.78 | 900.98 | 103.96 | 12.72 | 0.00 | 0.13 | 2.26 | 0.12 | 1.17 |
| Andorra | 2017 | 115.95 | 0.42 | 400.00 | 899.35 | 103.33 | 12.62 | 0.00 | 0.13 | 2.25 | 0.12 | 1.10 |
| Andorra | 2018 | 114.84 | 0.42 | 401.37 | 897.50 | 102.39 | 12.45 | 0.00 | 0.13 | 2.24 | 0.12 | 0.99 |
| Andorra | 2019 | 114.10 | 0.41 | 401.94 | 895.58 | 101.95 | 12.15 | 0.00 | 0.13 | 2.23 | 0.12 | 0.85 |
| Andorra | 2020 | 111.59 | 0.34 | 398.24 | 895.61 | 101.26 | 10.33 | 0.00 | 0.12 | 2.25 | 0.10 | 0.04 |
| Andorra | 2021 | 112.11 | 0.34 | 399.35 | 897.87 | 101.97 | 10.14 | 0.00 | 0.12 | 2.25 | 0.10 | 0.00 |
| Austria | 1990 | 231.97 | 2.05 | 329.20 | 890.17 | 148.64 | 83.33 | 0.01 | 0.26 | 2.70 | 0.56 | 27.92 |
| Austria | 1991 | 247.47 | 2.21 | 334.25 | 903.25 | 152.68 | 94.80 | 0.01 | 0.27 | 2.70 | 0.62 | 30.62 |
| Austria | 1992 | 264.47 | 2.41 | 339.54 | 915.40 | 156.51 | 107.95 | 0.01 | 0.29 | 2.70 | 0.69 | 33.79 |
| Austria | 1993 | 281.81 | 2.63 | 344.83 | 925.94 | 159.87 | 121.94 | 0.01 | 0.30 | 2.69 | 0.76 | 37.12 |
| Austria | 1994 | 294.92 | 2.83 | 349.92 | 934.37 | 162.35 | 132.57 | 0.01 | 0.32 | 2.67 | 0.82 | 39.70 |
| Austria | 1995 | 299.46 | 2.88 | 354.57 | 939.98 | 164.31 | 135.14 | 0.01 | 0.32 | 2.65 | 0.82 | 40.06 |
| Austria | 1996 | 298.44 | 2.85 | 360.11 | 944.88 | 165.72 | 132.71 | 0.01 | 0.32 | 2.62 | 0.80 | 39.11 |
| Austria | 1997 | 293.08 | 2.75 | 367.39 | 950.96 | 166.80 | 126.27 | 0.01 | 0.31 | 2.59 | 0.76 | 36.99 |
| Austria | 1998 | 292.17 | 2.72 | 375.42 | 957.25 | 168.16 | 124.01 | 0.01 | 0.31 | 2.55 | 0.74 | 35.97 |
| Austria | 1999 | 293.67 | 2.73 | 383.24 | 962.94 | 168.99 | 124.67 | 0.01 | 0.30 | 2.51 | 0.74 | 35.70 |
| Austria | 2000 | 297.55 | 2.72 | 389.84 | 966.97 | 169.64 | 127.91 | 0.01 | 0.31 | 2.48 | 0.75 | 35.88 |
| Austria | 2001 | 294.56 | 2.71 | 395.36 | 967.02 | 168.33 | 126.23 | 0.01 | 0.30 | 2.45 | 0.75 | 35.33 |
| Austria | 2002 | 293.44 | 2.76 | 400.50 | 963.07 | 165.52 | 127.92 | 0.01 | 0.30 | 2.40 | 0.77 | 35.80 |
| Austria | 2003 | 294.22 | 2.78 | 404.97 | 957.32 | 161.31 | 132.90 | 0.01 | 0.31 | 2.36 | 0.82 | 36.73 |
| Austria | 2004 | 295.86 | 2.87 | 408.53 | 952.08 | 157.61 | 138.25 | 0.01 | 0.31 | 2.33 | 0.88 | 38.13 |
| Austria | 2005 | 297.61 | 2.93 | 410.93 | 949.59 | 155.95 | 141.66 | 0.01 | 0.31 | 2.31 | 0.91 | 39.04 |
| Austria | 2006 | 298.62 | 2.93 | 412.61 | 952.09 | 156.95 | 141.67 | 0.01 | 0.31 | 2.31 | 0.90 | 38.87 |
| Austria | 2007 | 303.37 | 2.99 | 414.16 | 959.17 | 160.02 | 143.35 | 0.01 | 0.32 | 2.32 | 0.90 | 39.21 |
| Austria | 2008 | 309.49 | 3.06 | 415.51 | 968.74 | 164.04 | 145.45 | 0.01 | 0.32 | 2.33 | 0.89 | 39.62 |
| Austria | 2009 | 315.83 | 3.15 | 416.61 | 978.74 | 168.60 | 147.22 | 0.01 | 0.32 | 2.35 | 0.87 | 40.05 |
| Austria | 2010 | 317.88 | 3.14 | 417.38 | 987.04 | 172.06 | 145.83 | 0.01 | 0.32 | 2.36 | 0.85 | 39.51 |
| Austria | 2011 | 317.41 | 3.12 | 418.11 | 995.70 | 175.22 | 142.19 | 0.01 | 0.32 | 2.38 | 0.81 | 38.57 |
| Austria | 2012 | 313.65 | 3.04 | 419.02 | 1006.62 | 179.28 | 134.37 | 0.01 | 0.31 | 2.40 | 0.75 | 36.68 |
| Austria | 2013 | 308.12 | 2.93 | 420.03 | 1017.49 | 182.51 | 125.62 | 0.01 | 0.30 | 2.42 | 0.69 | 34.50 |
| Austria | 2014 | 303.58 | 2.83 | 421.06 | 1026.17 | 185.12 | 118.46 | 0.01 | 0.30 | 2.44 | 0.64 | 32.73 |
| Austria | 2015 | 302.87 | 2.83 | 422.06 | 1030.46 | 186.02 | 116.84 | 0.01 | 0.29 | 2.44 | 0.63 | 32.36 |
| Austria | 2016 | 296.53 | 2.79 | 424.23 | 1024.86 | 182.17 | 114.36 | 0.01 | 0.29 | 2.42 | 0.63 | 31.76 |
| Austria | 2017 | 289.68 | 2.93 | 427.87 | 1010.48 | 172.83 | 116.85 | 0.01 | 0.29 | 2.36 | 0.68 | 32.97 |
| Austria | 2018 | 288.69 | 3.21 | 431.64 | 995.10 | 162.93 | 125.76 | 0.01 | 0.29 | 2.31 | 0.77 | 35.97 |
| Austria | 2019 | 287.90 | 3.31 | 434.09 | 985.92 | 157.40 | 130.50 | 0.01 | 0.29 | 2.27 | 0.83 | 37.38 |
| Austria | 2020 | 278.05 | 3.13 | 434.10 | 977.56 | 157.47 | 120.57 | 0.01 | 0.28 | 2.25 | 0.77 | 35.00 |
| Austria | 2021 | 283.28 | 3.12 | 430.92 | 984.66 | 159.20 | 124.07 | 0.01 | 0.29 | 2.28 | 0.78 | 35.48 |
| Belarus | 1990 | 270.19 | 2.83 | 358.12 | 730.59 | 133.91 | 136.28 | 0.01 | 0.37 | 2.04 | 1.02 | 46.34 |
| Belarus | 1991 | 281.87 | 3.03 | 357.81 | 732.50 | 134.77 | 147.10 | 0.01 | 0.38 | 2.05 | 1.09 | 49.73 |
| Belarus | 1992 | 296.24 | 3.28 | 357.58 | 735.66 | 136.21 | 160.03 | 0.01 | 0.40 | 2.06 | 1.17 | 53.73 |
| Belarus | 1993 | 313.97 | 3.61 | 357.41 | 739.81 | 137.73 | 176.24 | 0.01 | 0.42 | 2.07 | 1.28 | 58.84 |
| Belarus | 1994 | 319.38 | 3.67 | 357.27 | 744.65 | 139.99 | 179.39 | 0.01 | 0.43 | 2.08 | 1.28 | 59.56 |
| Belarus | 1995 | 326.56 | 3.78 | 357.14 | 749.93 | 141.99 | 184.57 | 0.01 | 0.44 | 2.10 | 1.30 | 60.96 |
| Belarus | 1996 | 325.96 | 3.70 | 357.08 | 757.93 | 145.59 | 180.37 | 0.01 | 0.43 | 2.12 | 1.24 | 59.14 |
| Belarus | 1997 | 331.48 | 3.71 | 357.19 | 769.68 | 150.41 | 181.06 | 0.01 | 0.43 | 2.15 | 1.20 | 58.68 |
| Belarus | 1998 | 345.81 | 3.86 | 357.52 | 783.02 | 156.24 | 189.57 | 0.01 | 0.44 | 2.19 | 1.21 | 60.48 |
| Belarus | 1999 | 362.99 | 4.08 | 358.10 | 795.77 | 161.68 | 201.31 | 0.01 | 0.46 | 2.22 | 1.25 | 63.23 |
| Belarus | 2000 | 362.90 | 3.99 | 358.95 | 805.75 | 166.06 | 196.84 | 0.01 | 0.45 | 2.24 | 1.19 | 61.32 |
| Belarus | 2001 | 382.24 | 4.32 | 360.29 | 814.13 | 169.07 | 213.18 | 0.01 | 0.47 | 2.26 | 1.26 | 65.68 |
| Belarus | 2002 | 409.46 | 4.80 | 362.15 | 822.78 | 172.57 | 236.89 | 0.01 | 0.50 | 2.27 | 1.37 | 72.07 |
| Belarus | 2003 | 413.17 | 4.80 | 364.30 | 830.67 | 175.52 | 237.65 | 0.01 | 0.50 | 2.28 | 1.35 | 71.61 |
| Belarus | 2004 | 402.61 | 4.56 | 366.48 | 836.77 | 178.30 | 224.31 | 0.01 | 0.48 | 2.28 | 1.26 | 67.38 |
| Belarus | 2005 | 399.75 | 4.50 | 368.47 | 840.02 | 179.39 | 220.36 | 0.01 | 0.48 | 2.28 | 1.23 | 66.10 |
| Belarus | 2006 | 386.04 | 4.25 | 371.16 | 841.60 | 179.99 | 206.05 | 0.01 | 0.46 | 2.27 | 1.14 | 61.96 |
| Belarus | 2007 | 373.88 | 4.02 | 375.02 | 843.20 | 180.11 | 193.77 | 0.01 | 0.44 | 2.25 | 1.08 | 58.30 |
| Belarus | 2008 | 358.79 | 3.77 | 379.16 | 844.52 | 180.14 | 178.66 | 0.01 | 0.42 | 2.23 | 0.99 | 54.01 |
| Belarus | 2009 | 353.45 | 3.68 | 382.65 | 845.25 | 180.58 | 172.86 | 0.01 | 0.42 | 2.21 | 0.96 | 52.35 |
| Belarus | 2010 | 364.70 | 3.93 | 384.60 | 845.09 | 180.06 | 184.64 | 0.01 | 0.43 | 2.20 | 1.03 | 55.59 |
| Belarus | 2011 | 358.71 | 3.87 | 384.91 | 840.32 | 178.17 | 180.54 | 0.01 | 0.43 | 2.18 | 1.01 | 54.77 |
| Belarus | 2012 | 336.01 | 3.50 | 384.24 | 829.25 | 173.89 | 162.12 | 0.01 | 0.41 | 2.16 | 0.93 | 49.97 |
| Belarus | 2013 | 328.89 | 3.48 | 382.84 | 814.49 | 168.27 | 160.62 | 0.01 | 0.40 | 2.13 | 0.95 | 50.19 |
| Belarus | 2014 | 318.18 | 3.42 | 380.97 | 798.65 | 161.88 | 156.30 | 0.01 | 0.40 | 2.10 | 0.97 | 49.77 |
| Belarus | 2015 | 299.03 | 3.17 | 378.86 | 784.37 | 155.96 | 143.07 | 0.01 | 0.38 | 2.07 | 0.92 | 46.56 |
| Belarus | 2016 | 280.92 | 2.94 | 375.56 | 768.77 | 150.02 | 130.90 | 0.01 | 0.37 | 2.05 | 0.87 | 43.61 |
| Belarus | 2017 | 260.28 | 2.67 | 370.97 | 750.68 | 143.00 | 117.28 | 0.01 | 0.35 | 2.02 | 0.82 | 40.16 |
| Belarus | 2018 | 249.02 | 2.57 | 366.73 | 735.37 | 136.99 | 112.02 | 0.01 | 0.34 | 2.01 | 0.82 | 39.16 |
| Belarus | 2019 | 252.06 | 2.70 | 364.42 | 728.11 | 134.16 | 117.89 | 0.01 | 0.35 | 2.00 | 0.88 | 41.49 |
| Belarus | 2020 | 257.93 | 2.81 | 366.05 | 729.98 | 134.58 | 123.35 | 0.01 | 0.35 | 1.99 | 0.92 | 43.16 |
| Belarus | 2021 | 269.87 | 3.04 | 366.63 | 728.74 | 133.52 | 136.35 | 0.01 | 0.37 | 1.99 | 1.02 | 47.27 |
| Belgium | 1990 | 207.37 | 3.68 | 424.63 | 963.31 | 100.87 | 106.50 | 0.01 | 0.22 | 2.27 | 1.06 | 37.89 |
| Belgium | 1991 | 214.93 | 3.66 | 425.92 | 974.72 | 103.91 | 111.02 | 0.01 | 0.22 | 2.29 | 1.07 | 38.26 |
| Belgium | 1992 | 219.08 | 3.53 | 426.13 | 983.49 | 106.39 | 112.69 | 0.01 | 0.22 | 2.31 | 1.06 | 37.61 |
| Belgium | 1993 | 219.61 | 3.33 | 425.36 | 989.46 | 108.33 | 111.28 | 0.01 | 0.22 | 2.33 | 1.03 | 36.08 |
| Belgium | 1994 | 222.95 | 3.27 | 423.74 | 992.59 | 109.84 | 113.11 | 0.01 | 0.22 | 2.34 | 1.03 | 36.05 |
| Belgium | 1995 | 223.16 | 3.16 | 421.40 | 992.75 | 110.41 | 112.75 | 0.01 | 0.22 | 2.36 | 1.02 | 35.45 |
| Belgium | 1996 | 225.00 | 3.18 | 416.42 | 986.90 | 110.14 | 114.86 | 0.01 | 0.23 | 2.37 | 1.04 | 36.31 |
| Belgium | 1997 | 223.10 | 3.09 | 408.28 | 975.11 | 109.41 | 113.69 | 0.01 | 0.23 | 2.39 | 1.04 | 36.18 |
| Belgium | 1998 | 216.39 | 2.82 | 399.14 | 961.66 | 108.45 | 107.93 | 0.01 | 0.23 | 2.41 | 1.00 | 34.14 |
| Belgium | 1999 | 216.90 | 2.79 | 391.19 | 950.92 | 107.55 | 109.35 | 0.01 | 0.23 | 2.43 | 1.02 | 34.79 |
| Belgium | 2000 | 213.26 | 2.57 | 386.61 | 947.22 | 107.05 | 106.21 | 0.01 | 0.23 | 2.45 | 0.99 | 33.19 |
| Belgium | 2001 | 212.03 | 2.54 | 384.35 | 948.94 | 107.12 | 104.91 | 0.01 | 0.22 | 2.47 | 0.98 | 32.77 |
| Belgium | 2002 | 210.14 | 2.54 | 382.06 | 951.31 | 107.55 | 102.59 | 0.01 | 0.22 | 2.49 | 0.95 | 32.28 |
| Belgium | 2003 | 211.03 | 2.60 | 380.03 | 953.96 | 108.36 | 102.67 | 0.01 | 0.22 | 2.51 | 0.95 | 32.65 |
| Belgium | 2004 | 213.77 | 2.78 | 378.55 | 956.55 | 109.37 | 104.40 | 0.01 | 0.22 | 2.53 | 0.95 | 33.95 |
| Belgium | 2005 | 216.50 | 2.86 | 377.90 | 958.78 | 109.83 | 106.66 | 0.01 | 0.23 | 2.54 | 0.97 | 34.83 |
| Belgium | 2006 | 217.74 | 2.82 | 377.83 | 959.24 | 110.68 | 107.06 | 0.01 | 0.23 | 2.54 | 0.97 | 34.67 |
| Belgium | 2007 | 222.64 | 2.89 | 377.97 | 957.85 | 111.45 | 111.20 | 0.01 | 0.23 | 2.53 | 1.00 | 35.92 |
| Belgium | 2008 | 226.00 | 2.96 | 378.39 | 956.28 | 112.51 | 113.50 | 0.01 | 0.24 | 2.53 | 1.01 | 36.74 |
| Belgium | 2009 | 224.85 | 2.87 | 379.14 | 956.24 | 113.69 | 111.16 | 0.01 | 0.24 | 2.52 | 0.98 | 35.61 |
| Belgium | 2010 | 226.76 | 2.88 | 380.28 | 959.50 | 115.61 | 111.15 | 0.01 | 0.24 | 2.52 | 0.96 | 35.45 |
| Belgium | 2011 | 227.93 | 2.88 | 382.90 | 968.55 | 119.29 | 108.64 | 0.01 | 0.24 | 2.53 | 0.91 | 34.47 |
| Belgium | 2012 | 229.40 | 2.77 | 387.24 | 982.39 | 124.63 | 104.76 | 0.01 | 0.23 | 2.54 | 0.84 | 32.49 |
| Belgium | 2013 | 234.32 | 2.79 | 392.16 | 997.40 | 129.93 | 104.40 | 0.01 | 0.23 | 2.54 | 0.80 | 31.89 |
| Belgium | 2014 | 239.43 | 2.77 | 396.48 | 1009.86 | 134.52 | 104.92 | 0.01 | 0.24 | 2.55 | 0.78 | 31.40 |
| Belgium | 2015 | 244.20 | 2.84 | 399.02 | 1016.07 | 136.91 | 107.29 | 0.01 | 0.24 | 2.55 | 0.78 | 31.97 |
| Belgium | 2016 | 247.01 | 2.94 | 399.49 | 1010.95 | 136.19 | 110.82 | 0.01 | 0.24 | 2.53 | 0.81 | 33.22 |
| Belgium | 2017 | 253.82 | 3.35 | 398.86 | 996.72 | 134.21 | 119.61 | 0.01 | 0.25 | 2.50 | 0.89 | 37.38 |
| Belgium | 2018 | 266.43 | 4.05 | 397.96 | 981.12 | 131.93 | 134.50 | 0.01 | 0.27 | 2.47 | 1.02 | 44.36 |
| Belgium | 2019 | 263.92 | 4.00 | 397.61 | 971.72 | 129.70 | 134.22 | 0.01 | 0.27 | 2.44 | 1.03 | 44.38 |
| Belgium | 2020 | 248.84 | 3.69 | 396.69 | 987.36 | 127.84 | 121.00 | 0.01 | 0.25 | 2.49 | 0.95 | 40.03 |
| Belgium | 2021 | 265.30 | 3.96 | 396.09 | 987.31 | 126.61 | 138.69 | 0.01 | 0.27 | 2.49 | 1.10 | 44.98 |
| Bosnia and Herzegovina | 1990 | 225.33 | 4.42 | 587.02 | 420.17 | 55.11 | 170.22 | 0.01 | 0.54 | 0.72 | 3.09 | 89.52 |
| Bosnia and Herzegovina | 1991 | 241.21 | 4.76 | 574.27 | 418.54 | 54.93 | 186.28 | 0.01 | 0.58 | 0.73 | 3.39 | 98.59 |
| Bosnia and Herzegovina | 1992 | 241.97 | 4.77 | 562.47 | 417.01 | 54.59 | 187.38 | 0.01 | 0.58 | 0.74 | 3.43 | 99.91 |
| Bosnia and Herzegovina | 1993 | 237.65 | 4.75 | 551.81 | 415.41 | 54.54 | 183.11 | 0.01 | 0.57 | 0.75 | 3.36 | 98.47 |
| Bosnia and Herzegovina | 1994 | 238.19 | 4.76 | 542.57 | 413.55 | 54.26 | 183.93 | 0.01 | 0.58 | 0.76 | 3.39 | 99.58 |
| Bosnia and Herzegovina | 1995 | 237.45 | 4.70 | 535.87 | 411.96 | 53.80 | 183.65 | 0.01 | 0.58 | 0.77 | 3.41 | 100.00 |
| Bosnia and Herzegovina | 1996 | 227.51 | 4.50 | 531.24 | 406.98 | 53.81 | 173.70 | 0.01 | 0.56 | 0.77 | 3.23 | 95.26 |
| Bosnia and Herzegovina | 1997 | 216.89 | 4.29 | 527.43 | 397.06 | 53.53 | 163.36 | 0.01 | 0.55 | 0.75 | 3.05 | 90.91 |
| Bosnia and Herzegovina | 1998 | 205.75 | 4.01 | 524.62 | 386.36 | 53.67 | 152.08 | 0.01 | 0.53 | 0.74 | 2.83 | 85.52 |
| Bosnia and Herzegovina | 1999 | 193.93 | 3.79 | 521.83 | 378.00 | 53.83 | 140.11 | 0.01 | 0.51 | 0.72 | 2.60 | 79.77 |
| Bosnia and Herzegovina | 2000 | 188.61 | 3.68 | 517.81 | 374.77 | 54.02 | 134.58 | 0.01 | 0.50 | 0.72 | 2.49 | 77.06 |
| Bosnia and Herzegovina | 2001 | 179.82 | 3.53 | 509.29 | 374.90 | 54.14 | 125.68 | 0.01 | 0.48 | 0.74 | 2.32 | 72.38 |
| Bosnia and Herzegovina | 2002 | 179.13 | 3.51 | 495.39 | 374.66 | 54.40 | 124.73 | 0.01 | 0.48 | 0.76 | 2.29 | 72.18 |
| Bosnia and Herzegovina | 2003 | 173.52 | 3.43 | 479.08 | 374.45 | 54.63 | 118.89 | 0.01 | 0.46 | 0.78 | 2.18 | 69.50 |
| Bosnia and Herzegovina | 2004 | 168.51 | 3.35 | 463.33 | 374.70 | 55.16 | 113.35 | 0.01 | 0.45 | 0.81 | 2.06 | 66.82 |
| Bosnia and Herzegovina | 2005 | 168.70 | 3.40 | 451.00 | 375.75 | 55.66 | 113.05 | 0.01 | 0.45 | 0.83 | 2.03 | 67.04 |
| Bosnia and Herzegovina | 2006 | 167.35 | 3.35 | 440.62 | 377.97 | 56.90 | 110.45 | 0.01 | 0.44 | 0.86 | 1.94 | 65.35 |
| Bosnia and Herzegovina | 2007 | 171.14 | 3.49 | 429.93 | 381.36 | 58.23 | 112.91 | 0.01 | 0.45 | 0.89 | 1.94 | 66.81 |
| Bosnia and Herzegovina | 2008 | 165.65 | 3.32 | 420.52 | 385.66 | 59.75 | 105.90 | 0.01 | 0.43 | 0.92 | 1.77 | 62.41 |
| Bosnia and Herzegovina | 2009 | 164.38 | 3.28 | 413.68 | 389.85 | 61.42 | 102.96 | 0.01 | 0.42 | 0.94 | 1.68 | 60.44 |
| Bosnia and Herzegovina | 2010 | 162.60 | 3.19 | 410.74 | 392.94 | 62.84 | 99.76 | 0.01 | 0.41 | 0.96 | 1.59 | 58.15 |
| Bosnia and Herzegovina | 2011 | 160.29 | 3.12 | 410.43 | 395.66 | 63.94 | 96.35 | 0.01 | 0.41 | 0.96 | 1.51 | 55.99 |
| Bosnia and Herzegovina | 2012 | 160.74 | 3.10 | 410.43 | 398.73 | 65.57 | 95.16 | 0.01 | 0.40 | 0.97 | 1.45 | 54.86 |
| Bosnia and Herzegovina | 2013 | 163.05 | 3.13 | 410.68 | 401.68 | 67.24 | 95.81 | 0.01 | 0.41 | 0.98 | 1.42 | 54.78 |
| Bosnia and Herzegovina | 2014 | 162.71 | 3.11 | 411.14 | 403.99 | 68.35 | 94.36 | 0.01 | 0.40 | 0.98 | 1.38 | 53.75 |
| Bosnia and Herzegovina | 2015 | 166.15 | 3.17 | 411.72 | 405.09 | 68.78 | 97.37 | 0.01 | 0.41 | 0.98 | 1.42 | 55.10 |
| Bosnia and Herzegovina | 2016 | 164.66 | 3.15 | 412.28 | 401.68 | 67.13 | 97.53 | 0.01 | 0.41 | 0.97 | 1.45 | 55.55 |
| Bosnia and Herzegovina | 2017 | 158.28 | 3.07 | 412.79 | 393.83 | 63.54 | 94.73 | 0.01 | 0.40 | 0.95 | 1.49 | 55.14 |
| Bosnia and Herzegovina | 2018 | 152.76 | 3.01 | 413.36 | 386.04 | 60.22 | 92.54 | 0.01 | 0.40 | 0.93 | 1.54 | 55.13 |
| Bosnia and Herzegovina | 2019 | 151.23 | 3.03 | 414.11 | 382.84 | 58.43 | 92.80 | 0.01 | 0.40 | 0.92 | 1.59 | 55.94 |
| Bosnia and Herzegovina | 2020 | 144.73 | 2.91 | 415.27 | 384.52 | 58.67 | 86.06 | 0.01 | 0.38 | 0.93 | 1.47 | 52.12 |
| Bosnia and Herzegovina | 2021 | 137.10 | 2.68 | 416.35 | 382.98 | 58.21 | 78.90 | 0.01 | 0.36 | 0.92 | 1.36 | 47.89 |
| Bulgaria | 1990 | 109.01 | 0.79 | 369.82 | 612.65 | 74.75 | 34.26 | 0.00 | 0.18 | 1.66 | 0.46 | 12.13 |
| Bulgaria | 1991 | 110.26 | 0.79 | 371.68 | 618.53 | 75.35 | 34.91 | 0.00 | 0.18 | 1.66 | 0.46 | 12.20 |
| Bulgaria | 1992 | 111.31 | 0.78 | 373.56 | 624.48 | 76.17 | 35.14 | 0.00 | 0.18 | 1.67 | 0.46 | 12.11 |
| Bulgaria | 1993 | 114.68 | 0.86 | 375.44 | 630.41 | 77.17 | 37.51 | 0.00 | 0.18 | 1.68 | 0.49 | 13.16 |
| Bulgaria | 1994 | 118.01 | 0.91 | 377.28 | 636.22 | 78.17 | 39.84 | 0.00 | 0.19 | 1.69 | 0.51 | 14.05 |
| Bulgaria | 1995 | 117.79 | 0.89 | 379.05 | 641.79 | 78.98 | 38.81 | 0.00 | 0.18 | 1.69 | 0.49 | 13.49 |
| Bulgaria | 1996 | 118.20 | 0.88 | 380.91 | 647.96 | 79.84 | 38.36 | 0.00 | 0.18 | 1.70 | 0.48 | 13.14 |
| Bulgaria | 1997 | 121.05 | 0.90 | 382.97 | 655.03 | 81.13 | 39.92 | 0.00 | 0.18 | 1.71 | 0.49 | 13.61 |
| Bulgaria | 1998 | 123.69 | 0.93 | 385.08 | 662.08 | 82.32 | 41.37 | 0.00 | 0.19 | 1.72 | 0.50 | 14.02 |
| Bulgaria | 1999 | 127.26 | 0.98 | 387.10 | 668.31 | 83.14 | 44.12 | 0.00 | 0.19 | 1.73 | 0.53 | 14.98 |
| Bulgaria | 2000 | 130.88 | 1.04 | 388.87 | 672.82 | 84.26 | 46.62 | 0.00 | 0.19 | 1.73 | 0.55 | 15.87 |
| Bulgaria | 2001 | 132.23 | 1.06 | 391.03 | 677.82 | 85.19 | 47.04 | 0.00 | 0.20 | 1.73 | 0.55 | 15.97 |
| Bulgaria | 2002 | 133.42 | 1.06 | 394.04 | 684.95 | 86.87 | 46.55 | 0.00 | 0.19 | 1.74 | 0.54 | 15.66 |
| Bulgaria | 2003 | 135.58 | 1.07 | 397.48 | 692.59 | 88.21 | 47.38 | 0.00 | 0.20 | 1.74 | 0.54 | 15.75 |
| Bulgaria | 2004 | 138.65 | 1.10 | 400.92 | 699.05 | 89.52 | 49.13 | 0.00 | 0.20 | 1.74 | 0.55 | 16.21 |
| Bulgaria | 2005 | 141.51 | 1.14 | 403.92 | 702.65 | 90.24 | 51.27 | 0.00 | 0.20 | 1.74 | 0.57 | 16.91 |
| Bulgaria | 2006 | 143.66 | 1.17 | 407.08 | 703.82 | 90.57 | 53.09 | 0.00 | 0.20 | 1.73 | 0.59 | 17.49 |
| Bulgaria | 2007 | 146.89 | 1.21 | 410.87 | 704.29 | 90.72 | 56.17 | 0.00 | 0.21 | 1.71 | 0.62 | 18.48 |
| Bulgaria | 2008 | 147.15 | 1.23 | 414.75 | 704.40 | 90.80 | 56.35 | 0.00 | 0.21 | 1.70 | 0.62 | 18.54 |
| Bulgaria | 2009 | 145.21 | 1.20 | 418.16 | 704.38 | 90.84 | 54.37 | 0.00 | 0.21 | 1.68 | 0.60 | 17.84 |
| Bulgaria | 2010 | 143.78 | 1.18 | 420.55 | 704.51 | 91.17 | 52.61 | 0.00 | 0.20 | 1.68 | 0.58 | 17.22 |
| Bulgaria | 2011 | 140.19 | 1.11 | 422.01 | 703.92 | 91.23 | 48.96 | 0.00 | 0.20 | 1.67 | 0.54 | 15.87 |
| Bulgaria | 2012 | 139.82 | 1.12 | 423.14 | 702.18 | 91.29 | 48.53 | 0.00 | 0.20 | 1.66 | 0.53 | 15.84 |
| Bulgaria | 2013 | 137.93 | 1.08 | 424.11 | 700.25 | 91.43 | 46.50 | 0.00 | 0.20 | 1.65 | 0.51 | 15.12 |
| Bulgaria | 2014 | 137.96 | 1.08 | 425.09 | 699.12 | 91.95 | 46.01 | 0.00 | 0.20 | 1.64 | 0.50 | 14.98 |
| Bulgaria | 2015 | 138.15 | 1.08 | 426.26 | 699.82 | 92.45 | 45.70 | 0.00 | 0.20 | 1.64 | 0.49 | 14.90 |
| Bulgaria | 2016 | 137.94 | 1.06 | 427.77 | 701.86 | 93.28 | 44.66 | 0.00 | 0.20 | 1.64 | 0.48 | 14.48 |
| Bulgaria | 2017 | 140.51 | 1.10 | 429.54 | 704.13 | 94.84 | 45.67 | 0.00 | 0.20 | 1.64 | 0.48 | 14.88 |
| Bulgaria | 2018 | 144.38 | 1.17 | 431.36 | 706.65 | 96.03 | 48.35 | 0.00 | 0.20 | 1.64 | 0.50 | 15.87 |
| Bulgaria | 2019 | 146.52 | 1.18 | 433.04 | 709.44 | 96.94 | 49.57 | 0.00 | 0.21 | 1.64 | 0.51 | 16.21 |
| Bulgaria | 2020 | 143.82 | 1.14 | 435.57 | 711.91 | 96.75 | 47.07 | 0.00 | 0.20 | 1.63 | 0.49 | 15.26 |
| Bulgaria | 2021 | 146.99 | 1.18 | 441.33 | 748.31 | 97.29 | 49.71 | 0.00 | 0.20 | 1.70 | 0.51 | 15.37 |
| Croatia | 1990 | 152.43 | 1.59 | 450.48 | 762.21 | 86.67 | 65.77 | 0.00 | 0.20 | 1.69 | 0.76 | 21.34 |
| Croatia | 1991 | 158.23 | 1.64 | 449.82 | 775.47 | 90.33 | 67.91 | 0.00 | 0.20 | 1.72 | 0.75 | 21.72 |
| Croatia | 1992 | 161.78 | 1.60 | 449.49 | 788.44 | 94.30 | 67.49 | 0.00 | 0.21 | 1.75 | 0.72 | 21.03 |
| Croatia | 1993 | 166.53 | 1.64 | 449.52 | 800.98 | 98.26 | 68.27 | 0.00 | 0.21 | 1.78 | 0.69 | 21.12 |
| Croatia | 1994 | 171.91 | 1.73 | 449.91 | 812.66 | 102.33 | 69.59 | 0.00 | 0.21 | 1.81 | 0.68 | 21.52 |
| Croatia | 1995 | 176.37 | 1.74 | 450.61 | 823.14 | 106.41 | 69.96 | 0.00 | 0.21 | 1.83 | 0.66 | 21.40 |
| Croatia | 1996 | 180.96 | 1.73 | 451.84 | 834.00 | 111.31 | 69.65 | 0.00 | 0.22 | 1.85 | 0.63 | 21.02 |
| Croatia | 1997 | 186.97 | 1.72 | 453.72 | 846.11 | 116.90 | 70.06 | 0.00 | 0.22 | 1.86 | 0.60 | 20.80 |
| Croatia | 1998 | 196.94 | 1.79 | 456.11 | 858.11 | 123.22 | 73.72 | 0.00 | 0.23 | 1.88 | 0.60 | 21.66 |
| Croatia | 1999 | 208.96 | 1.93 | 458.87 | 868.53 | 127.80 | 81.16 | 0.00 | 0.24 | 1.89 | 0.64 | 23.56 |
| Croatia | 2000 | 217.13 | 1.99 | 461.87 | 875.95 | 131.31 | 85.82 | 0.00 | 0.25 | 1.90 | 0.65 | 24.60 |
| Croatia | 2001 | 225.01 | 2.10 | 466.20 | 882.15 | 133.55 | 91.46 | 0.00 | 0.26 | 1.89 | 0.68 | 26.01 |
| Croatia | 2002 | 231.48 | 2.18 | 472.61 | 889.42 | 136.17 | 95.31 | 0.00 | 0.26 | 1.88 | 0.70 | 26.84 |
| Croatia | 2003 | 238.43 | 2.26 | 480.39 | 897.14 | 139.21 | 99.22 | 0.00 | 0.27 | 1.87 | 0.71 | 27.60 |
| Croatia | 2004 | 243.07 | 2.29 | 488.85 | 904.72 | 141.88 | 101.18 | 0.00 | 0.27 | 1.85 | 0.71 | 27.78 |
| Croatia | 2005 | 250.95 | 2.39 | 497.29 | 911.48 | 144.36 | 106.60 | 0.00 | 0.28 | 1.83 | 0.74 | 28.87 |
| Croatia | 2006 | 252.24 | 2.37 | 507.93 | 918.70 | 146.75 | 105.49 | 0.00 | 0.27 | 1.81 | 0.72 | 28.24 |
| Croatia | 2007 | 261.92 | 2.52 | 521.70 | 927.24 | 150.09 | 111.83 | 0.00 | 0.28 | 1.78 | 0.75 | 29.53 |
| Croatia | 2008 | 266.23 | 2.58 | 535.91 | 935.73 | 152.58 | 113.65 | 0.00 | 0.28 | 1.75 | 0.74 | 29.67 |
| Croatia | 2009 | 261.61 | 2.47 | 547.96 | 943.04 | 155.41 | 106.19 | 0.00 | 0.28 | 1.72 | 0.68 | 27.55 |
| Croatia | 2010 | 256.48 | 2.37 | 555.17 | 947.87 | 156.88 | 99.60 | 0.00 | 0.27 | 1.71 | 0.63 | 25.83 |
| Croatia | 2011 | 248.54 | 2.19 | 558.73 | 949.74 | 158.10 | 90.44 | 0.00 | 0.26 | 1.70 | 0.57 | 23.47 |
| Croatia | 2012 | 246.19 | 2.18 | 561.42 | 949.01 | 158.06 | 88.13 | 0.00 | 0.26 | 1.69 | 0.56 | 23.03 |
| Croatia | 2013 | 239.35 | 2.03 | 563.10 | 945.82 | 157.18 | 82.17 | 0.00 | 0.25 | 1.68 | 0.52 | 21.44 |
| Croatia | 2014 | 235.11 | 1.97 | 563.67 | 940.57 | 155.26 | 79.84 | 0.00 | 0.25 | 1.67 | 0.51 | 20.88 |
| Croatia | 2015 | 233.27 | 2.02 | 563.00 | 933.53 | 153.02 | 80.25 | 0.00 | 0.25 | 1.66 | 0.52 | 21.22 |
| Croatia | 2016 | 225.51 | 1.93 | 560.13 | 918.42 | 148.89 | 76.62 | 0.00 | 0.25 | 1.64 | 0.51 | 20.49 |
| Croatia | 2017 | 221.09 | 2.00 | 555.01 | 894.49 | 142.42 | 78.67 | 0.00 | 0.25 | 1.61 | 0.55 | 21.53 |
| Croatia | 2018 | 217.06 | 2.04 | 548.88 | 870.42 | 135.89 | 81.17 | 0.00 | 0.25 | 1.59 | 0.60 | 22.61 |
| Croatia | 2019 | 213.24 | 2.04 | 543.00 | 855.07 | 130.81 | 82.43 | 0.00 | 0.25 | 1.57 | 0.63 | 23.24 |
| Croatia | 2020 | 204.09 | 1.93 | 530.17 | 849.85 | 127.54 | 76.55 | 0.00 | 0.24 | 1.60 | 0.60 | 21.85 |
| Croatia | 2021 | 200.74 | 1.95 | 529.15 | 838.34 | 123.73 | 77.01 | 0.00 | 0.24 | 1.58 | 0.62 | 22.25 |
| Cyprus | 1990 | 216.61 | 4.73 | 359.43 | 688.84 | 91.20 | 125.41 | 0.01 | 0.31 | 1.92 | 1.38 | 59.36 |
| Cyprus | 1991 | 217.24 | 4.79 | 355.85 | 673.99 | 91.26 | 125.97 | 0.01 | 0.32 | 1.89 | 1.38 | 60.63 |
| Cyprus | 1992 | 217.40 | 4.80 | 352.35 | 660.55 | 91.33 | 126.06 | 0.01 | 0.33 | 1.87 | 1.38 | 61.43 |
| Cyprus | 1993 | 216.40 | 4.67 | 349.05 | 649.05 | 91.53 | 124.87 | 0.01 | 0.33 | 1.86 | 1.36 | 61.02 |
| Cyprus | 1994 | 213.92 | 4.37 | 346.10 | 639.97 | 92.00 | 121.93 | 0.01 | 0.33 | 1.85 | 1.33 | 58.86 |
| Cyprus | 1995 | 212.56 | 4.20 | 343.63 | 633.78 | 92.08 | 120.48 | 0.01 | 0.34 | 1.84 | 1.31 | 57.77 |
| Cyprus | 1996 | 211.29 | 4.00 | 341.08 | 629.95 | 92.55 | 118.73 | 0.01 | 0.34 | 1.85 | 1.28 | 56.32 |
| Cyprus | 1997 | 211.67 | 3.94 | 337.98 | 627.58 | 93.53 | 118.14 | 0.01 | 0.34 | 1.86 | 1.26 | 56.02 |
| Cyprus | 1998 | 211.32 | 3.88 | 334.64 | 626.59 | 94.50 | 116.81 | 0.01 | 0.34 | 1.87 | 1.24 | 55.43 |
| Cyprus | 1999 | 209.69 | 3.70 | 331.36 | 626.89 | 94.94 | 114.75 | 0.01 | 0.33 | 1.89 | 1.21 | 53.90 |
| Cyprus | 2000 | 208.84 | 3.59 | 328.44 | 628.37 | 95.48 | 113.36 | 0.01 | 0.33 | 1.91 | 1.19 | 52.90 |
| Cyprus | 2001 | 205.94 | 3.46 | 325.03 | 629.87 | 96.09 | 109.85 | 0.01 | 0.33 | 1.94 | 1.14 | 51.20 |
| Cyprus | 2002 | 202.23 | 3.34 | 320.67 | 630.39 | 95.98 | 106.25 | 0.01 | 0.32 | 1.97 | 1.11 | 49.71 |
| Cyprus | 2003 | 199.35 | 3.20 | 316.18 | 630.11 | 95.71 | 103.64 | 0.01 | 0.32 | 1.99 | 1.08 | 48.45 |
| Cyprus | 2004 | 196.09 | 3.00 | 312.40 | 629.24 | 95.46 | 100.63 | 0.01 | 0.31 | 2.01 | 1.05 | 46.51 |
| Cyprus | 2005 | 198.56 | 2.96 | 310.14 | 627.98 | 94.68 | 103.88 | 0.01 | 0.32 | 2.02 | 1.10 | 47.42 |
| Cyprus | 2006 | 195.30 | 2.91 | 308.83 | 624.61 | 93.90 | 101.40 | 0.01 | 0.31 | 2.02 | 1.08 | 46.59 |
| Cyprus | 2007 | 191.35 | 2.85 | 307.45 | 618.48 | 92.05 | 99.30 | 0.01 | 0.31 | 2.01 | 1.08 | 46.06 |
| Cyprus | 2008 | 189.71 | 2.85 | 306.20 | 611.32 | 89.87 | 99.84 | 0.01 | 0.31 | 2.00 | 1.11 | 46.71 |
| Cyprus | 2009 | 187.23 | 2.85 | 305.24 | 604.92 | 88.04 | 99.19 | 0.01 | 0.31 | 1.98 | 1.13 | 46.98 |
| Cyprus | 2010 | 184.17 | 2.85 | 304.77 | 601.02 | 87.27 | 96.90 | 0.01 | 0.31 | 1.97 | 1.11 | 46.54 |
| Cyprus | 2011 | 179.06 | 2.76 | 305.22 | 600.19 | 87.27 | 91.79 | 0.01 | 0.30 | 1.97 | 1.05 | 44.42 |
| Cyprus | 2012 | 176.87 | 2.70 | 306.66 | 601.45 | 87.97 | 88.90 | 0.01 | 0.29 | 1.96 | 1.01 | 42.97 |
| Cyprus | 2013 | 173.81 | 2.63 | 308.80 | 604.28 | 88.95 | 84.86 | 0.01 | 0.29 | 1.96 | 0.95 | 40.98 |
| Cyprus | 2014 | 174.42 | 2.61 | 311.31 | 608.14 | 90.02 | 84.40 | 0.01 | 0.29 | 1.95 | 0.94 | 40.39 |
| Cyprus | 2015 | 175.86 | 2.61 | 313.86 | 612.47 | 91.09 | 84.76 | 0.01 | 0.29 | 1.95 | 0.93 | 40.15 |
| Cyprus | 2016 | 176.06 | 2.54 | 317.48 | 619.27 | 93.32 | 82.74 | 0.01 | 0.28 | 1.95 | 0.89 | 38.63 |
| Cyprus | 2017 | 181.41 | 2.61 | 322.36 | 628.66 | 95.88 | 85.54 | 0.01 | 0.29 | 1.95 | 0.89 | 39.19 |
| Cyprus | 2018 | 183.67 | 2.57 | 326.95 | 637.33 | 98.19 | 85.48 | 0.01 | 0.29 | 1.95 | 0.87 | 38.34 |
| Cyprus | 2019 | 185.26 | 2.55 | 329.67 | 642.01 | 99.59 | 85.66 | 0.01 | 0.29 | 1.95 | 0.86 | 37.95 |
| Cyprus | 2020 | 174.33 | 2.29 | 328.95 | 643.47 | 99.57 | 74.76 | 0.01 | 0.27 | 1.96 | 0.75 | 33.40 |
| Cyprus | 2021 | 176.99 | 2.29 | 328.31 | 642.31 | 99.55 | 77.44 | 0.01 | 0.28 | 1.96 | 0.78 | 34.13 |
| Czechia | 1990 | 124.68 | 1.10 | 468.67 | 986.92 | 80.46 | 44.22 | 0.00 | 0.13 | 2.11 | 0.55 | 10.37 |
| Czechia | 1991 | 125.93 | 1.10 | 471.23 | 1022.92 | 81.89 | 44.03 | 0.00 | 0.12 | 2.17 | 0.54 | 9.89 |
| Czechia | 1992 | 124.73 | 1.03 | 473.83 | 1055.43 | 82.70 | 42.03 | 0.00 | 0.12 | 2.23 | 0.51 | 8.77 |
| Czechia | 1993 | 126.29 | 1.05 | 476.31 | 1082.86 | 83.84 | 42.46 | 0.00 | 0.12 | 2.27 | 0.51 | 8.67 |
| Czechia | 1994 | 129.58 | 1.13 | 478.49 | 1103.57 | 84.55 | 45.03 | 0.00 | 0.12 | 2.31 | 0.53 | 9.48 |
| Czechia | 1995 | 129.52 | 1.11 | 480.19 | 1115.95 | 85.09 | 44.43 | 0.00 | 0.12 | 2.32 | 0.52 | 9.10 |
| Czechia | 1996 | 129.07 | 1.09 | 482.08 | 1121.38 | 85.21 | 43.86 | 0.00 | 0.12 | 2.33 | 0.51 | 8.83 |
| Czechia | 1997 | 130.10 | 1.11 | 484.89 | 1123.41 | 85.22 | 44.88 | 0.00 | 0.12 | 2.32 | 0.53 | 9.11 |
| Czechia | 1998 | 130.00 | 1.12 | 488.56 | 1123.76 | 85.05 | 44.95 | 0.00 | 0.12 | 2.30 | 0.53 | 9.13 |
| Czechia | 1999 | 133.36 | 1.20 | 493.02 | 1124.09 | 84.92 | 48.44 | 0.00 | 0.12 | 2.28 | 0.57 | 10.30 |
| Czechia | 2000 | 135.65 | 1.23 | 498.20 | 1126.09 | 85.47 | 50.18 | 0.00 | 0.12 | 2.26 | 0.59 | 10.79 |
| Czechia | 2001 | 138.12 | 1.28 | 507.04 | 1130.72 | 86.66 | 51.46 | 0.00 | 0.12 | 2.23 | 0.59 | 11.13 |
| Czechia | 2002 | 139.27 | 1.29 | 520.82 | 1137.32 | 88.17 | 51.10 | 0.00 | 0.12 | 2.18 | 0.58 | 10.83 |
| Czechia | 2003 | 141.90 | 1.31 | 536.91 | 1145.23 | 90.21 | 51.69 | 0.00 | 0.12 | 2.13 | 0.57 | 10.74 |
| Czechia | 2004 | 143.18 | 1.30 | 552.73 | 1153.88 | 92.19 | 51.00 | 0.00 | 0.12 | 2.09 | 0.55 | 10.27 |
| Czechia | 2005 | 145.46 | 1.32 | 565.68 | 1162.67 | 94.40 | 51.06 | 0.00 | 0.13 | 2.06 | 0.54 | 10.12 |
| Czechia | 2006 | 147.65 | 1.30 | 577.25 | 1174.61 | 97.72 | 49.93 | 0.00 | 0.13 | 2.03 | 0.51 | 9.52 |
| Czechia | 2007 | 155.42 | 1.36 | 589.67 | 1191.06 | 103.08 | 52.35 | 0.00 | 0.13 | 2.02 | 0.51 | 9.94 |
| Czechia | 2008 | 161.75 | 1.41 | 601.46 | 1209.05 | 108.82 | 52.93 | 0.00 | 0.13 | 2.01 | 0.49 | 9.92 |
| Czechia | 2009 | 166.71 | 1.41 | 611.09 | 1225.68 | 113.84 | 52.87 | 0.00 | 0.14 | 2.01 | 0.46 | 9.66 |
| Czechia | 2010 | 169.91 | 1.43 | 617.07 | 1238.08 | 116.87 | 53.05 | 0.00 | 0.14 | 2.01 | 0.45 | 9.59 |
| Czechia | 2011 | 170.75 | 1.43 | 620.10 | 1248.16 | 119.18 | 51.56 | 0.00 | 0.14 | 2.01 | 0.43 | 9.19 |
| Czechia | 2012 | 175.76 | 1.55 | 621.89 | 1258.54 | 121.33 | 54.44 | 0.00 | 0.14 | 2.02 | 0.45 | 10.05 |
| Czechia | 2013 | 178.43 | 1.60 | 622.43 | 1267.26 | 123.25 | 55.19 | 0.00 | 0.14 | 2.04 | 0.45 | 10.28 |
| Czechia | 2014 | 177.90 | 1.56 | 621.70 | 1272.37 | 124.73 | 53.17 | 0.00 | 0.14 | 2.05 | 0.43 | 9.74 |
| Czechia | 2015 | 180.38 | 1.64 | 619.68 | 1271.94 | 125.35 | 55.04 | 0.00 | 0.14 | 2.05 | 0.44 | 10.36 |
| Czechia | 2016 | 179.53 | 1.66 | 613.96 | 1258.60 | 124.19 | 55.34 | 0.00 | 0.14 | 2.05 | 0.45 | 10.63 |
| Czechia | 2017 | 179.38 | 1.72 | 604.72 | 1233.40 | 121.17 | 58.21 | 0.00 | 0.15 | 2.04 | 0.48 | 11.67 |
| Czechia | 2018 | 181.91 | 1.89 | 595.79 | 1206.90 | 117.95 | 63.96 | 0.00 | 0.15 | 2.03 | 0.54 | 13.72 |
| Czechia | 2019 | 181.79 | 1.93 | 591.00 | 1189.63 | 115.18 | 66.61 | 0.00 | 0.15 | 2.01 | 0.58 | 14.61 |
| Czechia | 2020 | 174.46 | 1.85 | 592.79 | 1179.83 | 112.57 | 61.89 | 0.00 | 0.15 | 1.99 | 0.55 | 13.54 |
| Czechia | 2021 | 173.20 | 1.84 | 591.63 | 1174.46 | 110.88 | 62.32 | 0.00 | 0.15 | 1.99 | 0.56 | 13.68 |
| Denmark | 1990 | 355.56 | 3.85 | 441.00 | 1102.64 | 171.19 | 184.37 | 0.01 | 0.32 | 2.50 | 1.08 | 45.66 |
| Denmark | 1991 | 358.45 | 3.92 | 440.30 | 1100.04 | 169.47 | 188.98 | 0.01 | 0.33 | 2.50 | 1.12 | 46.84 |
| Denmark | 1992 | 360.99 | 4.00 | 439.89 | 1097.37 | 167.70 | 193.29 | 0.01 | 0.33 | 2.49 | 1.15 | 48.08 |
| Denmark | 1993 | 366.07 | 4.15 | 439.80 | 1094.93 | 166.28 | 199.79 | 0.01 | 0.33 | 2.49 | 1.20 | 49.90 |
| Denmark | 1994 | 380.31 | 4.43 | 440.05 | 1093.21 | 165.81 | 214.50 | 0.01 | 0.35 | 2.48 | 1.29 | 53.65 |
| Denmark | 1995 | 379.56 | 4.42 | 440.68 | 1092.64 | 165.60 | 213.96 | 0.01 | 0.35 | 2.48 | 1.29 | 53.48 |
| Denmark | 1996 | 374.67 | 4.33 | 442.27 | 1090.04 | 166.00 | 208.67 | 0.01 | 0.34 | 2.46 | 1.26 | 52.19 |
| Denmark | 1997 | 371.31 | 4.25 | 444.95 | 1083.63 | 167.07 | 204.24 | 0.01 | 0.34 | 2.44 | 1.22 | 51.10 |
| Denmark | 1998 | 366.98 | 4.15 | 448.10 | 1076.17 | 167.69 | 199.28 | 0.01 | 0.34 | 2.40 | 1.19 | 49.84 |
| Denmark | 1999 | 374.55 | 4.25 | 451.05 | 1070.07 | 168.40 | 206.15 | 0.01 | 0.35 | 2.37 | 1.22 | 51.36 |
| Denmark | 2000 | 363.89 | 4.03 | 453.17 | 1068.09 | 169.01 | 194.87 | 0.01 | 0.34 | 2.36 | 1.15 | 48.47 |
| Denmark | 2001 | 356.25 | 3.92 | 454.44 | 1067.85 | 168.52 | 187.73 | 0.01 | 0.33 | 2.35 | 1.11 | 46.79 |
| Denmark | 2002 | 354.02 | 3.89 | 455.32 | 1065.84 | 168.20 | 185.82 | 0.01 | 0.33 | 2.34 | 1.10 | 46.40 |
| Denmark | 2003 | 348.16 | 3.80 | 455.96 | 1063.31 | 167.46 | 180.69 | 0.01 | 0.33 | 2.33 | 1.08 | 45.20 |
| Denmark | 2004 | 350.90 | 3.87 | 456.45 | 1061.08 | 166.66 | 184.24 | 0.01 | 0.33 | 2.32 | 1.11 | 46.16 |
| Denmark | 2005 | 355.75 | 3.96 | 456.92 | 1060.27 | 166.49 | 189.26 | 0.01 | 0.34 | 2.32 | 1.14 | 47.40 |
| Denmark | 2006 | 363.57 | 4.11 | 457.71 | 1061.92 | 167.30 | 196.27 | 0.01 | 0.34 | 2.32 | 1.17 | 49.11 |
| Denmark | 2007 | 369.93 | 4.24 | 458.80 | 1065.45 | 168.57 | 201.36 | 0.01 | 0.35 | 2.32 | 1.19 | 50.34 |
| Denmark | 2008 | 380.90 | 4.42 | 459.97 | 1070.00 | 170.32 | 210.58 | 0.01 | 0.36 | 2.33 | 1.24 | 52.35 |
| Denmark | 2009 | 386.42 | 4.52 | 460.94 | 1074.35 | 172.42 | 213.99 | 0.01 | 0.36 | 2.33 | 1.24 | 53.10 |
| Denmark | 2010 | 380.73 | 4.42 | 461.45 | 1077.49 | 173.91 | 206.82 | 0.01 | 0.35 | 2.34 | 1.19 | 51.38 |
| Denmark | 2011 | 373.39 | 4.29 | 460.03 | 1079.67 | 175.25 | 198.14 | 0.01 | 0.35 | 2.35 | 1.13 | 49.40 |
| Denmark | 2012 | 361.99 | 4.05 | 456.28 | 1081.46 | 177.87 | 184.12 | 0.01 | 0.33 | 2.37 | 1.04 | 46.21 |
| Denmark | 2013 | 360.03 | 4.01 | 451.66 | 1083.03 | 180.41 | 179.61 | 0.01 | 0.33 | 2.40 | 1.00 | 45.40 |
| Denmark | 2014 | 365.41 | 4.05 | 447.60 | 1084.29 | 183.16 | 182.25 | 0.01 | 0.34 | 2.42 | 1.00 | 46.10 |
| Denmark | 2015 | 368.36 | 4.07 | 445.53 | 1085.13 | 185.98 | 182.38 | 0.01 | 0.34 | 2.44 | 0.98 | 46.21 |
| Denmark | 2016 | 375.82 | 4.21 | 445.80 | 1083.01 | 187.46 | 188.36 | 0.01 | 0.35 | 2.43 | 1.00 | 47.78 |
| Denmark | 2017 | 378.65 | 4.28 | 447.28 | 1077.83 | 188.62 | 190.04 | 0.01 | 0.35 | 2.41 | 1.01 | 48.38 |
| Denmark | 2018 | 389.53 | 4.52 | 449.16 | 1073.79 | 190.17 | 199.36 | 0.01 | 0.36 | 2.39 | 1.05 | 50.88 |
| Denmark | 2019 | 396.47 | 4.61 | 450.64 | 1074.92 | 191.74 | 204.72 | 0.01 | 0.37 | 2.39 | 1.07 | 51.95 |
| Denmark | 2020 | 406.98 | 4.73 | 446.98 | 1083.20 | 194.00 | 212.98 | 0.01 | 0.38 | 2.42 | 1.10 | 53.63 |
| Denmark | 2021 | 392.86 | 4.46 | 449.59 | 1105.34 | 195.85 | 197.01 | 0.01 | 0.36 | 2.46 | 1.01 | 49.37 |
| Estonia | 1990 | 364.68 | 3.95 | 453.04 | 1001.12 | 180.66 | 184.02 | 0.01 | 0.36 | 2.21 | 1.02 | 47.68 |
| Estonia | 1991 | 384.85 | 4.27 | 456.88 | 1019.62 | 185.24 | 199.61 | 0.01 | 0.38 | 2.23 | 1.08 | 50.83 |
| Estonia | 1992 | 399.17 | 4.41 | 460.69 | 1038.98 | 191.26 | 207.92 | 0.01 | 0.38 | 2.26 | 1.09 | 51.93 |
| Estonia | 1993 | 428.63 | 4.86 | 464.33 | 1058.63 | 198.00 | 230.63 | 0.01 | 0.40 | 2.28 | 1.16 | 56.48 |
| Estonia | 1994 | 459.66 | 5.35 | 467.73 | 1078.66 | 205.14 | 254.51 | 0.01 | 0.43 | 2.31 | 1.24 | 61.18 |
| Estonia | 1995 | 460.38 | 5.17 | 470.76 | 1098.45 | 213.07 | 247.31 | 0.01 | 0.42 | 2.33 | 1.16 | 58.46 |
| Estonia | 1996 | 441.22 | 4.58 | 473.59 | 1119.78 | 222.35 | 218.87 | 0.01 | 0.39 | 2.36 | 0.98 | 51.19 |
| Estonia | 1997 | 458.18 | 4.70 | 476.71 | 1144.98 | 233.83 | 224.35 | 0.01 | 0.40 | 2.40 | 0.96 | 51.61 |
| Estonia | 1998 | 490.82 | 5.08 | 480.27 | 1174.01 | 247.15 | 243.67 | 0.01 | 0.42 | 2.44 | 0.99 | 54.78 |
| Estonia | 1999 | 508.90 | 5.13 | 484.46 | 1207.11 | 261.57 | 247.33 | 0.01 | 0.42 | 2.49 | 0.95 | 54.41 |
| Estonia | 2000 | 533.77 | 5.26 | 489.44 | 1244.09 | 276.06 | 257.71 | 0.01 | 0.43 | 2.54 | 0.93 | 55.05 |
| Estonia | 2001 | 578.99 | 5.70 | 495.86 | 1289.76 | 293.58 | 285.41 | 0.01 | 0.45 | 2.60 | 0.97 | 58.64 |
| Estonia | 2002 | 612.26 | 5.83 | 503.50 | 1343.89 | 314.60 | 297.66 | 0.01 | 0.46 | 2.67 | 0.95 | 58.82 |
| Estonia | 2003 | 629.18 | 5.75 | 511.00 | 1397.65 | 335.03 | 294.15 | 0.01 | 0.45 | 2.74 | 0.88 | 56.71 |
| Estonia | 2004 | 681.73 | 6.36 | 517.05 | 1442.79 | 352.12 | 329.60 | 0.01 | 0.47 | 2.79 | 0.94 | 61.35 |
| Estonia | 2005 | 709.27 | 6.67 | 520.36 | 1471.10 | 362.55 | 346.72 | 0.01 | 0.48 | 2.83 | 0.96 | 63.44 |
| Estonia | 2006 | 747.99 | 7.33 | 520.82 | 1481.89 | 365.41 | 382.57 | 0.01 | 0.50 | 2.85 | 1.05 | 69.09 |
| Estonia | 2007 | 788.70 | 8.04 | 519.81 | 1484.17 | 364.72 | 423.98 | 0.02 | 0.53 | 2.86 | 1.16 | 75.71 |
| Estonia | 2008 | 758.01 | 7.45 | 518.47 | 1483.86 | 363.27 | 394.74 | 0.01 | 0.51 | 2.86 | 1.09 | 70.78 |
| Estonia | 2009 | 756.96 | 7.38 | 517.82 | 1487.03 | 363.15 | 393.81 | 0.01 | 0.51 | 2.87 | 1.08 | 70.37 |
| Estonia | 2010 | 731.71 | 6.85 | 518.78 | 1499.49 | 366.69 | 365.01 | 0.01 | 0.49 | 2.89 | 1.00 | 65.26 |
| Estonia | 2011 | 762.42 | 7.25 | 522.67 | 1526.50 | 375.92 | 386.49 | 0.01 | 0.50 | 2.92 | 1.03 | 67.98 |
| Estonia | 2012 | 790.19 | 7.51 | 529.02 | 1565.55 | 389.28 | 400.90 | 0.01 | 0.50 | 2.96 | 1.03 | 69.06 |
| Estonia | 2013 | 746.17 | 6.48 | 536.28 | 1610.73 | 404.93 | 341.23 | 0.01 | 0.46 | 3.00 | 0.84 | 58.78 |
| Estonia | 2014 | 739.10 | 6.15 | 542.81 | 1655.87 | 421.15 | 317.95 | 0.01 | 0.45 | 3.05 | 0.75 | 54.60 |
| Estonia | 2015 | 724.25 | 5.65 | 547.05 | 1694.85 | 435.19 | 289.06 | 0.01 | 0.43 | 3.10 | 0.66 | 49.75 |
| Estonia | 2016 | 728.01 | 5.52 | 548.81 | 1730.31 | 450.44 | 277.58 | 0.01 | 0.42 | 3.15 | 0.62 | 47.93 |
| Estonia | 2017 | 707.31 | 4.88 | 549.58 | 1766.40 | 467.13 | 240.17 | 0.01 | 0.40 | 3.21 | 0.51 | 42.40 |
| Estonia | 2018 | 694.74 | 4.45 | 550.38 | 1799.57 | 481.89 | 212.85 | 0.01 | 0.39 | 3.27 | 0.44 | 38.58 |
| Estonia | 2019 | 687.46 | 4.11 | 552.30 | 1826.37 | 493.89 | 193.57 | 0.01 | 0.38 | 3.31 | 0.39 | 35.75 |
| Estonia | 2020 | 710.67 | 4.47 | 558.38 | 1848.70 | 500.77 | 209.90 | 0.01 | 0.38 | 3.31 | 0.42 | 37.95 |
| Estonia | 2021 | 753.64 | 4.98 | 566.83 | 1877.49 | 513.04 | 240.60 | 0.01 | 0.40 | 3.31 | 0.47 | 41.54 |
| Finland | 1990 | 285.05 | 3.01 | 419.46 | 906.20 | 144.01 | 141.04 | 0.01 | 0.31 | 2.16 | 0.98 | 40.32 |
| Finland | 1991 | 295.21 | 3.15 | 423.88 | 906.90 | 146.41 | 148.80 | 0.01 | 0.33 | 2.14 | 1.02 | 42.22 |
| Finland | 1992 | 297.66 | 3.17 | 428.62 | 909.29 | 149.19 | 148.48 | 0.01 | 0.33 | 2.12 | 1.00 | 41.93 |
| Finland | 1993 | 300.38 | 3.16 | 433.60 | 913.21 | 151.98 | 148.40 | 0.01 | 0.33 | 2.11 | 0.98 | 41.55 |
| Finland | 1994 | 304.93 | 3.20 | 438.65 | 918.34 | 154.65 | 150.28 | 0.01 | 0.33 | 2.09 | 0.97 | 41.67 |
| Finland | 1995 | 315.72 | 3.36 | 443.71 | 924.72 | 156.94 | 158.78 | 0.01 | 0.34 | 2.08 | 1.01 | 43.56 |
| Finland | 1996 | 324.62 | 3.49 | 449.85 | 933.60 | 158.83 | 165.79 | 0.01 | 0.35 | 2.08 | 1.04 | 44.89 |
| Finland | 1997 | 327.67 | 3.51 | 457.39 | 945.39 | 160.46 | 167.21 | 0.01 | 0.35 | 2.07 | 1.04 | 44.60 |
| Finland | 1998 | 329.36 | 3.46 | 465.10 | 958.53 | 162.79 | 166.56 | 0.01 | 0.34 | 2.06 | 1.02 | 43.62 |
| Finland | 1999 | 340.08 | 3.58 | 471.75 | 971.34 | 164.82 | 175.26 | 0.01 | 0.35 | 2.06 | 1.06 | 44.98 |
| Finland | 2000 | 350.43 | 3.66 | 476.08 | 982.37 | 167.58 | 182.85 | 0.01 | 0.36 | 2.06 | 1.09 | 46.11 |
| Finland | 2001 | 352.94 | 3.64 | 477.90 | 995.10 | 171.44 | 181.50 | 0.01 | 0.35 | 2.08 | 1.06 | 45.29 |
| Finland | 2002 | 357.25 | 3.63 | 478.22 | 1012.24 | 176.79 | 180.47 | 0.01 | 0.35 | 2.12 | 1.02 | 44.51 |
| Finland | 2003 | 367.56 | 3.71 | 477.62 | 1031.03 | 183.32 | 184.24 | 0.01 | 0.36 | 2.16 | 1.01 | 44.89 |
| Finland | 2004 | 385.87 | 3.96 | 476.73 | 1048.75 | 189.74 | 196.13 | 0.01 | 0.37 | 2.20 | 1.03 | 47.27 |
| Finland | 2005 | 399.01 | 4.14 | 476.10 | 1062.60 | 194.78 | 204.23 | 0.01 | 0.38 | 2.23 | 1.05 | 48.84 |
| Finland | 2006 | 417.96 | 4.39 | 475.42 | 1073.49 | 198.88 | 219.09 | 0.01 | 0.39 | 2.26 | 1.10 | 51.75 |
| Finland | 2007 | 445.61 | 4.81 | 474.10 | 1083.89 | 202.96 | 242.65 | 0.01 | 0.41 | 2.29 | 1.20 | 56.68 |
| Finland | 2008 | 459.85 | 5.02 | 472.39 | 1093.61 | 206.75 | 253.10 | 0.01 | 0.42 | 2.32 | 1.22 | 58.78 |
| Finland | 2009 | 471.10 | 5.14 | 470.51 | 1102.51 | 210.48 | 260.62 | 0.01 | 0.43 | 2.34 | 1.24 | 60.13 |
| Finland | 2010 | 476.33 | 5.16 | 468.67 | 1110.28 | 214.17 | 262.16 | 0.01 | 0.43 | 2.37 | 1.22 | 60.19 |
| Finland | 2011 | 475.28 | 5.07 | 466.46 | 1117.03 | 218.19 | 257.09 | 0.01 | 0.43 | 2.39 | 1.18 | 58.91 |
| Finland | 2012 | 474.63 | 4.98 | 463.58 | 1122.97 | 222.74 | 251.89 | 0.01 | 0.42 | 2.42 | 1.13 | 57.71 |
| Finland | 2013 | 472.26 | 4.87 | 460.56 | 1128.43 | 227.47 | 244.78 | 0.01 | 0.42 | 2.45 | 1.08 | 56.21 |
| Finland | 2014 | 465.84 | 4.69 | 457.90 | 1133.63 | 231.18 | 234.66 | 0.01 | 0.41 | 2.48 | 1.02 | 54.01 |
| Finland | 2015 | 461.64 | 4.57 | 456.08 | 1138.75 | 234.17 | 227.47 | 0.01 | 0.41 | 2.50 | 0.97 | 52.46 |
| Finland | 2016 | 474.91 | 4.74 | 456.23 | 1149.06 | 237.50 | 237.41 | 0.01 | 0.41 | 2.52 | 1.00 | 54.27 |
| Finland | 2017 | 491.42 | 4.99 | 458.21 | 1166.01 | 240.80 | 250.63 | 0.01 | 0.42 | 2.54 | 1.04 | 56.54 |
| Finland | 2018 | 509.96 | 5.28 | 460.56 | 1184.39 | 244.71 | 265.25 | 0.01 | 0.43 | 2.57 | 1.08 | 59.03 |
| Finland | 2019 | 531.61 | 5.58 | 461.88 | 1198.91 | 248.40 | 283.21 | 0.01 | 0.44 | 2.60 | 1.14 | 62.10 |
| Finland | 2020 | 546.01 | 5.76 | 455.24 | 1210.87 | 252.42 | 293.59 | 0.01 | 0.45 | 2.66 | 1.16 | 64.19 |
| Finland | 2021 | 525.35 | 5.34 | 458.98 | 1221.76 | 256.47 | 268.88 | 0.01 | 0.43 | 2.66 | 1.05 | 58.81 |
| France | 1990 | 270.51 | 5.88 | 539.15 | 1017.76 | 101.53 | 168.98 | 0.01 | 0.27 | 1.89 | 1.66 | 55.70 |
| France | 1991 | 274.98 | 5.92 | 536.65 | 1021.71 | 101.96 | 173.02 | 0.01 | 0.27 | 1.90 | 1.70 | 56.70 |
| France | 1992 | 276.99 | 5.98 | 533.81 | 1024.99 | 102.52 | 174.47 | 0.01 | 0.27 | 1.92 | 1.70 | 57.21 |
| France | 1993 | 276.57 | 5.97 | 530.68 | 1027.49 | 102.93 | 173.64 | 0.01 | 0.27 | 1.94 | 1.69 | 57.03 |
| France | 1994 | 271.97 | 5.83 | 527.30 | 1029.09 | 103.44 | 168.53 | 0.01 | 0.26 | 1.95 | 1.63 | 55.36 |
| France | 1995 | 269.67 | 5.84 | 523.71 | 1029.71 | 103.88 | 165.78 | 0.01 | 0.26 | 1.97 | 1.60 | 54.87 |
| France | 1996 | 266.81 | 5.79 | 519.50 | 1028.81 | 104.31 | 162.50 | 0.01 | 0.26 | 1.98 | 1.56 | 54.11 |
| France | 1997 | 262.46 | 5.72 | 514.48 | 1026.82 | 105.03 | 157.43 | 0.01 | 0.26 | 2.00 | 1.50 | 52.86 |
| France | 1998 | 259.78 | 5.60 | 508.98 | 1024.99 | 105.98 | 153.80 | 0.01 | 0.25 | 2.01 | 1.45 | 51.73 |
| France | 1999 | 246.59 | 4.95 | 503.36 | 1024.62 | 107.11 | 139.49 | 0.01 | 0.24 | 2.04 | 1.30 | 46.00 |
| France | 2000 | 219.45 | 3.54 | 497.94 | 1026.97 | 108.87 | 110.59 | 0.01 | 0.21 | 2.06 | 1.02 | 33.83 |
| France | 2001 | 219.27 | 3.36 | 492.28 | 1034.46 | 112.11 | 107.15 | 0.01 | 0.21 | 2.10 | 0.96 | 32.20 |
| France | 2002 | 226.29 | 3.50 | 486.14 | 1047.12 | 117.35 | 108.94 | 0.01 | 0.22 | 2.15 | 0.93 | 32.81 |
| France | 2003 | 235.21 | 3.60 | 480.20 | 1062.69 | 124.15 | 111.06 | 0.01 | 0.22 | 2.21 | 0.89 | 33.26 |
| France | 2004 | 243.31 | 3.66 | 475.10 | 1078.88 | 130.58 | 112.72 | 0.01 | 0.23 | 2.27 | 0.86 | 33.50 |
| France | 2005 | 253.26 | 3.73 | 471.49 | 1093.43 | 137.11 | 116.15 | 0.01 | 0.23 | 2.32 | 0.85 | 34.09 |
| France | 2006 | 262.21 | 3.76 | 469.33 | 1109.52 | 143.98 | 118.23 | 0.01 | 0.24 | 2.36 | 0.82 | 34.24 |
| France | 2007 | 271.27 | 3.77 | 467.90 | 1129.28 | 152.05 | 119.22 | 0.01 | 0.24 | 2.41 | 0.78 | 34.03 |
| France | 2008 | 279.20 | 3.72 | 466.87 | 1148.86 | 159.88 | 119.32 | 0.01 | 0.24 | 2.46 | 0.75 | 33.41 |
| France | 2009 | 283.82 | 3.59 | 465.94 | 1164.38 | 166.00 | 117.82 | 0.01 | 0.24 | 2.50 | 0.71 | 32.33 |
| France | 2010 | 288.19 | 3.61 | 464.77 | 1171.98 | 168.92 | 119.28 | 0.01 | 0.25 | 2.52 | 0.71 | 32.57 |
| France | 2011 | 282.95 | 3.55 | 463.19 | 1171.41 | 166.35 | 116.60 | 0.01 | 0.24 | 2.53 | 0.70 | 31.93 |
| France | 2012 | 269.95 | 3.35 | 461.40 | 1166.32 | 159.56 | 110.39 | 0.01 | 0.23 | 2.53 | 0.69 | 30.25 |
| France | 2013 | 259.45 | 3.25 | 459.70 | 1158.71 | 151.14 | 108.31 | 0.01 | 0.22 | 2.52 | 0.72 | 29.72 |
| France | 2014 | 251.81 | 3.24 | 458.39 | 1150.58 | 143.08 | 108.74 | 0.01 | 0.22 | 2.51 | 0.76 | 30.05 |
| France | 2015 | 250.81 | 3.35 | 457.75 | 1143.88 | 137.86 | 112.94 | 0.01 | 0.22 | 2.50 | 0.82 | 31.54 |
| France | 2016 | 251.84 | 3.53 | 456.59 | 1132.61 | 135.04 | 116.79 | 0.01 | 0.22 | 2.48 | 0.86 | 33.32 |
| France | 2017 | 250.70 | 3.55 | 454.28 | 1114.44 | 132.41 | 118.29 | 0.01 | 0.22 | 2.45 | 0.89 | 34.13 |
| France | 2018 | 248.83 | 3.53 | 451.99 | 1096.86 | 130.20 | 118.63 | 0.01 | 0.23 | 2.43 | 0.91 | 34.54 |
| France | 2019 | 248.18 | 3.53 | 450.90 | 1087.33 | 128.62 | 119.56 | 0.01 | 0.23 | 2.41 | 0.93 | 34.97 |
| France | 2020 | 243.80 | 3.48 | 448.33 | 1079.60 | 126.23 | 117.57 | 0.01 | 0.23 | 2.41 | 0.93 | 34.67 |
| France | 2021 | 236.93 | 3.37 | 448.16 | 1078.19 | 122.40 | 114.54 | 0.01 | 0.22 | 2.41 | 0.94 | 33.81 |
| Germany | 1990 | 220.09 | 2.19 | 378.95 | 855.42 | 121.62 | 98.46 | 0.01 | 0.26 | 2.26 | 0.81 | 30.76 |
| Germany | 1991 | 237.67 | 2.44 | 381.19 | 864.04 | 123.73 | 113.94 | 0.01 | 0.28 | 2.27 | 0.92 | 35.05 |
| Germany | 1992 | 245.62 | 2.52 | 383.61 | 872.05 | 125.56 | 120.06 | 0.01 | 0.28 | 2.27 | 0.96 | 36.39 |
| Germany | 1993 | 246.45 | 2.49 | 386.07 | 879.34 | 127.15 | 119.30 | 0.01 | 0.28 | 2.28 | 0.94 | 35.76 |
| Germany | 1994 | 246.29 | 2.44 | 388.44 | 885.90 | 128.30 | 117.99 | 0.01 | 0.28 | 2.28 | 0.92 | 34.93 |
| Germany | 1995 | 244.71 | 2.33 | 390.61 | 891.79 | 129.11 | 115.60 | 0.01 | 0.27 | 2.28 | 0.90 | 33.67 |
| Germany | 1996 | 245.61 | 2.35 | 393.33 | 897.03 | 129.64 | 115.96 | 0.01 | 0.27 | 2.28 | 0.89 | 33.61 |
| Germany | 1997 | 243.22 | 2.32 | 397.04 | 901.71 | 129.63 | 113.58 | 0.01 | 0.27 | 2.27 | 0.88 | 32.77 |
| Germany | 1998 | 242.89 | 2.30 | 401.09 | 905.92 | 129.73 | 113.16 | 0.01 | 0.27 | 2.26 | 0.87 | 32.36 |
| Germany | 1999 | 247.68 | 2.38 | 404.88 | 909.81 | 130.11 | 117.57 | 0.01 | 0.27 | 2.25 | 0.90 | 33.45 |
| Germany | 2000 | 250.65 | 2.46 | 407.80 | 913.50 | 130.65 | 120.01 | 0.01 | 0.27 | 2.24 | 0.92 | 34.14 |
| Germany | 2001 | 246.71 | 2.40 | 410.48 | 916.95 | 131.53 | 115.19 | 0.01 | 0.27 | 2.23 | 0.88 | 32.71 |
| Germany | 2002 | 244.69 | 2.37 | 413.70 | 920.01 | 133.31 | 111.39 | 0.01 | 0.27 | 2.22 | 0.84 | 31.63 |
| Germany | 2003 | 244.79 | 2.38 | 417.07 | 922.73 | 135.01 | 109.78 | 0.01 | 0.27 | 2.21 | 0.81 | 31.21 |
| Germany | 2004 | 244.00 | 2.37 | 420.20 | 925.26 | 136.74 | 107.26 | 0.01 | 0.26 | 2.20 | 0.78 | 30.52 |
| Germany | 2005 | 243.67 | 2.35 | 422.70 | 927.76 | 138.23 | 105.44 | 0.01 | 0.26 | 2.19 | 0.76 | 29.94 |
| Germany | 2006 | 243.45 | 2.34 | 424.67 | 930.51 | 139.33 | 104.12 | 0.01 | 0.26 | 2.19 | 0.75 | 29.52 |
| Germany | 2007 | 245.59 | 2.38 | 426.43 | 933.67 | 139.87 | 105.71 | 0.01 | 0.26 | 2.19 | 0.76 | 29.90 |
| Germany | 2008 | 249.76 | 2.51 | 427.96 | 937.24 | 140.74 | 109.02 | 0.01 | 0.27 | 2.19 | 0.77 | 31.04 |
| Germany | 2009 | 252.80 | 2.64 | 429.22 | 941.16 | 141.49 | 111.31 | 0.01 | 0.27 | 2.19 | 0.79 | 31.99 |
| Germany | 2010 | 253.32 | 2.72 | 430.14 | 945.40 | 142.09 | 111.23 | 0.01 | 0.27 | 2.20 | 0.78 | 32.25 |
| Germany | 2011 | 249.12 | 2.61 | 430.89 | 951.47 | 142.12 | 107.00 | 0.01 | 0.26 | 2.21 | 0.75 | 30.80 |
| Germany | 2012 | 246.49 | 2.59 | 431.64 | 959.98 | 141.57 | 104.92 | 0.01 | 0.26 | 2.22 | 0.74 | 30.13 |
| Germany | 2013 | 248.93 | 2.71 | 432.43 | 969.46 | 140.89 | 108.04 | 0.01 | 0.26 | 2.24 | 0.77 | 31.10 |
| Germany | 2014 | 250.39 | 2.81 | 433.30 | 978.36 | 139.79 | 110.60 | 0.01 | 0.26 | 2.26 | 0.79 | 31.90 |
| Germany | 2015 | 257.02 | 2.99 | 434.25 | 984.79 | 139.72 | 117.30 | 0.01 | 0.26 | 2.27 | 0.84 | 33.82 |
| Germany | 2016 | 261.51 | 3.12 | 435.34 | 987.50 | 140.32 | 121.19 | 0.01 | 0.26 | 2.27 | 0.86 | 35.08 |
| Germany | 2017 | 262.70 | 3.20 | 436.59 | 987.88 | 141.13 | 121.56 | 0.01 | 0.27 | 2.26 | 0.86 | 35.46 |
| Germany | 2018 | 266.33 | 3.27 | 437.86 | 987.53 | 141.93 | 124.41 | 0.01 | 0.27 | 2.26 | 0.88 | 36.26 |
| Germany | 2019 | 267.42 | 3.24 | 439.06 | 988.34 | 142.77 | 124.66 | 0.01 | 0.27 | 2.25 | 0.87 | 36.10 |
| Germany | 2020 | 265.55 | 3.19 | 438.95 | 990.98 | 143.33 | 122.21 | 0.01 | 0.27 | 2.26 | 0.85 | 35.34 |
| Germany | 2021 | 267.54 | 3.16 | 440.21 | 1026.07 | 145.55 | 121.99 | 0.01 | 0.26 | 2.33 | 0.84 | 34.43 |
| Greece | 1990 | 181.55 | 2.27 | 369.42 | 571.16 | 102.35 | 79.20 | 0.01 | 0.32 | 1.55 | 0.77 | 35.29 |
| Greece | 1991 | 191.92 | 2.07 | 367.83 | 589.33 | 111.21 | 80.71 | 0.01 | 0.33 | 1.60 | 0.73 | 33.95 |
| Greece | 1992 | 208.17 | 2.17 | 366.22 | 606.73 | 119.64 | 88.53 | 0.01 | 0.34 | 1.66 | 0.74 | 36.00 |
| Greece | 1993 | 228.40 | 2.51 | 364.59 | 622.71 | 127.12 | 101.28 | 0.01 | 0.37 | 1.71 | 0.80 | 40.54 |
| Greece | 1994 | 247.77 | 2.69 | 362.94 | 636.86 | 133.90 | 113.87 | 0.01 | 0.39 | 1.75 | 0.85 | 44.08 |
| Greece | 1995 | 265.68 | 2.84 | 361.30 | 648.86 | 139.29 | 126.39 | 0.01 | 0.41 | 1.80 | 0.91 | 47.32 |
| Greece | 1996 | 280.72 | 2.97 | 359.42 | 659.66 | 144.51 | 136.20 | 0.01 | 0.43 | 1.84 | 0.94 | 49.80 |
| Greece | 1997 | 293.25 | 3.08 | 357.28 | 670.19 | 149.27 | 143.98 | 0.01 | 0.44 | 1.88 | 0.96 | 51.72 |
| Greece | 1998 | 305.20 | 3.20 | 355.14 | 679.99 | 153.79 | 151.41 | 0.01 | 0.45 | 1.91 | 0.98 | 53.65 |
| Greece | 1999 | 315.24 | 3.27 | 353.25 | 688.16 | 157.47 | 157.77 | 0.01 | 0.46 | 1.95 | 1.00 | 55.10 |
| Greece | 2000 | 324.08 | 3.36 | 351.88 | 694.34 | 159.78 | 164.29 | 0.01 | 0.47 | 1.97 | 1.03 | 56.68 |
| Greece | 2001 | 332.42 | 3.49 | 350.91 | 699.49 | 162.07 | 170.35 | 0.01 | 0.48 | 1.99 | 1.05 | 58.47 |
| Greece | 2002 | 329.48 | 3.44 | 350.08 | 704.54 | 164.02 | 165.47 | 0.01 | 0.47 | 2.01 | 1.01 | 57.01 |
| Greece | 2003 | 333.08 | 3.50 | 349.55 | 709.38 | 165.99 | 167.09 | 0.01 | 0.47 | 2.03 | 1.01 | 57.51 |
| Greece | 2004 | 345.50 | 3.67 | 349.51 | 713.88 | 168.06 | 177.44 | 0.01 | 0.48 | 2.04 | 1.06 | 60.36 |
| Greece | 2005 | 355.77 | 3.81 | 350.12 | 717.83 | 169.13 | 186.63 | 0.01 | 0.50 | 2.05 | 1.10 | 62.71 |
| Greece | 2006 | 355.25 | 3.79 | 351.75 | 720.76 | 169.94 | 185.31 | 0.01 | 0.49 | 2.05 | 1.09 | 62.07 |
| Greece | 2007 | 359.65 | 4.01 | 354.27 | 722.36 | 170.27 | 189.37 | 0.01 | 0.50 | 2.04 | 1.11 | 63.91 |
| Greece | 2008 | 350.63 | 3.80 | 357.01 | 722.55 | 169.24 | 181.39 | 0.01 | 0.49 | 2.02 | 1.07 | 60.98 |
| Greece | 2009 | 350.54 | 3.97 | 359.36 | 721.26 | 168.39 | 182.15 | 0.01 | 0.49 | 2.01 | 1.08 | 62.06 |
| Greece | 2010 | 337.23 | 3.86 | 360.69 | 718.30 | 166.68 | 170.55 | 0.01 | 0.47 | 1.99 | 1.02 | 59.29 |
| Greece | 2011 | 321.84 | 3.66 | 360.52 | 712.16 | 164.49 | 157.34 | 0.01 | 0.45 | 1.98 | 0.96 | 55.87 |
| Greece | 2012 | 312.73 | 3.60 | 359.22 | 702.63 | 162.44 | 150.29 | 0.01 | 0.45 | 1.96 | 0.93 | 54.59 |
| Greece | 2013 | 304.08 | 3.59 | 357.55 | 691.66 | 159.89 | 144.19 | 0.01 | 0.44 | 1.93 | 0.90 | 53.93 |
| Greece | 2014 | 303.86 | 3.71 | 356.22 | 681.29 | 157.83 | 146.03 | 0.01 | 0.45 | 1.91 | 0.93 | 55.56 |
| Greece | 2015 | 307.45 | 3.88 | 355.89 | 673.27 | 156.09 | 151.36 | 0.01 | 0.46 | 1.89 | 0.97 | 57.99 |
| Greece | 2016 | 308.04 | 3.96 | 357.11 | 665.84 | 154.38 | 153.66 | 0.01 | 0.46 | 1.86 | 1.00 | 59.21 |
| Greece | 2017 | 310.67 | 4.13 | 359.47 | 657.18 | 152.31 | 158.36 | 0.01 | 0.47 | 1.83 | 1.04 | 61.50 |
| Greece | 2018 | 316.13 | 4.38 | 362.28 | 648.97 | 150.01 | 166.12 | 0.01 | 0.49 | 1.79 | 1.11 | 64.80 |
| Greece | 2019 | 316.79 | 4.42 | 364.89 | 643.16 | 148.15 | 168.64 | 0.01 | 0.49 | 1.76 | 1.14 | 65.74 |
| Greece | 2020 | 309.14 | 4.32 | 377.56 | 708.77 | 146.10 | 163.04 | 0.01 | 0.44 | 1.88 | 1.12 | 60.02 |
| Greece | 2021 | 306.07 | 4.31 | 376.42 | 699.88 | 142.12 | 163.95 | 0.01 | 0.44 | 1.86 | 1.15 | 60.73 |
| Hungary | 1990 | 121.55 | 1.22 | 422.69 | 675.22 | 78.29 | 43.26 | 0.00 | 0.18 | 1.60 | 0.55 | 15.35 |
| Hungary | 1991 | 121.44 | 1.19 | 420.64 | 673.99 | 78.62 | 42.83 | 0.00 | 0.18 | 1.60 | 0.54 | 15.14 |
| Hungary | 1992 | 120.65 | 1.17 | 418.61 | 671.96 | 78.90 | 41.75 | 0.00 | 0.18 | 1.61 | 0.53 | 14.77 |
| Hungary | 1993 | 121.74 | 1.19 | 416.73 | 669.65 | 79.13 | 42.61 | 0.00 | 0.18 | 1.61 | 0.54 | 15.21 |
| Hungary | 1994 | 123.82 | 1.25 | 415.11 | 667.58 | 79.35 | 44.46 | 0.00 | 0.19 | 1.61 | 0.56 | 16.15 |
| Hungary | 1995 | 121.05 | 1.17 | 413.88 | 666.25 | 79.49 | 41.56 | 0.00 | 0.18 | 1.61 | 0.52 | 14.88 |
| Hungary | 1996 | 118.92 | 1.10 | 412.36 | 661.58 | 79.38 | 39.55 | 0.00 | 0.18 | 1.60 | 0.50 | 14.03 |
| Hungary | 1997 | 117.30 | 1.07 | 410.20 | 651.64 | 78.58 | 38.72 | 0.00 | 0.18 | 1.59 | 0.49 | 13.86 |
| Hungary | 1998 | 117.80 | 1.09 | 407.97 | 640.20 | 77.73 | 40.07 | 0.00 | 0.18 | 1.57 | 0.52 | 14.60 |
| Hungary | 1999 | 119.06 | 1.13 | 406.26 | 631.02 | 77.10 | 41.97 | 0.00 | 0.19 | 1.55 | 0.54 | 15.64 |
| Hungary | 2000 | 117.49 | 1.08 | 405.64 | 627.91 | 76.96 | 40.53 | 0.00 | 0.19 | 1.55 | 0.53 | 14.99 |
| Hungary | 2001 | 119.07 | 1.11 | 406.56 | 634.00 | 77.95 | 41.12 | 0.00 | 0.19 | 1.56 | 0.53 | 15.22 |
| Hungary | 2002 | 121.16 | 1.13 | 408.71 | 647.27 | 80.07 | 41.08 | 0.00 | 0.19 | 1.58 | 0.51 | 14.98 |
| Hungary | 2003 | 124.40 | 1.15 | 411.45 | 663.82 | 82.58 | 41.82 | 0.00 | 0.19 | 1.61 | 0.51 | 14.99 |
| Hungary | 2004 | 127.64 | 1.20 | 414.18 | 679.73 | 84.89 | 42.75 | 0.00 | 0.19 | 1.64 | 0.50 | 15.16 |
| Hungary | 2005 | 129.91 | 1.21 | 416.28 | 691.09 | 86.56 | 43.36 | 0.00 | 0.19 | 1.66 | 0.50 | 15.13 |
| Hungary | 2006 | 130.82 | 1.20 | 418.45 | 699.08 | 87.82 | 43.00 | 0.00 | 0.19 | 1.67 | 0.49 | 14.81 |
| Hungary | 2007 | 132.02 | 1.18 | 421.36 | 706.92 | 89.23 | 42.79 | 0.00 | 0.19 | 1.68 | 0.48 | 14.49 |
| Hungary | 2008 | 132.49 | 1.17 | 424.36 | 713.75 | 90.40 | 42.09 | 0.00 | 0.19 | 1.68 | 0.47 | 14.12 |
| Hungary | 2009 | 133.76 | 1.20 | 426.79 | 718.72 | 91.18 | 42.58 | 0.00 | 0.19 | 1.68 | 0.47 | 14.30 |
| Hungary | 2010 | 132.68 | 1.19 | 427.99 | 720.97 | 91.55 | 41.13 | 0.00 | 0.18 | 1.68 | 0.45 | 13.81 |
| Hungary | 2011 | 129.61 | 1.11 | 427.48 | 718.71 | 91.45 | 38.16 | 0.00 | 0.18 | 1.68 | 0.42 | 12.67 |
| Hungary | 2012 | 130.91 | 1.17 | 425.72 | 712.52 | 90.75 | 40.16 | 0.00 | 0.18 | 1.67 | 0.44 | 13.58 |
| Hungary | 2013 | 127.82 | 1.11 | 423.50 | 705.03 | 89.79 | 38.04 | 0.00 | 0.18 | 1.66 | 0.42 | 12.86 |
| Hungary | 2014 | 125.80 | 1.06 | 421.59 | 698.88 | 88.89 | 36.91 | 0.00 | 0.18 | 1.66 | 0.42 | 12.44 |
| Hungary | 2015 | 127.30 | 1.11 | 420.79 | 696.70 | 88.66 | 38.64 | 0.00 | 0.18 | 1.66 | 0.44 | 13.18 |
| Hungary | 2016 | 126.99 | 1.09 | 420.21 | 694.22 | 89.21 | 37.78 | 0.00 | 0.18 | 1.65 | 0.42 | 12.91 |
| Hungary | 2017 | 130.40 | 1.16 | 418.97 | 687.99 | 89.82 | 40.58 | 0.00 | 0.19 | 1.64 | 0.45 | 14.18 |
| Hungary | 2018 | 133.13 | 1.23 | 417.88 | 681.87 | 90.24 | 42.88 | 0.00 | 0.20 | 1.63 | 0.48 | 15.33 |
| Hungary | 2019 | 134.84 | 1.26 | 417.76 | 679.72 | 90.47 | 44.36 | 0.00 | 0.20 | 1.63 | 0.49 | 15.93 |
| Hungary | 2020 | 133.91 | 1.24 | 420.09 | 680.36 | 90.53 | 43.38 | 0.00 | 0.20 | 1.62 | 0.48 | 15.52 |
| Hungary | 2021 | 134.44 | 1.23 | 420.93 | 678.80 | 90.28 | 44.16 | 0.00 | 0.20 | 1.61 | 0.49 | 15.71 |
| Iceland | 1990 | 284.74 | 3.24 | 411.75 | 911.04 | 146.24 | 138.50 | 0.01 | 0.31 | 2.21 | 0.95 | 41.18 |
| Iceland | 1991 | 287.20 | 3.25 | 412.05 | 922.07 | 148.68 | 138.52 | 0.01 | 0.31 | 2.24 | 0.93 | 40.90 |
| Iceland | 1992 | 287.84 | 3.23 | 412.53 | 932.26 | 151.48 | 136.37 | 0.01 | 0.31 | 2.26 | 0.90 | 40.09 |
| Iceland | 1993 | 288.60 | 3.21 | 413.00 | 940.98 | 153.61 | 134.99 | 0.01 | 0.31 | 2.28 | 0.88 | 39.46 |
| Iceland | 1994 | 293.84 | 3.25 | 413.42 | 948.73 | 155.97 | 137.87 | 0.01 | 0.31 | 2.29 | 0.88 | 39.97 |
| Iceland | 1995 | 298.03 | 3.31 | 413.81 | 955.45 | 158.07 | 139.95 | 0.01 | 0.31 | 2.31 | 0.89 | 40.42 |
| Iceland | 1996 | 304.23 | 3.43 | 414.29 | 959.48 | 159.37 | 144.87 | 0.01 | 0.32 | 2.32 | 0.91 | 41.75 |
| Iceland | 1997 | 312.08 | 3.55 | 415.23 | 961.48 | 160.97 | 151.11 | 0.01 | 0.32 | 2.32 | 0.94 | 43.30 |
| Iceland | 1998 | 317.82 | 3.61 | 416.53 | 962.64 | 162.51 | 155.31 | 0.01 | 0.33 | 2.31 | 0.96 | 44.20 |
| Iceland | 1999 | 326.03 | 3.65 | 417.95 | 964.19 | 164.39 | 161.64 | 0.01 | 0.34 | 2.31 | 0.98 | 45.34 |
| Iceland | 2000 | 330.34 | 3.57 | 419.53 | 968.36 | 166.15 | 164.19 | 0.01 | 0.34 | 2.31 | 0.99 | 45.11 |
| Iceland | 2001 | 331.82 | 3.54 | 421.59 | 974.34 | 167.86 | 163.95 | 0.01 | 0.34 | 2.31 | 0.98 | 44.66 |
| Iceland | 2002 | 329.96 | 3.47 | 424.39 | 981.45 | 170.10 | 159.87 | 0.01 | 0.34 | 2.31 | 0.94 | 43.33 |
| Iceland | 2003 | 327.39 | 3.40 | 427.43 | 989.37 | 172.11 | 155.28 | 0.01 | 0.33 | 2.31 | 0.90 | 41.88 |
| Iceland | 2004 | 329.47 | 3.42 | 430.03 | 996.92 | 174.07 | 155.40 | 0.01 | 0.33 | 2.32 | 0.89 | 41.71 |
| Iceland | 2005 | 331.02 | 3.42 | 431.63 | 1004.10 | 176.21 | 154.81 | 0.01 | 0.33 | 2.33 | 0.88 | 41.37 |
| Iceland | 2006 | 338.44 | 3.52 | 432.27 | 1010.36 | 177.90 | 160.54 | 0.01 | 0.33 | 2.34 | 0.90 | 42.57 |
| Iceland | 2007 | 345.37 | 3.64 | 432.49 | 1016.03 | 179.94 | 165.43 | 0.01 | 0.34 | 2.35 | 0.92 | 43.75 |
| Iceland | 2008 | 353.71 | 3.80 | 432.67 | 1021.40 | 181.84 | 171.87 | 0.01 | 0.35 | 2.36 | 0.95 | 45.37 |
| Iceland | 2009 | 366.75 | 4.02 | 433.06 | 1026.62 | 183.95 | 182.80 | 0.01 | 0.36 | 2.37 | 0.99 | 47.98 |
| Iceland | 2010 | 375.47 | 4.17 | 433.99 | 1033.46 | 186.71 | 188.76 | 0.01 | 0.36 | 2.38 | 1.01 | 49.36 |
| Iceland | 2011 | 382.03 | 4.20 | 435.87 | 1048.48 | 192.19 | 189.85 | 0.01 | 0.36 | 2.41 | 0.99 | 49.12 |
| Iceland | 2012 | 393.91 | 4.28 | 438.60 | 1073.18 | 200.07 | 193.84 | 0.01 | 0.37 | 2.45 | 0.97 | 49.28 |
| Iceland | 2013 | 408.93 | 4.40 | 441.70 | 1102.48 | 209.30 | 199.62 | 0.01 | 0.37 | 2.50 | 0.95 | 49.70 |
| Iceland | 2014 | 423.15 | 4.50 | 444.59 | 1130.94 | 218.28 | 204.86 | 0.01 | 0.37 | 2.54 | 0.94 | 50.05 |
| Iceland | 2015 | 439.97 | 4.69 | 446.73 | 1153.21 | 225.31 | 214.66 | 0.01 | 0.38 | 2.58 | 0.95 | 51.55 |
| Iceland | 2016 | 462.04 | 5.02 | 448.45 | 1170.49 | 230.81 | 231.22 | 0.01 | 0.39 | 2.61 | 1.00 | 54.72 |
| Iceland | 2017 | 484.15 | 5.38 | 450.14 | 1185.67 | 236.22 | 247.93 | 0.01 | 0.41 | 2.63 | 1.05 | 58.02 |
| Iceland | 2018 | 508.07 | 5.92 | 451.56 | 1197.09 | 240.62 | 267.44 | 0.01 | 0.42 | 2.65 | 1.11 | 62.54 |
| Iceland | 2019 | 520.15 | 6.16 | 452.39 | 1203.62 | 243.15 | 277.01 | 0.01 | 0.43 | 2.66 | 1.14 | 64.62 |
| Iceland | 2020 | 513.54 | 6.04 | 448.96 | 1202.80 | 243.06 | 270.48 | 0.01 | 0.43 | 2.68 | 1.11 | 63.51 |
| Iceland | 2021 | 538.10 | 6.46 | 448.76 | 1201.04 | 242.34 | 295.76 | 0.01 | 0.45 | 2.68 | 1.22 | 68.62 |
| Ireland | 1990 | 205.72 | 2.18 | 422.87 | 1129.06 | 123.66 | 82.06 | 0.01 | 0.18 | 2.67 | 0.66 | 21.90 |
| Ireland | 1991 | 222.96 | 2.33 | 425.97 | 1138.79 | 131.54 | 91.42 | 0.01 | 0.20 | 2.67 | 0.69 | 23.97 |
| Ireland | 1992 | 238.06 | 2.46 | 429.06 | 1147.35 | 138.69 | 99.37 | 0.01 | 0.21 | 2.67 | 0.72 | 25.65 |
| Ireland | 1993 | 253.32 | 2.61 | 432.03 | 1154.49 | 145.43 | 107.89 | 0.01 | 0.22 | 2.67 | 0.74 | 27.54 |
| Ireland | 1994 | 268.35 | 2.79 | 434.73 | 1159.61 | 150.56 | 117.79 | 0.01 | 0.23 | 2.67 | 0.78 | 29.79 |
| Ireland | 1995 | 289.86 | 3.08 | 437.11 | 1162.78 | 154.96 | 134.90 | 0.01 | 0.25 | 2.66 | 0.87 | 33.78 |
| Ireland | 1996 | 307.75 | 3.33 | 439.62 | 1163.46 | 158.65 | 149.10 | 0.01 | 0.26 | 2.65 | 0.94 | 37.00 |
| Ireland | 1997 | 321.32 | 3.47 | 442.70 | 1162.96 | 162.04 | 159.28 | 0.01 | 0.28 | 2.63 | 0.98 | 39.07 |
| Ireland | 1998 | 330.67 | 3.58 | 445.99 | 1163.21 | 165.18 | 165.49 | 0.01 | 0.28 | 2.61 | 1.00 | 40.34 |
| Ireland | 1999 | 341.83 | 3.71 | 449.18 | 1165.94 | 168.22 | 173.62 | 0.01 | 0.29 | 2.60 | 1.03 | 41.91 |
| Ireland | 2000 | 345.94 | 3.67 | 451.90 | 1173.09 | 171.25 | 174.69 | 0.01 | 0.29 | 2.60 | 1.02 | 41.57 |
| Ireland | 2001 | 347.00 | 3.58 | 454.38 | 1185.26 | 175.96 | 171.04 | 0.01 | 0.29 | 2.61 | 0.97 | 40.14 |
| Ireland | 2002 | 349.88 | 3.52 | 456.91 | 1200.53 | 182.06 | 167.82 | 0.01 | 0.29 | 2.63 | 0.92 | 38.86 |
| Ireland | 2003 | 347.72 | 3.38 | 459.26 | 1215.93 | 188.05 | 159.67 | 0.01 | 0.29 | 2.65 | 0.85 | 36.61 |
| Ireland | 2004 | 359.03 | 3.49 | 461.19 | 1228.45 | 193.55 | 165.48 | 0.01 | 0.29 | 2.66 | 0.85 | 37.62 |
| Ireland | 2005 | 374.29 | 3.70 | 462.39 | 1234.80 | 195.81 | 178.47 | 0.01 | 0.30 | 2.67 | 0.91 | 40.14 |
| Ireland | 2006 | 382.09 | 3.86 | 462.68 | 1231.58 | 193.77 | 188.32 | 0.01 | 0.31 | 2.66 | 0.97 | 42.33 |
| Ireland | 2007 | 386.58 | 4.01 | 462.14 | 1220.24 | 189.20 | 197.39 | 0.01 | 0.32 | 2.64 | 1.04 | 44.61 |
| Ireland | 2008 | 390.21 | 4.18 | 461.31 | 1206.33 | 183.22 | 206.99 | 0.01 | 0.32 | 2.61 | 1.13 | 47.22 |
| Ireland | 2009 | 396.91 | 4.40 | 460.58 | 1194.84 | 178.42 | 218.49 | 0.01 | 0.33 | 2.59 | 1.22 | 50.34 |
| Ireland | 2010 | 377.92 | 4.12 | 460.36 | 1190.77 | 176.41 | 201.51 | 0.01 | 0.32 | 2.59 | 1.14 | 46.77 |
| Ireland | 2011 | 380.92 | 4.14 | 459.68 | 1195.21 | 178.22 | 202.70 | 0.01 | 0.32 | 2.60 | 1.14 | 46.90 |
| Ireland | 2012 | 373.97 | 3.94 | 457.79 | 1204.28 | 182.14 | 191.83 | 0.01 | 0.31 | 2.63 | 1.05 | 44.17 |
| Ireland | 2013 | 373.27 | 3.85 | 455.38 | 1215.96 | 187.79 | 185.48 | 0.01 | 0.31 | 2.67 | 0.99 | 42.57 |
| Ireland | 2014 | 378.52 | 3.84 | 453.25 | 1228.15 | 193.38 | 185.14 | 0.01 | 0.31 | 2.71 | 0.96 | 42.20 |
| Ireland | 2015 | 383.88 | 3.86 | 452.14 | 1238.57 | 199.23 | 184.66 | 0.01 | 0.31 | 2.74 | 0.93 | 41.98 |
| Ireland | 2016 | 390.63 | 3.90 | 453.21 | 1247.92 | 205.62 | 185.01 | 0.01 | 0.31 | 2.75 | 0.90 | 41.93 |
| Ireland | 2017 | 394.51 | 3.85 | 456.19 | 1257.99 | 213.70 | 180.81 | 0.01 | 0.31 | 2.76 | 0.85 | 40.76 |
| Ireland | 2018 | 416.23 | 4.12 | 459.51 | 1268.71 | 221.43 | 194.79 | 0.01 | 0.33 | 2.76 | 0.88 | 43.40 |
| Ireland | 2019 | 434.38 | 4.35 | 461.70 | 1280.01 | 227.16 | 207.22 | 0.01 | 0.34 | 2.77 | 0.91 | 45.68 |
| Ireland | 2020 | 418.91 | 3.99 | 458.12 | 1291.61 | 230.83 | 188.08 | 0.01 | 0.32 | 2.82 | 0.81 | 41.56 |
| Ireland | 2021 | 424.79 | 3.99 | 461.29 | 1299.72 | 234.35 | 190.45 | 0.01 | 0.33 | 2.82 | 0.81 | 41.59 |
| Israel | 1990 | 295.12 | 7.29 | 594.83 | 746.92 | 99.38 | 195.74 | 0.01 | 0.40 | 1.26 | 1.97 | 72.38 |
| Israel | 1991 | 292.93 | 7.19 | 583.76 | 748.03 | 100.19 | 192.74 | 0.01 | 0.39 | 1.28 | 1.92 | 71.54 |
| Israel | 1992 | 289.26 | 6.93 | 572.46 | 748.90 | 100.52 | 188.74 | 0.01 | 0.39 | 1.31 | 1.88 | 70.02 |
| Israel | 1993 | 269.78 | 5.98 | 560.98 | 749.48 | 101.31 | 168.47 | 0.01 | 0.36 | 1.34 | 1.66 | 61.76 |
| Israel | 1994 | 262.01 | 5.61 | 549.41 | 749.66 | 101.47 | 160.54 | 0.01 | 0.35 | 1.36 | 1.58 | 58.83 |
| Israel | 1995 | 262.82 | 5.61 | 537.86 | 749.34 | 101.68 | 161.14 | 0.01 | 0.35 | 1.39 | 1.58 | 59.40 |
| Israel | 1996 | 282.18 | 6.42 | 525.58 | 748.13 | 101.72 | 180.46 | 0.01 | 0.38 | 1.42 | 1.77 | 67.98 |
| Israel | 1997 | 270.39 | 5.61 | 512.40 | 745.95 | 101.79 | 168.60 | 0.01 | 0.36 | 1.46 | 1.66 | 62.42 |
| Israel | 1998 | 258.18 | 4.89 | 499.21 | 743.60 | 102.18 | 155.99 | 0.01 | 0.35 | 1.49 | 1.53 | 56.86 |
| Israel | 1999 | 254.78 | 4.67 | 486.88 | 741.71 | 102.04 | 152.75 | 0.01 | 0.34 | 1.52 | 1.50 | 55.72 |
| Israel | 2000 | 248.74 | 4.09 | 476.33 | 740.98 | 102.59 | 146.16 | 0.01 | 0.34 | 1.56 | 1.42 | 51.88 |
| Israel | 2001 | 246.87 | 3.91 | 465.34 | 740.96 | 102.94 | 143.93 | 0.01 | 0.33 | 1.59 | 1.40 | 50.90 |
| Israel | 2002 | 244.76 | 3.85 | 452.42 | 740.67 | 103.15 | 141.61 | 0.01 | 0.33 | 1.64 | 1.37 | 50.53 |
| Israel | 2003 | 238.60 | 3.75 | 439.51 | 740.35 | 103.58 | 135.02 | 0.01 | 0.32 | 1.68 | 1.30 | 48.95 |
| Israel | 2004 | 234.78 | 3.68 | 428.53 | 740.05 | 103.85 | 130.94 | 0.01 | 0.32 | 1.73 | 1.26 | 48.01 |
| Israel | 2005 | 227.76 | 3.44 | 421.39 | 739.91 | 104.44 | 123.33 | 0.01 | 0.31 | 1.76 | 1.18 | 45.21 |
| Israel | 2006 | 222.95 | 3.35 | 417.17 | 742.86 | 104.60 | 118.35 | 0.01 | 0.30 | 1.78 | 1.13 | 43.64 |
| Israel | 2007 | 222.94 | 3.40 | 413.70 | 750.11 | 104.95 | 117.99 | 0.01 | 0.30 | 1.81 | 1.12 | 43.74 |
| Israel | 2008 | 223.08 | 3.52 | 411.15 | 759.12 | 105.06 | 118.02 | 0.01 | 0.29 | 1.85 | 1.12 | 44.17 |
| Israel | 2009 | 222.46 | 3.54 | 409.62 | 767.28 | 105.35 | 117.11 | 0.01 | 0.29 | 1.87 | 1.11 | 43.92 |
| Israel | 2010 | 218.62 | 3.44 | 409.28 | 772.11 | 105.07 | 113.54 | 0.01 | 0.28 | 1.89 | 1.08 | 42.47 |
| Israel | 2011 | 211.09 | 3.26 | 409.96 | 773.96 | 104.67 | 106.41 | 0.01 | 0.27 | 1.89 | 1.02 | 39.75 |
| Israel | 2012 | 205.60 | 3.10 | 411.18 | 775.02 | 104.15 | 101.45 | 0.01 | 0.27 | 1.88 | 0.97 | 37.72 |
| Israel | 2013 | 206.68 | 3.15 | 412.60 | 775.36 | 103.25 | 103.42 | 0.01 | 0.27 | 1.88 | 1.00 | 38.42 |
| Israel | 2014 | 209.51 | 3.26 | 413.92 | 775.09 | 102.38 | 107.13 | 0.01 | 0.27 | 1.87 | 1.05 | 39.90 |
| Israel | 2015 | 214.06 | 3.41 | 414.83 | 774.41 | 101.98 | 112.08 | 0.01 | 0.28 | 1.87 | 1.10 | 41.90 |
| Israel | 2016 | 217.66 | 3.52 | 414.83 | 770.23 | 100.90 | 116.76 | 0.01 | 0.28 | 1.86 | 1.16 | 43.80 |
| Israel | 2017 | 227.08 | 3.90 | 413.91 | 761.41 | 100.05 | 127.03 | 0.01 | 0.30 | 1.84 | 1.27 | 48.64 |
| Israel | 2018 | 235.47 | 4.24 | 412.52 | 750.95 | 99.01 | 136.46 | 0.01 | 0.31 | 1.82 | 1.38 | 53.20 |
| Israel | 2019 | 236.17 | 4.23 | 411.07 | 741.93 | 98.48 | 137.69 | 0.01 | 0.32 | 1.80 | 1.40 | 53.87 |
| Israel | 2020 | 223.67 | 3.94 | 404.39 | 713.10 | 97.44 | 126.22 | 0.01 | 0.31 | 1.76 | 1.30 | 50.77 |
| Israel | 2021 | 217.98 | 3.79 | 403.63 | 715.03 | 97.33 | 120.64 | 0.01 | 0.30 | 1.77 | 1.24 | 48.56 |
| Italy | 1990 | 278.48 | 2.39 | 408.85 | 1289.45 | 160.63 | 117.85 | 0.01 | 0.22 | 3.15 | 0.73 | 26.66 |
| Italy | 1991 | 297.80 | 2.59 | 410.81 | 1299.46 | 168.43 | 129.37 | 0.01 | 0.23 | 3.16 | 0.77 | 29.11 |
| Italy | 1992 | 302.70 | 2.58 | 411.69 | 1305.24 | 175.00 | 127.71 | 0.01 | 0.23 | 3.17 | 0.73 | 28.60 |
| Italy | 1993 | 301.07 | 2.51 | 411.65 | 1306.41 | 180.28 | 120.79 | 0.01 | 0.23 | 3.17 | 0.67 | 27.18 |
| Italy | 1994 | 305.15 | 2.55 | 410.81 | 1302.52 | 183.73 | 121.42 | 0.01 | 0.23 | 3.17 | 0.66 | 27.53 |
| Italy | 1995 | 314.43 | 2.74 | 409.32 | 1293.16 | 185.29 | 129.13 | 0.01 | 0.24 | 3.16 | 0.70 | 29.77 |
| Italy | 1996 | 315.41 | 2.79 | 401.76 | 1273.68 | 185.06 | 130.36 | 0.01 | 0.25 | 3.17 | 0.70 | 30.75 |
| Italy | 1997 | 306.09 | 2.68 | 386.20 | 1244.85 | 183.45 | 122.65 | 0.01 | 0.25 | 3.22 | 0.67 | 30.05 |
| Italy | 1998 | 296.90 | 2.59 | 367.96 | 1214.38 | 181.28 | 115.62 | 0.01 | 0.24 | 3.30 | 0.64 | 29.64 |
| Italy | 1999 | 284.76 | 2.41 | 352.38 | 1189.95 | 179.03 | 105.74 | 0.01 | 0.24 | 3.38 | 0.59 | 28.14 |
| Italy | 2000 | 273.22 | 2.22 | 344.80 | 1179.23 | 177.26 | 95.96 | 0.01 | 0.23 | 3.42 | 0.54 | 25.93 |
| Italy | 2001 | 261.66 | 2.03 | 343.89 | 1184.22 | 175.92 | 85.74 | 0.01 | 0.22 | 3.44 | 0.49 | 23.22 |
| Italy | 2002 | 251.67 | 1.85 | 344.29 | 1197.63 | 174.92 | 76.75 | 0.01 | 0.21 | 3.48 | 0.44 | 20.58 |
| Italy | 2003 | 241.25 | 1.57 | 345.24 | 1213.11 | 173.77 | 67.47 | 0.00 | 0.20 | 3.51 | 0.39 | 17.26 |
| Italy | 2004 | 236.79 | 1.52 | 345.98 | 1224.32 | 172.04 | 64.75 | 0.00 | 0.19 | 3.54 | 0.38 | 16.31 |
| Italy | 2005 | 231.73 | 1.46 | 345.76 | 1224.85 | 169.57 | 62.16 | 0.00 | 0.19 | 3.54 | 0.37 | 15.51 |
| Italy | 2006 | 225.65 | 1.36 | 344.93 | 1217.24 | 166.65 | 59.00 | 0.00 | 0.19 | 3.53 | 0.35 | 14.50 |
| Italy | 2007 | 220.47 | 1.31 | 344.26 | 1208.34 | 163.77 | 56.70 | 0.00 | 0.18 | 3.51 | 0.35 | 13.86 |
| Italy | 2008 | 214.59 | 1.28 | 343.62 | 1198.14 | 160.85 | 53.74 | 0.00 | 0.18 | 3.49 | 0.33 | 13.29 |
| Italy | 2009 | 208.79 | 1.27 | 342.88 | 1186.62 | 157.57 | 51.22 | 0.00 | 0.18 | 3.46 | 0.33 | 12.85 |
| Italy | 2010 | 203.09 | 1.26 | 341.90 | 1173.77 | 153.92 | 49.18 | 0.00 | 0.17 | 3.43 | 0.32 | 12.59 |
| Italy | 2011 | 195.95 | 1.24 | 339.42 | 1149.65 | 148.40 | 47.55 | 0.00 | 0.17 | 3.39 | 0.32 | 12.35 |
| Italy | 2012 | 188.17 | 1.27 | 335.07 | 1110.83 | 140.49 | 47.68 | 0.00 | 0.17 | 3.32 | 0.34 | 12.86 |
| Italy | 2013 | 178.45 | 1.24 | 330.13 | 1067.08 | 131.68 | 46.76 | 0.00 | 0.17 | 3.23 | 0.36 | 12.92 |
| Italy | 2014 | 168.47 | 1.21 | 325.86 | 1028.19 | 123.77 | 44.70 | 0.00 | 0.16 | 3.16 | 0.36 | 12.69 |
| Italy | 2015 | 162.93 | 1.22 | 323.48 | 1003.93 | 118.29 | 44.64 | 0.00 | 0.16 | 3.10 | 0.38 | 12.96 |
| Italy | 2016 | 158.90 | 1.23 | 322.27 | 984.28 | 114.41 | 44.49 | 0.00 | 0.16 | 3.05 | 0.39 | 13.22 |
| Italy | 2017 | 155.91 | 1.24 | 320.95 | 958.64 | 110.82 | 45.09 | 0.00 | 0.16 | 2.99 | 0.41 | 13.71 |
| Italy | 2018 | 152.77 | 1.23 | 319.96 | 936.17 | 107.83 | 44.94 | 0.00 | 0.16 | 2.93 | 0.42 | 13.87 |
| Italy | 2019 | 149.15 | 1.18 | 319.75 | 926.07 | 106.18 | 42.97 | 0.00 | 0.16 | 2.90 | 0.40 | 13.22 |
| Italy | 2020 | 146.92 | 1.08 | 321.47 | 957.70 | 109.44 | 37.49 | 0.00 | 0.15 | 2.98 | 0.34 | 10.96 |
| Italy | 2021 | 151.07 | 1.12 | 320.18 | 951.96 | 110.71 | 40.36 | 0.00 | 0.16 | 2.97 | 0.36 | 11.99 |
| Latvia | 1990 | 265.88 | 2.43 | 390.87 | 907.55 | 152.89 | 112.99 | 0.01 | 0.29 | 2.32 | 0.74 | 33.01 |
| Latvia | 1991 | 278.56 | 2.57 | 393.70 | 929.59 | 157.44 | 121.12 | 0.01 | 0.30 | 2.36 | 0.77 | 34.63 |
| Latvia | 1992 | 291.16 | 2.72 | 396.03 | 948.60 | 161.92 | 129.24 | 0.01 | 0.31 | 2.40 | 0.80 | 36.30 |
| Latvia | 1993 | 314.19 | 3.12 | 397.68 | 963.94 | 165.37 | 148.82 | 0.01 | 0.33 | 2.42 | 0.90 | 41.25 |
| Latvia | 1994 | 331.83 | 3.42 | 398.51 | 974.94 | 168.23 | 163.60 | 0.01 | 0.34 | 2.45 | 0.97 | 44.98 |
| Latvia | 1995 | 323.71 | 3.22 | 398.41 | 981.26 | 170.26 | 153.45 | 0.01 | 0.33 | 2.46 | 0.90 | 42.04 |
| Latvia | 1996 | 306.99 | 2.85 | 396.86 | 982.09 | 171.71 | 135.28 | 0.01 | 0.31 | 2.47 | 0.79 | 37.19 |
| Latvia | 1997 | 305.86 | 2.80 | 394.03 | 978.79 | 173.22 | 132.64 | 0.01 | 0.31 | 2.48 | 0.77 | 36.66 |
| Latvia | 1998 | 320.61 | 3.07 | 390.70 | 973.97 | 174.26 | 146.34 | 0.01 | 0.33 | 2.49 | 0.84 | 40.54 |
| Latvia | 1999 | 327.22 | 3.17 | 387.69 | 970.38 | 175.57 | 151.65 | 0.01 | 0.34 | 2.50 | 0.86 | 42.15 |
| Latvia | 2000 | 328.38 | 3.17 | 385.79 | 970.88 | 176.13 | 152.26 | 0.01 | 0.34 | 2.52 | 0.86 | 42.33 |
| Latvia | 2001 | 333.15 | 3.25 | 384.47 | 976.52 | 176.79 | 156.36 | 0.01 | 0.34 | 2.54 | 0.88 | 43.35 |
| Latvia | 2002 | 327.34 | 3.15 | 382.89 | 984.53 | 176.57 | 150.77 | 0.01 | 0.33 | 2.57 | 0.85 | 41.80 |
| Latvia | 2003 | 317.93 | 3.00 | 381.32 | 992.32 | 176.26 | 141.67 | 0.01 | 0.32 | 2.60 | 0.80 | 39.39 |
| Latvia | 2004 | 320.59 | 3.07 | 380.09 | 997.35 | 175.78 | 144.80 | 0.01 | 0.32 | 2.62 | 0.82 | 40.23 |
| Latvia | 2005 | 336.66 | 3.43 | 379.49 | 996.88 | 174.28 | 162.38 | 0.01 | 0.34 | 2.63 | 0.93 | 45.07 |
| Latvia | 2006 | 348.21 | 3.74 | 379.37 | 985.66 | 170.95 | 177.26 | 0.01 | 0.35 | 2.60 | 1.04 | 49.52 |
| Latvia | 2007 | 342.08 | 3.73 | 379.53 | 964.84 | 165.34 | 176.74 | 0.01 | 0.35 | 2.54 | 1.07 | 50.05 |
| Latvia | 2008 | 314.48 | 3.28 | 380.17 | 941.17 | 158.95 | 155.53 | 0.01 | 0.33 | 2.48 | 0.98 | 44.72 |
| Latvia | 2009 | 289.36 | 2.89 | 381.47 | 921.44 | 153.61 | 135.75 | 0.01 | 0.31 | 2.42 | 0.88 | 39.63 |
| Latvia | 2010 | 272.66 | 2.62 | 383.65 | 912.45 | 151.71 | 120.95 | 0.01 | 0.30 | 2.38 | 0.80 | 35.62 |
| Latvia | 2011 | 260.55 | 2.41 | 386.92 | 913.59 | 151.82 | 108.73 | 0.01 | 0.29 | 2.36 | 0.72 | 32.12 |
| Latvia | 2012 | 261.32 | 2.39 | 391.08 | 918.51 | 153.17 | 108.16 | 0.01 | 0.28 | 2.35 | 0.71 | 31.69 |
| Latvia | 2013 | 262.93 | 2.38 | 395.73 | 925.44 | 155.43 | 107.50 | 0.01 | 0.28 | 2.34 | 0.69 | 31.23 |
| Latvia | 2014 | 264.33 | 2.37 | 400.47 | 932.63 | 157.85 | 106.48 | 0.01 | 0.28 | 2.33 | 0.67 | 30.74 |
| Latvia | 2015 | 265.89 | 2.38 | 404.85 | 938.36 | 160.03 | 105.86 | 0.01 | 0.28 | 2.32 | 0.66 | 30.41 |
| Latvia | 2016 | 269.81 | 2.43 | 409.26 | 939.12 | 160.93 | 108.88 | 0.01 | 0.29 | 2.29 | 0.68 | 31.06 |
| Latvia | 2017 | 277.00 | 2.57 | 413.82 | 935.75 | 161.94 | 115.06 | 0.01 | 0.30 | 2.26 | 0.71 | 32.81 |
| Latvia | 2018 | 278.09 | 2.60 | 417.69 | 932.93 | 162.49 | 115.60 | 0.01 | 0.30 | 2.23 | 0.71 | 32.97 |
| Latvia | 2019 | 277.10 | 2.53 | 419.98 | 935.22 | 163.66 | 113.44 | 0.01 | 0.30 | 2.23 | 0.69 | 32.16 |
| Latvia | 2020 | 277.38 | 2.47 | 422.97 | 950.39 | 168.04 | 109.34 | 0.01 | 0.29 | 2.25 | 0.65 | 30.79 |
| Latvia | 2021 | 294.66 | 2.71 | 425.68 | 961.92 | 172.33 | 122.34 | 0.01 | 0.31 | 2.26 | 0.71 | 33.83 |
| Lithuania | 1990 | 237.64 | 2.47 | 353.04 | 635.42 | 122.95 | 114.69 | 0.01 | 0.37 | 1.80 | 0.93 | 43.41 |
| Lithuania | 1991 | 251.07 | 2.67 | 353.20 | 651.26 | 125.30 | 125.78 | 0.01 | 0.39 | 1.84 | 1.00 | 46.51 |
| Lithuania | 1992 | 256.81 | 2.73 | 353.05 | 664.49 | 127.43 | 129.38 | 0.01 | 0.39 | 1.88 | 1.02 | 47.10 |
| Lithuania | 1993 | 272.51 | 3.02 | 352.54 | 674.67 | 128.71 | 143.80 | 0.01 | 0.40 | 1.91 | 1.12 | 51.68 |
| Lithuania | 1994 | 284.91 | 3.25 | 351.60 | 681.36 | 129.73 | 155.18 | 0.01 | 0.42 | 1.94 | 1.20 | 55.30 |
| Lithuania | 1995 | 280.47 | 3.14 | 350.17 | 684.12 | 130.33 | 150.14 | 0.01 | 0.41 | 1.95 | 1.15 | 53.48 |
| Lithuania | 1996 | 271.13 | 2.94 | 347.72 | 681.30 | 130.24 | 140.90 | 0.01 | 0.40 | 1.96 | 1.08 | 50.43 |
| Lithuania | 1997 | 264.20 | 2.80 | 344.33 | 673.49 | 129.62 | 134.59 | 0.01 | 0.39 | 1.96 | 1.04 | 48.71 |
| Lithuania | 1998 | 263.16 | 2.81 | 340.81 | 663.64 | 128.61 | 134.55 | 0.01 | 0.40 | 1.95 | 1.05 | 49.35 |
| Lithuania | 1999 | 264.51 | 2.86 | 338.00 | 654.80 | 127.43 | 137.08 | 0.01 | 0.40 | 1.94 | 1.08 | 50.80 |
| Lithuania | 2000 | 265.81 | 2.89 | 336.73 | 649.96 | 126.45 | 139.36 | 0.01 | 0.41 | 1.93 | 1.10 | 51.79 |
| Lithuania | 2001 | 269.54 | 2.99 | 336.62 | 645.77 | 124.61 | 144.92 | 0.01 | 0.42 | 1.92 | 1.16 | 53.96 |
| Lithuania | 2002 | 261.69 | 2.91 | 336.81 | 638.55 | 121.35 | 140.34 | 0.01 | 0.41 | 1.90 | 1.16 | 52.83 |
| Lithuania | 2003 | 263.99 | 3.01 | 337.36 | 630.63 | 117.49 | 146.50 | 0.01 | 0.42 | 1.87 | 1.25 | 55.49 |
| Lithuania | 2004 | 271.67 | 3.21 | 338.35 | 624.47 | 114.35 | 157.31 | 0.01 | 0.44 | 1.85 | 1.38 | 59.85 |
| Lithuania | 2005 | 297.18 | 3.73 | 339.87 | 622.52 | 113.30 | 183.88 | 0.01 | 0.48 | 1.83 | 1.62 | 69.75 |
| Lithuania | 2006 | 323.20 | 4.21 | 342.67 | 626.08 | 114.38 | 208.83 | 0.01 | 0.52 | 1.83 | 1.83 | 78.40 |
| Lithuania | 2007 | 343.29 | 4.55 | 347.08 | 634.11 | 116.86 | 226.44 | 0.01 | 0.54 | 1.83 | 1.94 | 83.63 |
| Lithuania | 2008 | 332.32 | 4.26 | 352.52 | 645.40 | 120.53 | 211.79 | 0.01 | 0.51 | 1.83 | 1.76 | 76.84 |
| Lithuania | 2009 | 305.31 | 3.64 | 358.43 | 658.88 | 125.19 | 180.12 | 0.01 | 0.46 | 1.84 | 1.44 | 64.20 |
| Lithuania | 2010 | 297.49 | 3.41 | 364.23 | 673.43 | 130.33 | 167.16 | 0.01 | 0.44 | 1.85 | 1.28 | 58.57 |
| Lithuania | 2011 | 297.27 | 3.27 | 371.69 | 694.61 | 137.84 | 159.43 | 0.01 | 0.43 | 1.87 | 1.16 | 54.43 |
| Lithuania | 2012 | 314.18 | 3.37 | 381.87 | 725.66 | 149.34 | 164.85 | 0.01 | 0.43 | 1.90 | 1.10 | 54.00 |
| Lithuania | 2013 | 335.17 | 3.51 | 393.17 | 762.05 | 162.55 | 172.62 | 0.01 | 0.44 | 1.94 | 1.06 | 54.07 |
| Lithuania | 2014 | 354.20 | 3.64 | 403.94 | 799.14 | 175.89 | 178.30 | 0.01 | 0.44 | 1.98 | 1.01 | 53.68 |
| Lithuania | 2015 | 372.60 | 3.78 | 412.52 | 832.25 | 188.39 | 184.21 | 0.01 | 0.45 | 2.02 | 0.98 | 53.76 |
| Lithuania | 2016 | 382.64 | 3.78 | 419.38 | 864.13 | 200.79 | 181.85 | 0.01 | 0.44 | 2.06 | 0.91 | 51.96 |
| Lithuania | 2017 | 378.20 | 3.44 | 425.88 | 897.35 | 214.91 | 163.29 | 0.01 | 0.42 | 2.11 | 0.76 | 46.20 |
| Lithuania | 2018 | 387.09 | 3.37 | 431.75 | 927.05 | 228.05 | 159.05 | 0.01 | 0.42 | 2.15 | 0.70 | 44.39 |
| Lithuania | 2019 | 389.44 | 3.26 | 436.68 | 948.02 | 236.29 | 153.14 | 0.01 | 0.41 | 2.17 | 0.65 | 42.40 |
| Lithuania | 2020 | 390.14 | 3.21 | 443.97 | 976.43 | 241.53 | 148.61 | 0.01 | 0.40 | 2.20 | 0.62 | 40.62 |
| Lithuania | 2021 | 407.54 | 3.39 | 448.95 | 994.27 | 248.35 | 159.18 | 0.01 | 0.41 | 2.21 | 0.64 | 42.43 |
| Luxembourg | 1990 | 325.62 | 4.04 | 455.52 | 1109.37 | 163.81 | 161.80 | 0.01 | 0.29 | 2.44 | 0.99 | 42.58 |
| Luxembourg | 1991 | 356.17 | 4.37 | 457.45 | 1144.73 | 175.98 | 180.18 | 0.01 | 0.31 | 2.50 | 1.02 | 45.82 |
| Luxembourg | 1992 | 378.84 | 4.54 | 459.25 | 1177.35 | 187.40 | 191.44 | 0.01 | 0.32 | 2.56 | 1.02 | 47.21 |
| Luxembourg | 1993 | 399.30 | 4.71 | 460.59 | 1204.96 | 196.66 | 202.64 | 0.01 | 0.33 | 2.62 | 1.03 | 48.71 |
| Luxembourg | 1994 | 418.27 | 4.88 | 461.18 | 1225.94 | 203.75 | 214.51 | 0.01 | 0.34 | 2.66 | 1.05 | 50.49 |
| Luxembourg | 1995 | 425.92 | 4.94 | 460.81 | 1238.42 | 208.01 | 217.91 | 0.01 | 0.34 | 2.69 | 1.05 | 50.91 |
| Luxembourg | 1996 | 429.38 | 4.97 | 460.30 | 1246.40 | 210.96 | 218.42 | 0.01 | 0.34 | 2.71 | 1.04 | 50.89 |
| Luxembourg | 1997 | 417.37 | 4.74 | 460.40 | 1254.19 | 213.73 | 203.64 | 0.01 | 0.33 | 2.72 | 0.95 | 47.73 |
| Luxembourg | 1998 | 408.57 | 4.54 | 460.69 | 1260.86 | 215.93 | 192.64 | 0.01 | 0.32 | 2.74 | 0.89 | 45.27 |
| Luxembourg | 1999 | 405.00 | 4.37 | 460.73 | 1265.74 | 217.17 | 187.83 | 0.01 | 0.32 | 2.75 | 0.86 | 43.75 |
| Luxembourg | 2000 | 403.86 | 4.18 | 460.16 | 1268.48 | 218.19 | 185.67 | 0.01 | 0.32 | 2.76 | 0.85 | 42.54 |
| Luxembourg | 2001 | 394.06 | 3.97 | 457.57 | 1265.02 | 216.38 | 177.68 | 0.01 | 0.31 | 2.76 | 0.82 | 40.73 |
| Luxembourg | 2002 | 383.02 | 3.88 | 452.89 | 1254.85 | 211.87 | 171.15 | 0.01 | 0.31 | 2.77 | 0.81 | 39.86 |
| Luxembourg | 2003 | 374.22 | 3.84 | 447.68 | 1242.20 | 206.09 | 168.13 | 0.01 | 0.30 | 2.77 | 0.82 | 39.76 |
| Luxembourg | 2004 | 370.66 | 3.89 | 443.30 | 1230.57 | 200.78 | 169.88 | 0.01 | 0.30 | 2.78 | 0.85 | 40.62 |
| Luxembourg | 2005 | 368.01 | 3.86 | 441.16 | 1223.76 | 197.77 | 170.25 | 0.01 | 0.30 | 2.77 | 0.86 | 40.76 |
| Luxembourg | 2006 | 364.72 | 3.82 | 441.09 | 1220.55 | 196.11 | 168.62 | 0.01 | 0.30 | 2.77 | 0.86 | 40.43 |
| Luxembourg | 2007 | 361.09 | 3.79 | 441.73 | 1218.09 | 194.99 | 166.10 | 0.01 | 0.30 | 2.76 | 0.85 | 40.00 |
| Luxembourg | 2008 | 355.08 | 3.72 | 442.74 | 1216.37 | 194.63 | 160.45 | 0.01 | 0.29 | 2.75 | 0.82 | 38.88 |
| Luxembourg | 2009 | 350.20 | 3.69 | 443.81 | 1214.36 | 193.78 | 156.43 | 0.01 | 0.29 | 2.74 | 0.81 | 38.15 |
| Luxembourg | 2010 | 343.61 | 3.69 | 444.42 | 1210.59 | 192.09 | 151.52 | 0.01 | 0.28 | 2.72 | 0.79 | 37.55 |
| Luxembourg | 2011 | 333.61 | 3.60 | 444.68 | 1202.36 | 188.25 | 145.35 | 0.01 | 0.28 | 2.70 | 0.77 | 36.40 |
| Luxembourg | 2012 | 323.61 | 3.53 | 444.99 | 1188.87 | 182.51 | 141.10 | 0.01 | 0.27 | 2.67 | 0.77 | 35.71 |
| Luxembourg | 2013 | 313.26 | 3.48 | 445.59 | 1173.90 | 176.21 | 137.06 | 0.01 | 0.27 | 2.63 | 0.78 | 35.16 |
| Luxembourg | 2014 | 306.98 | 3.55 | 446.62 | 1160.77 | 170.71 | 136.27 | 0.01 | 0.26 | 2.60 | 0.80 | 35.61 |
| Luxembourg | 2015 | 306.84 | 3.65 | 448.28 | 1152.88 | 167.63 | 139.21 | 0.01 | 0.27 | 2.57 | 0.83 | 36.68 |
| Luxembourg | 2016 | 305.60 | 3.70 | 451.79 | 1149.58 | 165.88 | 139.72 | 0.01 | 0.27 | 2.54 | 0.84 | 36.93 |
| Luxembourg | 2017 | 306.23 | 3.82 | 456.75 | 1146.90 | 164.30 | 141.93 | 0.01 | 0.27 | 2.51 | 0.86 | 37.72 |
| Luxembourg | 2018 | 310.54 | 3.90 | 461.35 | 1145.23 | 163.29 | 147.25 | 0.01 | 0.27 | 2.48 | 0.90 | 38.79 |
| Luxembourg | 2019 | 307.54 | 3.80 | 463.64 | 1144.44 | 162.58 | 144.96 | 0.01 | 0.27 | 2.47 | 0.89 | 37.94 |
| Luxembourg | 2020 | 289.55 | 3.41 | 456.41 | 1144.62 | 161.72 | 127.83 | 0.01 | 0.25 | 2.51 | 0.79 | 33.74 |
| Luxembourg | 2021 | 291.82 | 3.41 | 458.61 | 1148.31 | 162.86 | 128.96 | 0.01 | 0.25 | 2.50 | 0.79 | 33.75 |
| Malta | 1990 | 170.69 | 1.83 | 349.05 | 677.20 | 108.47 | 62.22 | 0.01 | 0.25 | 1.94 | 0.57 | 25.64 |
| Malta | 1991 | 177.54 | 1.86 | 350.48 | 683.56 | 109.50 | 68.04 | 0.01 | 0.26 | 1.95 | 0.62 | 27.06 |
| Malta | 1992 | 185.54 | 1.96 | 351.66 | 690.10 | 110.82 | 74.72 | 0.01 | 0.27 | 1.96 | 0.67 | 29.05 |
| Malta | 1993 | 187.92 | 1.96 | 352.59 | 696.33 | 112.38 | 75.54 | 0.01 | 0.27 | 1.97 | 0.67 | 29.03 |
| Malta | 1994 | 193.13 | 1.99 | 353.24 | 702.29 | 113.96 | 79.17 | 0.01 | 0.28 | 1.99 | 0.69 | 29.93 |
| Malta | 1995 | 199.42 | 2.07 | 353.65 | 707.65 | 115.46 | 83.95 | 0.01 | 0.28 | 2.00 | 0.73 | 31.37 |
| Malta | 1996 | 206.09 | 2.11 | 353.48 | 718.34 | 120.26 | 85.83 | 0.01 | 0.29 | 2.03 | 0.71 | 31.72 |
| Malta | 1997 | 215.70 | 2.15 | 352.80 | 737.69 | 127.90 | 87.80 | 0.01 | 0.29 | 2.09 | 0.69 | 31.95 |
| Malta | 1998 | 225.01 | 2.12 | 352.16 | 760.62 | 137.63 | 87.38 | 0.01 | 0.30 | 2.16 | 0.63 | 31.19 |
| Malta | 1999 | 231.53 | 2.03 | 352.08 | 782.11 | 146.94 | 84.60 | 0.01 | 0.30 | 2.22 | 0.58 | 29.75 |
| Malta | 2000 | 234.86 | 1.89 | 353.10 | 797.28 | 152.87 | 81.99 | 0.01 | 0.29 | 2.26 | 0.54 | 28.16 |
| Malta | 2001 | 239.32 | 1.88 | 355.55 | 807.79 | 156.64 | 82.68 | 0.01 | 0.30 | 2.27 | 0.53 | 27.97 |
| Malta | 2002 | 242.65 | 1.90 | 358.89 | 817.97 | 159.94 | 82.71 | 0.01 | 0.30 | 2.28 | 0.52 | 27.83 |
| Malta | 2003 | 246.17 | 1.90 | 362.41 | 827.59 | 163.42 | 82.75 | 0.01 | 0.30 | 2.28 | 0.51 | 27.64 |
| Malta | 2004 | 251.30 | 1.94 | 365.41 | 835.91 | 166.42 | 84.88 | 0.01 | 0.30 | 2.29 | 0.51 | 28.04 |
| Malta | 2005 | 255.65 | 1.96 | 367.20 | 842.64 | 168.82 | 86.83 | 0.01 | 0.30 | 2.29 | 0.51 | 28.38 |
| Malta | 2006 | 260.73 | 2.00 | 368.14 | 848.46 | 170.80 | 89.93 | 0.01 | 0.31 | 2.30 | 0.53 | 29.04 |
| Malta | 2007 | 269.28 | 2.11 | 369.11 | 853.91 | 172.92 | 96.36 | 0.01 | 0.32 | 2.31 | 0.56 | 30.64 |
| Malta | 2008 | 274.79 | 2.19 | 370.07 | 858.83 | 174.69 | 100.11 | 0.01 | 0.32 | 2.32 | 0.57 | 31.64 |
| Malta | 2009 | 276.88 | 2.21 | 370.96 | 862.73 | 175.86 | 101.02 | 0.01 | 0.32 | 2.33 | 0.57 | 31.82 |
| Malta | 2010 | 276.38 | 2.19 | 371.72 | 865.44 | 176.47 | 99.91 | 0.01 | 0.32 | 2.33 | 0.57 | 31.46 |
| Malta | 2011 | 269.57 | 2.10 | 372.62 | 861.50 | 174.31 | 95.26 | 0.01 | 0.31 | 2.31 | 0.55 | 30.17 |
| Malta | 2012 | 261.31 | 2.07 | 373.73 | 849.16 | 168.19 | 93.12 | 0.01 | 0.31 | 2.27 | 0.55 | 29.68 |
| Malta | 2013 | 252.10 | 2.07 | 374.84 | 833.48 | 159.92 | 92.19 | 0.01 | 0.30 | 2.22 | 0.58 | 29.70 |
| Malta | 2014 | 248.55 | 2.15 | 375.73 | 819.75 | 152.75 | 95.80 | 0.01 | 0.30 | 2.18 | 0.63 | 30.95 |
| Malta | 2015 | 246.53 | 2.18 | 376.15 | 813.18 | 148.56 | 97.97 | 0.01 | 0.30 | 2.16 | 0.66 | 31.64 |
| Malta | 2016 | 250.07 | 2.28 | 377.25 | 821.99 | 147.52 | 102.54 | 0.01 | 0.30 | 2.18 | 0.70 | 32.79 |
| Malta | 2017 | 257.13 | 2.48 | 379.39 | 843.16 | 147.50 | 109.63 | 0.01 | 0.30 | 2.22 | 0.74 | 34.59 |
| Malta | 2018 | 260.71 | 2.53 | 381.39 | 864.63 | 148.13 | 112.58 | 0.01 | 0.30 | 2.27 | 0.76 | 34.85 |
| Malta | 2019 | 260.56 | 2.53 | 382.15 | 875.28 | 148.21 | 112.35 | 0.01 | 0.30 | 2.29 | 0.76 | 34.51 |
| Malta | 2020 | 260.98 | 2.47 | 378.12 | 876.08 | 148.35 | 112.62 | 0.01 | 0.30 | 2.32 | 0.76 | 34.38 |
| Malta | 2021 | 246.80 | 2.29 | 381.30 | 876.03 | 148.40 | 98.39 | 0.01 | 0.28 | 2.30 | 0.66 | 30.62 |
| Monaco | 1990 | 297.76 | 6.55 | 708.03 | 933.46 | 118.05 | 179.71 | 0.01 | 0.32 | 1.32 | 1.52 | 53.75 |
| Monaco | 1991 | 294.59 | 6.44 | 703.53 | 935.06 | 118.44 | 176.16 | 0.01 | 0.32 | 1.33 | 1.49 | 52.71 |
| Monaco | 1992 | 290.83 | 6.34 | 699.49 | 936.42 | 118.54 | 172.29 | 0.01 | 0.31 | 1.34 | 1.45 | 51.69 |
| Monaco | 1993 | 290.87 | 6.31 | 696.14 | 937.88 | 118.91 | 171.95 | 0.01 | 0.31 | 1.35 | 1.45 | 51.52 |
| Monaco | 1994 | 290.28 | 6.30 | 693.51 | 939.23 | 118.86 | 171.41 | 0.01 | 0.31 | 1.35 | 1.44 | 51.43 |
| Monaco | 1995 | 289.42 | 6.28 | 691.67 | 940.56 | 118.78 | 170.64 | 0.01 | 0.31 | 1.36 | 1.44 | 51.25 |
| Monaco | 1996 | 289.64 | 6.29 | 690.80 | 942.02 | 118.92 | 170.73 | 0.01 | 0.31 | 1.36 | 1.44 | 51.27 |
| Monaco | 1997 | 290.78 | 6.33 | 690.60 | 943.26 | 119.30 | 171.49 | 0.01 | 0.31 | 1.37 | 1.44 | 51.49 |
| Monaco | 1998 | 291.42 | 6.36 | 690.83 | 944.73 | 119.41 | 172.01 | 0.01 | 0.31 | 1.37 | 1.44 | 51.64 |
| Monaco | 1999 | 292.11 | 6.41 | 691.05 | 946.31 | 119.69 | 172.43 | 0.01 | 0.31 | 1.37 | 1.44 | 51.81 |
| Monaco | 2000 | 293.11 | 6.46 | 690.95 | 948.09 | 120.19 | 172.92 | 0.01 | 0.31 | 1.37 | 1.44 | 51.97 |
| Monaco | 2001 | 293.12 | 6.47 | 690.57 | 950.21 | 120.69 | 172.43 | 0.01 | 0.31 | 1.38 | 1.43 | 51.80 |
| Monaco | 2002 | 292.43 | 6.45 | 690.06 | 952.10 | 121.18 | 171.26 | 0.01 | 0.31 | 1.38 | 1.41 | 51.43 |
| Monaco | 2003 | 291.79 | 6.43 | 689.52 | 954.31 | 121.62 | 170.18 | 0.01 | 0.31 | 1.38 | 1.40 | 51.05 |
| Monaco | 2004 | 290.60 | 6.38 | 689.11 | 956.78 | 122.23 | 168.37 | 0.01 | 0.30 | 1.39 | 1.38 | 50.44 |
| Monaco | 2005 | 289.90 | 6.35 | 688.79 | 959.01 | 122.94 | 166.96 | 0.01 | 0.30 | 1.39 | 1.36 | 49.93 |
| Monaco | 2006 | 288.84 | 6.30 | 688.86 | 961.44 | 123.58 | 165.26 | 0.01 | 0.30 | 1.40 | 1.34 | 49.34 |
| Monaco | 2007 | 286.85 | 6.22 | 689.18 | 963.29 | 124.28 | 162.57 | 0.01 | 0.30 | 1.40 | 1.31 | 48.45 |
| Monaco | 2008 | 285.58 | 6.16 | 689.41 | 964.53 | 125.28 | 160.30 | 0.01 | 0.30 | 1.40 | 1.28 | 47.69 |
| Monaco | 2009 | 284.97 | 6.13 | 689.81 | 966.36 | 125.91 | 159.06 | 0.01 | 0.29 | 1.40 | 1.26 | 47.24 |
| Monaco | 2010 | 284.00 | 6.09 | 690.06 | 967.13 | 126.18 | 157.82 | 0.01 | 0.29 | 1.40 | 1.25 | 46.85 |
| Monaco | 2011 | 282.84 | 6.06 | 691.00 | 967.36 | 126.30 | 156.55 | 0.01 | 0.29 | 1.40 | 1.24 | 46.45 |
| Monaco | 2012 | 280.36 | 6.00 | 693.31 | 967.85 | 125.76 | 154.60 | 0.01 | 0.29 | 1.40 | 1.23 | 45.85 |
| Monaco | 2013 | 278.23 | 5.96 | 696.10 | 967.01 | 124.92 | 153.31 | 0.01 | 0.29 | 1.39 | 1.23 | 45.51 |
| Monaco | 2014 | 275.67 | 5.90 | 699.06 | 966.56 | 124.52 | 151.15 | 0.01 | 0.29 | 1.38 | 1.21 | 44.86 |
| Monaco | 2015 | 272.59 | 5.83 | 701.34 | 965.51 | 123.84 | 148.76 | 0.01 | 0.28 | 1.38 | 1.20 | 44.18 |
| Monaco | 2016 | 269.75 | 5.75 | 704.56 | 963.62 | 123.35 | 146.40 | 0.01 | 0.28 | 1.37 | 1.19 | 43.46 |
| Monaco | 2017 | 266.61 | 5.67 | 709.50 | 961.70 | 122.41 | 144.20 | 0.01 | 0.28 | 1.36 | 1.18 | 42.79 |
| Monaco | 2018 | 263.22 | 5.59 | 714.17 | 959.25 | 121.34 | 141.88 | 0.01 | 0.27 | 1.34 | 1.17 | 42.12 |
| Monaco | 2019 | 260.66 | 5.51 | 717.12 | 958.46 | 121.01 | 139.66 | 0.01 | 0.27 | 1.34 | 1.15 | 41.42 |
| Monaco | 2020 | 267.79 | 5.54 | 713.82 | 958.38 | 120.30 | 147.49 | 0.01 | 0.28 | 1.34 | 1.23 | 43.20 |
| Monaco | 2021 | 267.23 | 5.50 | 708.43 | 959.01 | 120.60 | 146.63 | 0.01 | 0.28 | 1.35 | 1.22 | 43.00 |
| Montenegro | 1990 | 81.28 | 0.38 | 378.67 | 526.18 | 65.17 | 16.11 | 0.00 | 0.15 | 1.39 | 0.25 | 4.87 |
| Montenegro | 1991 | 80.31 | 0.36 | 377.30 | 525.66 | 65.17 | 15.14 | 0.00 | 0.15 | 1.39 | 0.23 | 4.44 |
| Montenegro | 1992 | 80.18 | 0.36 | 376.19 | 525.17 | 65.09 | 15.09 | 0.00 | 0.15 | 1.40 | 0.23 | 4.43 |
| Montenegro | 1993 | 80.79 | 0.38 | 375.38 | 524.75 | 65.06 | 15.73 | 0.00 | 0.15 | 1.40 | 0.24 | 4.74 |
| Montenegro | 1994 | 81.18 | 0.38 | 374.86 | 524.41 | 65.15 | 16.03 | 0.00 | 0.15 | 1.40 | 0.25 | 4.92 |
| Montenegro | 1995 | 81.09 | 0.38 | 374.63 | 524.14 | 65.01 | 16.08 | 0.00 | 0.15 | 1.40 | 0.25 | 4.94 |
| Montenegro | 1996 | 81.31 | 0.39 | 374.22 | 520.87 | 64.99 | 16.32 | 0.00 | 0.16 | 1.39 | 0.25 | 5.11 |
| Montenegro | 1997 | 81.43 | 0.39 | 373.39 | 513.40 | 64.85 | 16.58 | 0.00 | 0.16 | 1.37 | 0.26 | 5.40 |
| Montenegro | 1998 | 82.00 | 0.41 | 372.57 | 504.58 | 64.80 | 17.20 | 0.00 | 0.16 | 1.35 | 0.27 | 5.92 |
| Montenegro | 1999 | 83.18 | 0.43 | 372.15 | 497.25 | 64.64 | 18.54 | 0.00 | 0.17 | 1.34 | 0.29 | 6.71 |
| Montenegro | 2000 | 83.11 | 0.43 | 372.52 | 494.23 | 64.70 | 18.40 | 0.00 | 0.17 | 1.33 | 0.28 | 6.72 |
| Montenegro | 2001 | 82.27 | 0.42 | 373.50 | 494.26 | 64.72 | 17.55 | 0.00 | 0.17 | 1.32 | 0.27 | 6.33 |
| Montenegro | 2002 | 81.56 | 0.41 | 374.61 | 494.26 | 64.85 | 16.71 | 0.00 | 0.17 | 1.32 | 0.26 | 5.96 |
| Montenegro | 2003 | 81.78 | 0.41 | 375.90 | 494.37 | 65.02 | 16.77 | 0.00 | 0.17 | 1.32 | 0.26 | 5.99 |
| Montenegro | 2004 | 81.86 | 0.40 | 377.39 | 494.69 | 65.21 | 16.65 | 0.00 | 0.17 | 1.31 | 0.26 | 5.91 |
| Montenegro | 2005 | 82.33 | 0.40 | 379.08 | 495.28 | 65.54 | 16.79 | 0.00 | 0.17 | 1.31 | 0.26 | 5.95 |
| Montenegro | 2006 | 82.49 | 0.39 | 381.69 | 497.01 | 65.99 | 16.51 | 0.00 | 0.17 | 1.30 | 0.25 | 5.79 |
| Montenegro | 2007 | 82.77 | 0.38 | 385.31 | 500.02 | 66.64 | 16.12 | 0.00 | 0.17 | 1.30 | 0.24 | 5.55 |
| Montenegro | 2008 | 83.42 | 0.38 | 389.09 | 503.44 | 67.34 | 16.08 | 0.00 | 0.17 | 1.29 | 0.24 | 5.49 |
| Montenegro | 2009 | 84.08 | 0.39 | 392.16 | 506.29 | 68.12 | 15.95 | 0.00 | 0.17 | 1.29 | 0.23 | 5.45 |
| Montenegro | 2010 | 84.02 | 0.39 | 393.68 | 507.67 | 68.43 | 15.60 | 0.00 | 0.17 | 1.29 | 0.23 | 5.30 |
| Montenegro | 2011 | 83.71 | 0.38 | 393.26 | 507.53 | 68.48 | 15.23 | 0.00 | 0.16 | 1.29 | 0.22 | 5.16 |
| Montenegro | 2012 | 83.58 | 0.38 | 391.60 | 506.66 | 68.22 | 15.35 | 0.00 | 0.16 | 1.29 | 0.23 | 5.22 |
| Montenegro | 2013 | 83.46 | 0.39 | 389.39 | 505.39 | 68.01 | 15.44 | 0.00 | 0.17 | 1.30 | 0.23 | 5.29 |
| Montenegro | 2014 | 83.11 | 0.39 | 387.34 | 504.04 | 67.75 | 15.36 | 0.00 | 0.16 | 1.30 | 0.23 | 5.28 |
| Montenegro | 2015 | 82.50 | 0.39 | 386.15 | 502.98 | 67.45 | 15.04 | 0.00 | 0.16 | 1.30 | 0.22 | 5.17 |
| Montenegro | 2016 | 82.44 | 0.39 | 385.37 | 501.73 | 67.14 | 15.30 | 0.00 | 0.16 | 1.30 | 0.23 | 5.31 |
| Montenegro | 2017 | 82.49 | 0.40 | 384.34 | 500.06 | 66.74 | 15.75 | 0.00 | 0.16 | 1.30 | 0.24 | 5.54 |
| Montenegro | 2018 | 81.84 | 0.40 | 383.42 | 498.60 | 66.13 | 15.71 | 0.00 | 0.16 | 1.30 | 0.24 | 5.52 |
| Montenegro | 2019 | 82.26 | 0.42 | 382.98 | 497.98 | 65.86 | 16.40 | 0.00 | 0.17 | 1.30 | 0.25 | 5.83 |
| Montenegro | 2020 | 81.47 | 0.40 | 384.52 | 500.27 | 66.22 | 15.25 | 0.00 | 0.16 | 1.30 | 0.23 | 5.29 |
| Montenegro | 2021 | 79.06 | 0.37 | 382.28 | 475.13 | 64.95 | 14.11 | 0.00 | 0.17 | 1.24 | 0.22 | 5.20 |
| Netherlands | 1990 | 148.04 | 1.32 | 408.16 | 1128.84 | 105.64 | 42.40 | 0.00 | 0.13 | 2.77 | 0.40 | 10.14 |
| Netherlands | 1991 | 150.75 | 1.35 | 407.45 | 1124.61 | 106.38 | 44.37 | 0.00 | 0.13 | 2.76 | 0.42 | 10.74 |
| Netherlands | 1992 | 152.71 | 1.36 | 406.76 | 1120.24 | 107.32 | 45.39 | 0.00 | 0.14 | 2.75 | 0.42 | 11.08 |
| Netherlands | 1993 | 155.22 | 1.40 | 406.13 | 1115.90 | 108.50 | 46.72 | 0.00 | 0.14 | 2.75 | 0.43 | 11.62 |
| Netherlands | 1994 | 156.73 | 1.41 | 405.59 | 1111.85 | 109.85 | 46.88 | 0.00 | 0.14 | 2.74 | 0.43 | 11.73 |
| Netherlands | 1995 | 158.74 | 1.38 | 405.15 | 1108.31 | 111.36 | 47.39 | 0.00 | 0.14 | 2.74 | 0.43 | 11.72 |
| Netherlands | 1996 | 161.95 | 1.31 | 404.67 | 1102.09 | 113.55 | 48.40 | 0.00 | 0.15 | 2.72 | 0.43 | 11.61 |
| Netherlands | 1997 | 165.05 | 1.27 | 404.01 | 1091.63 | 116.43 | 48.62 | 0.00 | 0.15 | 2.70 | 0.42 | 11.59 |
| Netherlands | 1998 | 169.39 | 1.27 | 403.30 | 1079.98 | 119.71 | 49.68 | 0.00 | 0.16 | 2.68 | 0.42 | 11.97 |
| Netherlands | 1999 | 173.38 | 1.30 | 402.60 | 1069.90 | 121.97 | 51.41 | 0.00 | 0.16 | 2.66 | 0.42 | 12.57 |
| Netherlands | 2000 | 173.97 | 1.25 | 402.02 | 1064.29 | 122.61 | 51.35 | 0.00 | 0.16 | 2.65 | 0.42 | 12.41 |
| Netherlands | 2001 | 171.54 | 1.24 | 400.60 | 1059.21 | 120.35 | 51.18 | 0.00 | 0.16 | 2.64 | 0.43 | 12.35 |
| Netherlands | 2002 | 167.05 | 1.25 | 397.88 | 1050.61 | 116.09 | 50.96 | 0.00 | 0.16 | 2.64 | 0.44 | 12.49 |
| Netherlands | 2003 | 161.09 | 1.22 | 394.77 | 1041.33 | 111.19 | 49.90 | 0.00 | 0.15 | 2.64 | 0.45 | 12.20 |
| Netherlands | 2004 | 156.83 | 1.23 | 392.13 | 1033.90 | 106.75 | 50.09 | 0.00 | 0.15 | 2.64 | 0.47 | 12.40 |
| Netherlands | 2005 | 153.27 | 1.19 | 390.85 | 1031.01 | 104.70 | 48.57 | 0.00 | 0.15 | 2.64 | 0.46 | 11.95 |
| Netherlands | 2006 | 151.97 | 1.16 | 390.45 | 1029.11 | 104.31 | 47.66 | 0.00 | 0.15 | 2.64 | 0.46 | 11.62 |
| Netherlands | 2007 | 152.10 | 1.16 | 389.93 | 1024.26 | 103.94 | 48.16 | 0.00 | 0.15 | 2.63 | 0.46 | 11.80 |
| Netherlands | 2008 | 154.11 | 1.21 | 389.39 | 1018.95 | 103.98 | 50.13 | 0.00 | 0.15 | 2.62 | 0.48 | 12.56 |
| Netherlands | 2009 | 154.56 | 1.22 | 388.95 | 1015.44 | 104.22 | 50.34 | 0.00 | 0.15 | 2.61 | 0.48 | 12.68 |
| Netherlands | 2010 | 154.83 | 1.22 | 388.72 | 1016.14 | 104.84 | 49.99 | 0.00 | 0.15 | 2.61 | 0.48 | 12.62 |
| Netherlands | 2011 | 155.93 | 1.23 | 388.26 | 1020.69 | 105.46 | 50.47 | 0.00 | 0.15 | 2.63 | 0.48 | 12.70 |
| Netherlands | 2012 | 157.23 | 1.23 | 387.31 | 1026.63 | 106.18 | 51.05 | 0.00 | 0.15 | 2.65 | 0.48 | 12.77 |
| Netherlands | 2013 | 159.23 | 1.27 | 386.20 | 1032.97 | 107.13 | 52.10 | 0.00 | 0.15 | 2.67 | 0.49 | 13.16 |
| Netherlands | 2014 | 161.51 | 1.31 | 385.31 | 1038.94 | 108.00 | 53.50 | 0.00 | 0.16 | 2.70 | 0.50 | 13.65 |
| Netherlands | 2015 | 165.60 | 1.37 | 384.98 | 1043.66 | 108.81 | 56.79 | 0.00 | 0.16 | 2.71 | 0.52 | 14.63 |
| Netherlands | 2016 | 171.58 | 1.47 | 384.03 | 1041.91 | 110.20 | 61.38 | 0.00 | 0.16 | 2.71 | 0.56 | 16.15 |
| Netherlands | 2017 | 176.89 | 1.57 | 381.90 | 1033.44 | 111.70 | 65.19 | 0.00 | 0.17 | 2.71 | 0.58 | 17.62 |
| Netherlands | 2018 | 181.38 | 1.67 | 379.91 | 1024.65 | 113.55 | 67.83 | 0.00 | 0.18 | 2.70 | 0.60 | 18.84 |
| Netherlands | 2019 | 183.78 | 1.67 | 379.39 | 1022.02 | 115.45 | 68.33 | 0.00 | 0.18 | 2.69 | 0.59 | 18.94 |
| Netherlands | 2020 | 179.59 | 1.58 | 378.09 | 1014.64 | 115.69 | 63.90 | 0.00 | 0.18 | 2.68 | 0.55 | 17.71 |
| Netherlands | 2021 | 182.62 | 1.59 | 371.91 | 982.29 | 117.82 | 64.80 | 0.00 | 0.19 | 2.64 | 0.55 | 18.50 |
| North Macedonia | 1990 | 102.88 | 0.70 | 342.66 | 470.87 | 71.33 | 31.55 | 0.00 | 0.22 | 1.37 | 0.44 | 14.56 |
| North Macedonia | 1991 | 102.48 | 0.70 | 342.00 | 470.15 | 71.28 | 31.21 | 0.00 | 0.22 | 1.37 | 0.44 | 14.48 |
| North Macedonia | 1992 | 102.93 | 0.72 | 341.52 | 469.48 | 71.14 | 31.79 | 0.00 | 0.22 | 1.37 | 0.45 | 14.84 |
| North Macedonia | 1993 | 103.02 | 0.73 | 341.19 | 468.88 | 71.35 | 31.67 | 0.00 | 0.22 | 1.37 | 0.44 | 14.89 |
| North Macedonia | 1994 | 102.48 | 0.73 | 341.01 | 468.33 | 71.36 | 31.12 | 0.00 | 0.22 | 1.37 | 0.44 | 14.68 |
| North Macedonia | 1995 | 102.37 | 0.74 | 340.93 | 467.78 | 71.41 | 30.95 | 0.00 | 0.22 | 1.37 | 0.43 | 14.70 |
| North Macedonia | 1996 | 102.79 | 0.75 | 341.17 | 467.09 | 71.76 | 31.03 | 0.00 | 0.22 | 1.37 | 0.43 | 14.86 |
| North Macedonia | 1997 | 103.85 | 0.78 | 341.86 | 466.43 | 71.88 | 31.98 | 0.00 | 0.22 | 1.36 | 0.44 | 15.40 |
| North Macedonia | 1998 | 107.38 | 0.85 | 342.86 | 466.17 | 72.29 | 35.08 | 0.00 | 0.23 | 1.36 | 0.49 | 16.97 |
| North Macedonia | 1999 | 109.99 | 0.90 | 344.05 | 466.69 | 73.24 | 36.75 | 0.00 | 0.24 | 1.36 | 0.50 | 17.86 |
| North Macedonia | 2000 | 110.86 | 0.91 | 345.28 | 468.23 | 73.90 | 36.96 | 0.00 | 0.24 | 1.36 | 0.50 | 17.95 |
| North Macedonia | 2001 | 113.47 | 0.94 | 346.60 | 470.86 | 75.07 | 38.40 | 0.00 | 0.24 | 1.36 | 0.51 | 18.59 |
| North Macedonia | 2002 | 116.09 | 0.96 | 348.20 | 474.58 | 76.26 | 39.83 | 0.00 | 0.24 | 1.36 | 0.52 | 19.14 |
| North Macedonia | 2003 | 118.81 | 0.97 | 350.03 | 479.22 | 78.26 | 40.56 | 0.00 | 0.25 | 1.37 | 0.52 | 19.33 |
| North Macedonia | 2004 | 120.01 | 0.96 | 352.00 | 484.22 | 80.40 | 39.61 | 0.00 | 0.25 | 1.38 | 0.49 | 18.80 |
| North Macedonia | 2005 | 120.85 | 0.95 | 353.98 | 488.98 | 82.18 | 38.66 | 0.00 | 0.25 | 1.38 | 0.47 | 18.32 |
| North Macedonia | 2006 | 124.13 | 0.96 | 356.91 | 496.36 | 85.15 | 38.99 | 0.00 | 0.25 | 1.39 | 0.46 | 18.37 |
| North Macedonia | 2007 | 129.25 | 0.99 | 361.08 | 507.61 | 89.38 | 39.87 | 0.00 | 0.25 | 1.41 | 0.45 | 18.58 |
| North Macedonia | 2008 | 133.49 | 0.98 | 365.47 | 519.64 | 94.34 | 39.15 | 0.00 | 0.26 | 1.42 | 0.42 | 18.10 |
| North Macedonia | 2009 | 136.42 | 0.97 | 369.06 | 529.29 | 97.68 | 38.73 | 0.00 | 0.26 | 1.43 | 0.40 | 17.76 |
| North Macedonia | 2010 | 136.65 | 0.95 | 370.80 | 533.37 | 99.14 | 37.51 | 0.00 | 0.26 | 1.44 | 0.38 | 17.22 |
| North Macedonia | 2011 | 134.64 | 0.91 | 370.37 | 532.35 | 98.90 | 35.74 | 0.00 | 0.25 | 1.44 | 0.36 | 16.51 |
| North Macedonia | 2012 | 133.32 | 0.91 | 368.58 | 529.33 | 97.64 | 35.67 | 0.00 | 0.25 | 1.44 | 0.37 | 16.53 |
| North Macedonia | 2013 | 131.87 | 0.90 | 366.15 | 525.13 | 95.98 | 35.89 | 0.00 | 0.25 | 1.43 | 0.37 | 16.60 |
| North Macedonia | 2014 | 130.60 | 0.90 | 363.79 | 520.51 | 94.37 | 36.23 | 0.00 | 0.25 | 1.43 | 0.38 | 16.77 |
| North Macedonia | 2015 | 128.69 | 0.89 | 362.22 | 516.17 | 92.61 | 36.08 | 0.00 | 0.25 | 1.43 | 0.39 | 16.74 |
| North Macedonia | 2016 | 127.25 | 0.89 | 361.07 | 510.46 | 90.78 | 36.47 | 0.00 | 0.25 | 1.41 | 0.40 | 16.95 |
| North Macedonia | 2017 | 124.13 | 0.87 | 359.76 | 502.88 | 88.61 | 35.52 | 0.00 | 0.25 | 1.40 | 0.40 | 16.67 |
| North Macedonia | 2018 | 121.47 | 0.86 | 358.63 | 495.70 | 86.68 | 34.79 | 0.00 | 0.25 | 1.38 | 0.40 | 16.51 |
| North Macedonia | 2019 | 118.67 | 0.84 | 358.05 | 491.20 | 85.18 | 33.49 | 0.00 | 0.24 | 1.37 | 0.39 | 16.01 |
| North Macedonia | 2020 | 111.13 | 0.75 | 358.48 | 484.42 | 81.92 | 29.21 | 0.00 | 0.23 | 1.35 | 0.36 | 14.05 |
| North Macedonia | 2021 | 105.52 | 0.74 | 357.42 | 470.37 | 76.18 | 29.33 | 0.00 | 0.22 | 1.32 | 0.39 | 14.14 |
| Norway | 1990 | 291.73 | 2.22 | 497.69 | 908.22 | 182.70 | 109.04 | 0.00 | 0.32 | 1.82 | 0.60 | 29.10 |
| Norway | 1991 | 332.63 | 2.59 | 504.22 | 958.50 | 203.66 | 128.97 | 0.01 | 0.35 | 1.90 | 0.63 | 32.86 |
| Norway | 1992 | 373.53 | 2.96 | 510.48 | 1006.36 | 223.54 | 149.99 | 0.01 | 0.37 | 1.97 | 0.67 | 36.53 |
| Norway | 1993 | 406.31 | 3.23 | 516.34 | 1051.21 | 241.85 | 164.45 | 0.01 | 0.39 | 2.04 | 0.68 | 38.70 |
| Norway | 1994 | 445.48 | 3.68 | 521.62 | 1092.46 | 258.68 | 186.81 | 0.01 | 0.41 | 2.09 | 0.72 | 42.49 |
| Norway | 1995 | 484.65 | 4.08 | 526.14 | 1129.53 | 273.46 | 211.20 | 0.01 | 0.43 | 2.15 | 0.77 | 46.24 |
| Norway | 1996 | 527.13 | 4.57 | 530.46 | 1164.91 | 287.58 | 239.55 | 0.01 | 0.45 | 2.20 | 0.83 | 50.64 |
| Norway | 1997 | 568.33 | 5.09 | 534.97 | 1199.72 | 301.70 | 266.63 | 0.01 | 0.47 | 2.24 | 0.88 | 54.79 |
| Norway | 1998 | 625.16 | 5.88 | 539.42 | 1232.15 | 315.09 | 310.07 | 0.01 | 0.51 | 2.28 | 0.98 | 61.65 |
| Norway | 1999 | 659.10 | 6.29 | 543.46 | 1259.94 | 325.93 | 333.17 | 0.01 | 0.52 | 2.32 | 1.02 | 64.73 |
| Norway | 2000 | 717.89 | 7.24 | 546.78 | 1280.99 | 333.07 | 384.82 | 0.01 | 0.56 | 2.34 | 1.16 | 72.95 |
| Norway | 2001 | 741.54 | 7.59 | 557.81 | 1298.85 | 338.64 | 402.90 | 0.01 | 0.57 | 2.33 | 1.19 | 74.98 |
| Norway | 2002 | 723.94 | 7.24 | 580.09 | 1316.70 | 343.50 | 380.45 | 0.01 | 0.55 | 2.27 | 1.11 | 69.85 |
| Norway | 2003 | 696.63 | 6.71 | 605.96 | 1332.57 | 347.66 | 348.97 | 0.01 | 0.52 | 2.20 | 1.00 | 63.38 |
| Norway | 2004 | 693.43 | 6.62 | 627.86 | 1344.55 | 350.28 | 343.15 | 0.01 | 0.52 | 2.14 | 0.98 | 61.39 |
| Norway | 2005 | 681.05 | 6.41 | 638.24 | 1350.98 | 351.13 | 329.93 | 0.01 | 0.50 | 2.12 | 0.94 | 58.91 |
| Norway | 2006 | 666.81 | 6.23 | 639.29 | 1343.72 | 346.40 | 320.40 | 0.01 | 0.50 | 2.10 | 0.92 | 57.50 |
| Norway | 2007 | 648.58 | 6.12 | 638.82 | 1321.24 | 334.99 | 313.59 | 0.01 | 0.49 | 2.07 | 0.94 | 56.97 |
| Norway | 2008 | 634.26 | 6.16 | 637.50 | 1291.12 | 320.68 | 313.58 | 0.01 | 0.49 | 2.03 | 0.98 | 57.83 |
| Norway | 2009 | 616.97 | 6.11 | 636.02 | 1261.14 | 306.68 | 310.30 | 0.01 | 0.49 | 1.98 | 1.01 | 58.12 |
| Norway | 2010 | 597.33 | 5.93 | 635.19 | 1239.76 | 296.73 | 300.60 | 0.01 | 0.48 | 1.95 | 1.01 | 57.05 |
| Norway | 2011 | 579.94 | 5.73 | 631.30 | 1224.75 | 289.81 | 290.13 | 0.01 | 0.47 | 1.94 | 1.00 | 55.75 |
| Norway | 2012 | 557.17 | 5.49 | 621.70 | 1209.33 | 283.58 | 273.59 | 0.01 | 0.46 | 1.95 | 0.96 | 53.77 |
| Norway | 2013 | 546.81 | 5.43 | 608.73 | 1194.53 | 277.86 | 268.95 | 0.01 | 0.46 | 1.96 | 0.97 | 53.79 |
| Norway | 2014 | 534.97 | 5.29 | 594.68 | 1181.31 | 273.21 | 261.76 | 0.01 | 0.45 | 1.99 | 0.96 | 53.25 |
| Norway | 2015 | 520.23 | 5.09 | 581.91 | 1171.18 | 269.61 | 250.62 | 0.01 | 0.44 | 2.01 | 0.93 | 51.89 |
| Norway | 2016 | 507.63 | 4.91 | 569.40 | 1161.92 | 266.50 | 241.13 | 0.01 | 0.44 | 2.04 | 0.90 | 50.73 |
| Norway | 2017 | 492.45 | 4.67 | 556.53 | 1152.20 | 263.57 | 228.88 | 0.01 | 0.43 | 2.07 | 0.87 | 48.99 |
| Norway | 2018 | 489.24 | 4.65 | 546.27 | 1144.37 | 261.35 | 227.89 | 0.01 | 0.43 | 2.09 | 0.87 | 49.30 |
| Norway | 2019 | 484.39 | 4.58 | 541.63 | 1140.68 | 260.01 | 224.38 | 0.01 | 0.42 | 2.11 | 0.86 | 48.85 |
| Norway | 2020 | 483.47 | 4.52 | 533.54 | 1149.81 | 261.00 | 222.47 | 0.01 | 0.42 | 2.16 | 0.85 | 48.43 |
| Norway | 2021 | 472.16 | 4.27 | 536.13 | 1157.34 | 264.23 | 207.93 | 0.01 | 0.41 | 2.16 | 0.79 | 45.45 |
| Poland | 1990 | 133.22 | 1.24 | 432.31 | 828.04 | 79.78 | 53.44 | 0.00 | 0.16 | 1.92 | 0.67 | 15.77 |
| Poland | 1991 | 135.68 | 1.31 | 436.64 | 827.54 | 79.84 | 55.84 | 0.00 | 0.16 | 1.90 | 0.70 | 16.73 |
| Poland | 1992 | 133.80 | 1.28 | 440.34 | 826.75 | 79.94 | 53.86 | 0.00 | 0.16 | 1.88 | 0.67 | 15.97 |
| Poland | 1993 | 130.80 | 1.20 | 443.39 | 825.85 | 79.97 | 50.83 | 0.00 | 0.16 | 1.86 | 0.64 | 14.73 |
| Poland | 1994 | 131.13 | 1.20 | 445.77 | 825.00 | 79.98 | 51.15 | 0.00 | 0.16 | 1.85 | 0.64 | 14.80 |
| Poland | 1995 | 130.53 | 1.18 | 447.48 | 824.35 | 80.08 | 50.45 | 0.00 | 0.16 | 1.84 | 0.63 | 14.50 |
| Poland | 1996 | 129.28 | 1.16 | 447.79 | 824.40 | 80.11 | 49.17 | 0.00 | 0.16 | 1.84 | 0.61 | 14.04 |
| Poland | 1997 | 131.40 | 1.23 | 446.61 | 825.14 | 80.31 | 51.09 | 0.00 | 0.16 | 1.85 | 0.64 | 14.90 |
| Poland | 1998 | 138.12 | 1.47 | 444.98 | 825.89 | 80.29 | 57.83 | 0.00 | 0.17 | 1.86 | 0.72 | 17.96 |
| Poland | 1999 | 141.23 | 1.60 | 443.92 | 825.98 | 80.09 | 61.14 | 0.00 | 0.17 | 1.86 | 0.76 | 19.50 |
| Poland | 2000 | 136.74 | 1.51 | 444.49 | 824.76 | 79.31 | 57.43 | 0.00 | 0.17 | 1.86 | 0.72 | 18.12 |
| Poland | 2001 | 133.73 | 1.50 | 450.11 | 820.59 | 77.70 | 56.03 | 0.00 | 0.16 | 1.82 | 0.72 | 17.72 |
| Poland | 2002 | 131.14 | 1.51 | 461.21 | 813.63 | 75.39 | 55.75 | 0.00 | 0.16 | 1.76 | 0.74 | 17.77 |
| Poland | 2003 | 126.23 | 1.44 | 474.36 | 806.00 | 72.94 | 53.29 | 0.00 | 0.16 | 1.70 | 0.73 | 16.85 |
| Poland | 2004 | 122.40 | 1.40 | 486.15 | 799.79 | 70.96 | 51.45 | 0.00 | 0.15 | 1.65 | 0.73 | 16.20 |
| Poland | 2005 | 121.36 | 1.43 | 493.18 | 797.13 | 70.08 | 51.28 | 0.00 | 0.15 | 1.62 | 0.73 | 16.27 |
| Poland | 2006 | 119.43 | 1.38 | 496.65 | 796.96 | 69.98 | 49.44 | 0.00 | 0.15 | 1.60 | 0.71 | 15.48 |
| Poland | 2007 | 118.33 | 1.37 | 499.88 | 796.87 | 69.84 | 48.49 | 0.00 | 0.15 | 1.59 | 0.69 | 15.09 |
| Poland | 2008 | 119.70 | 1.43 | 502.70 | 796.97 | 69.74 | 49.95 | 0.00 | 0.15 | 1.59 | 0.72 | 15.79 |
| Poland | 2009 | 120.63 | 1.46 | 504.97 | 797.35 | 69.75 | 50.88 | 0.00 | 0.15 | 1.58 | 0.73 | 16.17 |
| Poland | 2010 | 120.31 | 1.45 | 506.55 | 798.11 | 69.86 | 50.46 | 0.00 | 0.15 | 1.58 | 0.72 | 15.96 |
| Poland | 2011 | 118.02 | 1.36 | 507.66 | 801.55 | 70.30 | 47.72 | 0.00 | 0.15 | 1.58 | 0.68 | 14.63 |
| Poland | 2012 | 119.84 | 1.37 | 508.44 | 808.37 | 71.51 | 48.33 | 0.00 | 0.15 | 1.59 | 0.68 | 14.71 |
| Poland | 2013 | 120.12 | 1.33 | 508.63 | 816.35 | 72.86 | 47.26 | 0.00 | 0.15 | 1.61 | 0.65 | 14.03 |
| Poland | 2014 | 122.42 | 1.35 | 508.00 | 823.25 | 74.21 | 48.20 | 0.00 | 0.15 | 1.62 | 0.65 | 14.23 |
| Poland | 2015 | 124.51 | 1.37 | 506.30 | 826.80 | 75.24 | 49.27 | 0.00 | 0.15 | 1.63 | 0.65 | 14.56 |
| Poland | 2016 | 126.21 | 1.41 | 501.00 | 819.74 | 75.64 | 50.58 | 0.00 | 0.15 | 1.64 | 0.67 | 15.23 |
| Poland | 2017 | 129.10 | 1.46 | 492.26 | 802.98 | 75.87 | 53.22 | 0.00 | 0.16 | 1.63 | 0.70 | 16.53 |
| Poland | 2018 | 134.78 | 1.56 | 483.93 | 786.61 | 76.10 | 58.68 | 0.00 | 0.17 | 1.63 | 0.77 | 18.88 |
| Poland | 2019 | 139.35 | 1.62 | 479.83 | 780.75 | 76.94 | 62.41 | 0.00 | 0.18 | 1.63 | 0.81 | 20.32 |
| Poland | 2020 | 140.59 | 1.59 | 481.32 | 793.37 | 79.33 | 61.27 | 0.00 | 0.18 | 1.65 | 0.77 | 19.50 |
| Poland | 2021 | 142.48 | 1.61 | 483.21 | 795.65 | 80.26 | 62.22 | 0.00 | 0.18 | 1.65 | 0.78 | 19.72 |
| Portugal | 1990 | 182.54 | 2.29 | 392.03 | 805.29 | 94.55 | 88.00 | 0.01 | 0.23 | 2.05 | 0.93 | 30.73 |
| Portugal | 1991 | 193.61 | 2.49 | 394.18 | 799.19 | 94.83 | 98.79 | 0.01 | 0.24 | 2.03 | 1.04 | 34.50 |
| Portugal | 1992 | 202.29 | 2.64 | 396.07 | 792.99 | 94.60 | 107.68 | 0.01 | 0.26 | 2.00 | 1.14 | 37.68 |
| Portugal | 1993 | 209.06 | 2.82 | 397.52 | 786.92 | 94.74 | 114.31 | 0.01 | 0.27 | 1.98 | 1.21 | 40.39 |
| Portugal | 1994 | 212.97 | 2.92 | 398.40 | 781.18 | 94.75 | 118.21 | 0.01 | 0.27 | 1.96 | 1.25 | 42.01 |
| Portugal | 1995 | 217.80 | 3.01 | 398.55 | 775.97 | 94.95 | 122.85 | 0.01 | 0.28 | 1.95 | 1.29 | 43.74 |
| Portugal | 1996 | 220.11 | 3.11 | 398.04 | 768.83 | 94.90 | 125.20 | 0.01 | 0.29 | 1.93 | 1.32 | 45.12 |
| Portugal | 1997 | 220.66 | 3.15 | 397.21 | 758.71 | 94.86 | 125.80 | 0.01 | 0.29 | 1.91 | 1.33 | 45.78 |
| Portugal | 1998 | 219.56 | 3.21 | 396.24 | 747.97 | 94.57 | 124.98 | 0.01 | 0.29 | 1.89 | 1.32 | 46.25 |
| Portugal | 1999 | 214.78 | 3.12 | 395.32 | 738.92 | 94.70 | 120.07 | 0.01 | 0.29 | 1.87 | 1.27 | 44.77 |
| Portugal | 2000 | 205.25 | 2.87 | 394.64 | 733.88 | 94.47 | 110.78 | 0.01 | 0.28 | 1.86 | 1.17 | 41.21 |
| Portugal | 2001 | 192.50 | 2.47 | 393.31 | 730.98 | 94.10 | 98.40 | 0.01 | 0.26 | 1.86 | 1.05 | 35.97 |
| Portugal | 2002 | 178.32 | 2.06 | 390.77 | 727.14 | 93.30 | 85.02 | 0.01 | 0.25 | 1.86 | 0.91 | 30.48 |
| Portugal | 2003 | 170.13 | 1.90 | 387.72 | 722.96 | 92.67 | 77.47 | 0.00 | 0.24 | 1.86 | 0.84 | 27.83 |
| Portugal | 2004 | 164.66 | 1.80 | 384.86 | 718.97 | 91.78 | 72.88 | 0.00 | 0.23 | 1.87 | 0.79 | 26.28 |
| Portugal | 2005 | 157.78 | 1.65 | 382.88 | 715.75 | 91.46 | 66.33 | 0.00 | 0.22 | 1.87 | 0.73 | 23.82 |
| Portugal | 2006 | 156.04 | 1.65 | 382.19 | 712.34 | 91.50 | 64.55 | 0.00 | 0.22 | 1.86 | 0.71 | 23.47 |
| Portugal | 2007 | 164.11 | 1.95 | 382.45 | 708.10 | 91.29 | 72.82 | 0.01 | 0.23 | 1.85 | 0.80 | 27.42 |
| Portugal | 2008 | 167.12 | 2.10 | 383.26 | 703.91 | 91.08 | 76.04 | 0.01 | 0.24 | 1.84 | 0.83 | 29.22 |
| Portugal | 2009 | 167.73 | 2.10 | 384.23 | 700.73 | 91.12 | 76.61 | 0.01 | 0.24 | 1.82 | 0.84 | 29.44 |
| Portugal | 2010 | 166.80 | 2.11 | 384.95 | 699.53 | 90.88 | 75.91 | 0.01 | 0.24 | 1.82 | 0.84 | 29.30 |
| Portugal | 2011 | 163.86 | 2.01 | 386.10 | 703.29 | 91.47 | 72.39 | 0.01 | 0.23 | 1.82 | 0.79 | 27.65 |
| Portugal | 2012 | 162.93 | 1.95 | 388.20 | 712.12 | 92.44 | 70.50 | 0.01 | 0.23 | 1.83 | 0.76 | 26.53 |
| Portugal | 2013 | 163.47 | 1.95 | 390.64 | 722.65 | 93.22 | 70.25 | 0.00 | 0.23 | 1.85 | 0.75 | 26.15 |
| Portugal | 2014 | 164.93 | 2.01 | 392.78 | 731.51 | 93.85 | 71.08 | 0.01 | 0.23 | 1.86 | 0.76 | 26.38 |
| Portugal | 2015 | 165.25 | 2.03 | 394.03 | 735.39 | 93.78 | 71.47 | 0.01 | 0.22 | 1.87 | 0.76 | 26.52 |
| Portugal | 2016 | 167.08 | 2.13 | 393.72 | 728.04 | 92.96 | 74.12 | 0.01 | 0.23 | 1.85 | 0.80 | 27.94 |
| Portugal | 2017 | 171.04 | 2.26 | 392.26 | 711.50 | 91.81 | 79.22 | 0.01 | 0.24 | 1.81 | 0.86 | 30.45 |
| Portugal | 2018 | 175.19 | 2.39 | 390.64 | 694.90 | 90.61 | 84.58 | 0.01 | 0.25 | 1.78 | 0.93 | 33.14 |
| Portugal | 2019 | 174.65 | 2.37 | 389.82 | 687.32 | 89.90 | 84.74 | 0.01 | 0.25 | 1.76 | 0.94 | 33.32 |
| Portugal | 2020 | 170.83 | 2.27 | 386.23 | 687.20 | 89.55 | 81.29 | 0.01 | 0.25 | 1.78 | 0.91 | 32.01 |
| Portugal | 2021 | 169.73 | 2.23 | 385.97 | 688.92 | 89.89 | 79.84 | 0.01 | 0.25 | 1.78 | 0.89 | 31.34 |
| Republic of Moldova | 1990 | 195.14 | 1.45 | 338.20 | 654.20 | 128.38 | 66.76 | 0.00 | 0.30 | 1.93 | 0.52 | 25.94 |
| Republic of Moldova | 1991 | 197.94 | 1.52 | 336.82 | 652.86 | 127.95 | 69.99 | 0.00 | 0.30 | 1.94 | 0.55 | 27.15 |
| Republic of Moldova | 1992 | 195.69 | 1.48 | 335.16 | 651.62 | 127.39 | 68.30 | 0.00 | 0.30 | 1.94 | 0.54 | 26.57 |
| Republic of Moldova | 1993 | 194.69 | 1.47 | 333.26 | 650.62 | 127.18 | 67.51 | 0.00 | 0.30 | 1.95 | 0.53 | 26.40 |
| Republic of Moldova | 1994 | 199.28 | 1.59 | 331.21 | 649.87 | 126.80 | 72.48 | 0.00 | 0.31 | 1.96 | 0.57 | 28.38 |
| Republic of Moldova | 1995 | 200.98 | 1.62 | 329.08 | 649.42 | 126.90 | 74.08 | 0.00 | 0.31 | 1.97 | 0.58 | 29.02 |
| Republic of Moldova | 1996 | 198.51 | 1.57 | 325.84 | 646.65 | 126.72 | 71.79 | 0.00 | 0.31 | 1.98 | 0.57 | 28.37 |
| Republic of Moldova | 1997 | 194.09 | 1.47 | 321.14 | 640.41 | 126.60 | 67.49 | 0.00 | 0.30 | 1.99 | 0.53 | 27.04 |
| Republic of Moldova | 1998 | 192.83 | 1.43 | 316.04 | 633.08 | 126.36 | 66.47 | 0.00 | 0.30 | 2.00 | 0.53 | 26.92 |
| Republic of Moldova | 1999 | 196.88 | 1.50 | 311.65 | 626.96 | 126.57 | 70.32 | 0.00 | 0.31 | 2.01 | 0.56 | 28.70 |
| Republic of Moldova | 2000 | 194.94 | 1.47 | 309.05 | 624.44 | 126.61 | 68.33 | 0.00 | 0.31 | 2.02 | 0.54 | 28.16 |
| Republic of Moldova | 2001 | 191.76 | 1.40 | 307.56 | 624.47 | 126.63 | 65.13 | 0.00 | 0.31 | 2.03 | 0.51 | 27.01 |
| Republic of Moldova | 2002 | 190.39 | 1.39 | 305.96 | 624.44 | 126.49 | 63.90 | 0.00 | 0.30 | 2.04 | 0.51 | 26.67 |
| Republic of Moldova | 2003 | 190.39 | 1.39 | 304.51 | 624.41 | 126.82 | 63.57 | 0.00 | 0.30 | 2.05 | 0.50 | 26.69 |
| Republic of Moldova | 2004 | 192.70 | 1.44 | 303.48 | 624.43 | 126.77 | 65.93 | 0.00 | 0.31 | 2.06 | 0.52 | 27.60 |
| Republic of Moldova | 2005 | 201.08 | 1.62 | 303.15 | 624.56 | 126.58 | 74.50 | 0.01 | 0.32 | 2.06 | 0.59 | 30.88 |
| Republic of Moldova | 2006 | 203.88 | 1.68 | 304.09 | 625.06 | 126.81 | 77.07 | 0.01 | 0.33 | 2.06 | 0.61 | 31.82 |
| Republic of Moldova | 2007 | 203.32 | 1.66 | 306.18 | 625.88 | 127.03 | 76.29 | 0.01 | 0.32 | 2.04 | 0.60 | 31.38 |
| Republic of Moldova | 2008 | 198.34 | 1.56 | 308.66 | 626.71 | 126.89 | 71.45 | 0.01 | 0.32 | 2.03 | 0.56 | 29.46 |
| Republic of Moldova | 2009 | 194.30 | 1.47 | 310.80 | 627.23 | 127.37 | 66.93 | 0.00 | 0.31 | 2.02 | 0.53 | 27.71 |
| Republic of Moldova | 2010 | 193.60 | 1.46 | 311.84 | 627.10 | 127.06 | 66.54 | 0.00 | 0.31 | 2.01 | 0.52 | 27.53 |
| Republic of Moldova | 2011 | 183.44 | 1.26 | 311.90 | 625.06 | 126.66 | 56.77 | 0.00 | 0.29 | 2.00 | 0.45 | 23.94 |
| Republic of Moldova | 2012 | 179.99 | 1.23 | 311.64 | 620.67 | 124.87 | 55.12 | 0.00 | 0.29 | 1.99 | 0.44 | 23.39 |
| Republic of Moldova | 2013 | 175.87 | 1.18 | 311.10 | 614.87 | 122.76 | 53.11 | 0.00 | 0.29 | 1.98 | 0.43 | 22.67 |
| Republic of Moldova | 2014 | 177.17 | 1.26 | 310.36 | 608.66 | 120.90 | 56.27 | 0.00 | 0.29 | 1.96 | 0.47 | 24.08 |
| Republic of Moldova | 2015 | 176.26 | 1.29 | 309.48 | 602.98 | 118.83 | 57.43 | 0.00 | 0.29 | 1.95 | 0.48 | 24.66 |
| Republic of Moldova | 2016 | 172.70 | 1.26 | 308.05 | 596.54 | 116.82 | 55.88 | 0.00 | 0.29 | 1.94 | 0.48 | 24.25 |
| Republic of Moldova | 2017 | 167.10 | 1.21 | 306.05 | 588.90 | 113.71 | 53.39 | 0.00 | 0.28 | 1.92 | 0.47 | 23.41 |
| Republic of Moldova | 2018 | 166.23 | 1.23 | 304.22 | 582.30 | 111.62 | 54.62 | 0.00 | 0.29 | 1.91 | 0.49 | 24.05 |
| Republic of Moldova | 2019 | 169.43 | 1.31 | 303.26 | 578.99 | 110.60 | 58.82 | 0.00 | 0.29 | 1.91 | 0.53 | 25.77 |
| Republic of Moldova | 2020 | 167.28 | 1.28 | 305.18 | 581.25 | 110.65 | 56.63 | 0.00 | 0.29 | 1.90 | 0.51 | 24.87 |
| Republic of Moldova | 2021 | 170.93 | 1.36 | 305.56 | 580.86 | 110.38 | 60.55 | 0.00 | 0.29 | 1.90 | 0.55 | 26.43 |
| Romania | 1990 | 105.97 | 1.30 | 320.24 | 330.38 | 46.66 | 59.30 | 0.00 | 0.32 | 1.03 | 1.27 | 38.87 |
| Romania | 1991 | 105.83 | 1.30 | 319.82 | 331.01 | 47.39 | 58.44 | 0.00 | 0.32 | 1.04 | 1.23 | 38.23 |
| Romania | 1992 | 107.12 | 1.32 | 319.47 | 331.33 | 48.24 | 58.87 | 0.00 | 0.32 | 1.04 | 1.22 | 38.41 |
| Romania | 1993 | 109.52 | 1.37 | 319.20 | 331.36 | 48.70 | 60.82 | 0.00 | 0.33 | 1.04 | 1.25 | 39.72 |
| Romania | 1994 | 112.83 | 1.44 | 319.02 | 331.21 | 49.06 | 63.77 | 0.00 | 0.34 | 1.04 | 1.30 | 41.67 |
| Romania | 1995 | 114.23 | 1.46 | 318.93 | 330.93 | 49.36 | 64.88 | 0.00 | 0.35 | 1.04 | 1.31 | 42.37 |
| Romania | 1996 | 115.80 | 1.50 | 318.98 | 330.16 | 49.35 | 66.45 | 0.00 | 0.35 | 1.04 | 1.35 | 43.48 |
| Romania | 1997 | 114.84 | 1.49 | 319.15 | 328.68 | 48.98 | 65.86 | 0.00 | 0.35 | 1.03 | 1.34 | 43.28 |
| Romania | 1998 | 111.54 | 1.42 | 319.36 | 326.77 | 48.48 | 63.06 | 0.00 | 0.34 | 1.02 | 1.30 | 41.59 |
| Romania | 1999 | 108.54 | 1.35 | 319.54 | 324.75 | 47.87 | 60.68 | 0.00 | 0.33 | 1.02 | 1.27 | 40.10 |
| Romania | 2000 | 104.66 | 1.27 | 319.64 | 322.92 | 47.07 | 57.59 | 0.00 | 0.32 | 1.01 | 1.22 | 38.22 |
| Romania | 2001 | 103.86 | 1.29 | 319.16 | 320.80 | 46.19 | 57.67 | 0.00 | 0.32 | 1.01 | 1.25 | 38.69 |
| Romania | 2002 | 101.69 | 1.27 | 318.04 | 318.10 | 45.10 | 56.59 | 0.00 | 0.32 | 1.00 | 1.25 | 38.43 |
| Romania | 2003 | 98.92 | 1.25 | 316.82 | 315.38 | 43.58 | 55.34 | 0.00 | 0.31 | 1.00 | 1.27 | 38.14 |
| Romania | 2004 | 96.19 | 1.22 | 316.04 | 313.22 | 42.59 | 53.60 | 0.00 | 0.31 | 0.99 | 1.26 | 37.32 |
| Romania | 2005 | 94.15 | 1.19 | 316.26 | 312.18 | 41.98 | 52.17 | 0.00 | 0.30 | 0.99 | 1.24 | 36.52 |
| Romania | 2006 | 92.10 | 1.15 | 318.01 | 312.27 | 41.86 | 50.24 | 0.00 | 0.29 | 0.98 | 1.20 | 35.06 |
| Romania | 2007 | 91.42 | 1.13 | 320.90 | 312.97 | 41.90 | 49.52 | 0.00 | 0.29 | 0.98 | 1.18 | 34.38 |
| Romania | 2008 | 94.01 | 1.19 | 324.12 | 314.06 | 42.05 | 51.96 | 0.00 | 0.30 | 0.97 | 1.24 | 36.01 |
| Romania | 2009 | 95.53 | 1.22 | 326.85 | 315.37 | 42.35 | 53.17 | 0.00 | 0.30 | 0.96 | 1.26 | 36.73 |
| Romania | 2010 | 95.20 | 1.21 | 328.28 | 316.69 | 42.97 | 52.22 | 0.00 | 0.30 | 0.96 | 1.22 | 35.83 |
| Romania | 2011 | 90.88 | 1.10 | 328.49 | 318.20 | 43.48 | 47.40 | 0.00 | 0.29 | 0.97 | 1.09 | 32.15 |
| Romania | 2012 | 93.09 | 1.14 | 328.29 | 320.14 | 44.08 | 49.01 | 0.00 | 0.29 | 0.98 | 1.11 | 33.12 |
| Romania | 2013 | 91.47 | 1.08 | 327.92 | 322.36 | 44.88 | 46.59 | 0.00 | 0.28 | 0.98 | 1.04 | 31.10 |
| Romania | 2014 | 92.42 | 1.09 | 327.63 | 324.63 | 45.53 | 46.89 | 0.00 | 0.28 | 0.99 | 1.03 | 31.06 |
| Romania | 2015 | 92.96 | 1.08 | 327.66 | 326.78 | 46.36 | 46.60 | 0.00 | 0.28 | 1.00 | 1.01 | 30.65 |
| Romania | 2016 | 92.27 | 1.05 | 328.92 | 329.50 | 47.36 | 44.91 | 0.00 | 0.28 | 1.00 | 0.95 | 29.22 |
| Romania | 2017 | 94.87 | 1.09 | 331.44 | 332.97 | 48.50 | 46.37 | 0.00 | 0.28 | 1.00 | 0.96 | 29.85 |
| Romania | 2018 | 98.39 | 1.14 | 334.15 | 336.15 | 49.71 | 48.68 | 0.00 | 0.29 | 1.01 | 0.98 | 31.03 |
| Romania | 2019 | 100.62 | 1.17 | 335.97 | 338.06 | 50.37 | 50.25 | 0.00 | 0.30 | 1.01 | 1.00 | 31.79 |
| Romania | 2020 | 98.51 | 1.15 | 338.15 | 337.86 | 49.54 | 48.97 | 0.00 | 0.29 | 1.00 | 0.99 | 31.05 |
| Romania | 2021 | 100.65 | 1.20 | 338.79 | 335.90 | 49.42 | 51.23 | 0.00 | 0.30 | 0.99 | 1.04 | 32.66 |
| Russian Federation | 1990 | 410.96 | 3.64 | 443.42 | 1097.37 | 240.27 | 170.69 | 0.01 | 0.37 | 2.47 | 0.71 | 42.40 |
| Russian Federation | 1991 | 437.42 | 3.84 | 454.74 | 1150.98 | 256.02 | 181.39 | 0.01 | 0.38 | 2.53 | 0.71 | 43.28 |
| Russian Federation | 1992 | 481.37 | 4.40 | 465.53 | 1203.87 | 271.82 | 209.55 | 0.01 | 0.40 | 2.59 | 0.77 | 47.75 |
| Russian Federation | 1993 | 544.46 | 5.33 | 475.54 | 1254.46 | 287.67 | 256.78 | 0.01 | 0.43 | 2.64 | 0.89 | 55.72 |
| Russian Federation | 1994 | 589.72 | 5.88 | 484.48 | 1301.28 | 303.19 | 286.53 | 0.01 | 0.45 | 2.69 | 0.95 | 59.84 |
| Russian Federation | 1995 | 600.42 | 5.71 | 492.05 | 1343.00 | 318.24 | 282.18 | 0.01 | 0.45 | 2.73 | 0.89 | 57.37 |
| Russian Federation | 1996 | 609.67 | 5.48 | 499.22 | 1387.15 | 335.19 | 274.48 | 0.01 | 0.44 | 2.78 | 0.82 | 54.43 |
| Russian Federation | 1997 | 620.18 | 5.25 | 506.60 | 1437.13 | 354.47 | 265.71 | 0.01 | 0.43 | 2.84 | 0.75 | 51.47 |
| Russian Federation | 1998 | 650.29 | 5.39 | 513.41 | 1485.34 | 372.98 | 277.31 | 0.01 | 0.44 | 2.89 | 0.74 | 52.11 |
| Russian Federation | 1999 | 711.06 | 6.22 | 518.83 | 1524.22 | 387.97 | 323.09 | 0.01 | 0.47 | 2.94 | 0.83 | 58.63 |
| Russian Federation | 2000 | 749.25 | 6.73 | 522.09 | 1546.31 | 396.47 | 352.78 | 0.01 | 0.48 | 2.96 | 0.89 | 62.70 |
| Russian Federation | 2001 | 740.23 | 6.53 | 524.37 | 1555.92 | 400.35 | 339.88 | 0.01 | 0.48 | 2.97 | 0.85 | 60.52 |
| Russian Federation | 2002 | 732.04 | 6.40 | 527.10 | 1562.22 | 402.89 | 329.15 | 0.01 | 0.47 | 2.96 | 0.82 | 58.86 |
| Russian Federation | 2003 | 759.29 | 6.85 | 529.96 | 1565.19 | 404.35 | 354.93 | 0.01 | 0.49 | 2.95 | 0.88 | 62.64 |
| Russian Federation | 2004 | 803.21 | 7.57 | 532.57 | 1564.83 | 404.43 | 398.77 | 0.01 | 0.51 | 2.94 | 0.99 | 69.01 |
| Russian Federation | 2005 | 893.43 | 9.13 | 534.60 | 1561.26 | 403.16 | 490.27 | 0.02 | 0.57 | 2.92 | 1.22 | 82.80 |
| Russian Federation | 2006 | 900.09 | 9.27 | 535.64 | 1551.05 | 399.09 | 501.00 | 0.02 | 0.58 | 2.90 | 1.26 | 84.45 |
| Russian Federation | 2007 | 863.43 | 8.77 | 535.50 | 1532.47 | 391.50 | 471.93 | 0.02 | 0.56 | 2.86 | 1.21 | 80.52 |
| Russian Federation | 2008 | 819.05 | 8.21 | 534.23 | 1507.95 | 381.56 | 437.49 | 0.02 | 0.54 | 2.82 | 1.15 | 76.06 |
| Russian Federation | 2009 | 731.35 | 6.90 | 531.84 | 1480.08 | 370.10 | 361.25 | 0.01 | 0.49 | 2.78 | 0.98 | 64.92 |
| Russian Federation | 2010 | 692.76 | 6.46 | 528.33 | 1451.49 | 358.58 | 334.18 | 0.01 | 0.48 | 2.75 | 0.93 | 61.49 |
| Russian Federation | 2011 | 642.32 | 5.80 | 523.38 | 1414.36 | 343.96 | 298.36 | 0.01 | 0.45 | 2.70 | 0.87 | 56.45 |
| Russian Federation | 2012 | 617.09 | 5.68 | 517.28 | 1365.03 | 324.88 | 292.21 | 0.01 | 0.45 | 2.64 | 0.90 | 56.59 |
| Russian Federation | 2013 | 596.21 | 5.69 | 510.91 | 1312.28 | 304.53 | 291.69 | 0.01 | 0.45 | 2.57 | 0.96 | 58.03 |
| Russian Federation | 2014 | 583.18 | 5.84 | 505.08 | 1264.90 | 286.23 | 296.95 | 0.01 | 0.46 | 2.50 | 1.04 | 60.64 |
| Russian Federation | 2015 | 543.97 | 5.43 | 500.61 | 1231.77 | 273.35 | 270.62 | 0.01 | 0.44 | 2.46 | 0.99 | 57.04 |
| Russian Federation | 2016 | 519.84 | 5.19 | 496.37 | 1206.30 | 264.24 | 255.60 | 0.01 | 0.43 | 2.43 | 0.97 | 55.11 |
| Russian Federation | 2017 | 492.78 | 4.83 | 491.54 | 1180.11 | 255.81 | 236.97 | 0.01 | 0.42 | 2.40 | 0.93 | 52.24 |
| Russian Federation | 2018 | 481.53 | 4.77 | 487.33 | 1159.06 | 249.54 | 232.00 | 0.01 | 0.42 | 2.38 | 0.93 | 52.05 |
| Russian Federation | 2019 | 477.02 | 4.74 | 484.92 | 1149.06 | 246.45 | 230.57 | 0.01 | 0.42 | 2.37 | 0.94 | 52.09 |
| Russian Federation | 2020 | 473.71 | 4.68 | 484.09 | 1150.66 | 246.15 | 227.56 | 0.01 | 0.41 | 2.38 | 0.92 | 51.45 |
| Russian Federation | 2021 | 482.04 | 4.85 | 484.94 | 1149.34 | 245.07 | 236.97 | 0.01 | 0.42 | 2.37 | 0.97 | 53.34 |
| San Marino | 1990 | 152.33 | 1.47 | 453.79 | 902.67 | 113.21 | 39.12 | 0.00 | 0.17 | 1.99 | 0.35 | 12.04 |
| San Marino | 1991 | 151.80 | 1.44 | 452.52 | 903.57 | 113.55 | 38.25 | 0.00 | 0.17 | 2.00 | 0.34 | 11.75 |
| San Marino | 1992 | 151.61 | 1.42 | 451.53 | 904.49 | 114.09 | 37.52 | 0.00 | 0.17 | 2.00 | 0.33 | 11.52 |
| San Marino | 1993 | 150.96 | 1.39 | 450.85 | 905.40 | 114.38 | 36.58 | 0.00 | 0.17 | 2.01 | 0.32 | 11.18 |
| San Marino | 1994 | 150.84 | 1.38 | 450.55 | 906.27 | 114.77 | 36.06 | 0.00 | 0.17 | 2.01 | 0.31 | 10.99 |
| San Marino | 1995 | 150.06 | 1.34 | 450.67 | 907.07 | 115.02 | 35.04 | 0.00 | 0.17 | 2.01 | 0.30 | 10.60 |
| San Marino | 1996 | 149.37 | 1.31 | 451.34 | 907.99 | 115.21 | 34.15 | 0.00 | 0.16 | 2.01 | 0.30 | 10.25 |
| San Marino | 1997 | 148.69 | 1.27 | 452.53 | 909.19 | 115.67 | 33.03 | 0.00 | 0.16 | 2.01 | 0.29 | 9.81 |
| San Marino | 1998 | 147.98 | 1.22 | 454.01 | 910.63 | 115.94 | 32.04 | 0.00 | 0.16 | 2.01 | 0.28 | 9.37 |
| San Marino | 1999 | 147.15 | 1.17 | 455.54 | 912.22 | 116.49 | 30.66 | 0.00 | 0.16 | 2.00 | 0.26 | 8.81 |
| San Marino | 2000 | 146.35 | 1.12 | 456.89 | 913.91 | 116.96 | 29.40 | 0.00 | 0.16 | 2.00 | 0.25 | 8.28 |
| San Marino | 2001 | 145.90 | 1.08 | 458.34 | 916.03 | 117.52 | 28.38 | 0.00 | 0.16 | 2.00 | 0.24 | 7.86 |
| San Marino | 2002 | 145.58 | 1.04 | 460.18 | 918.76 | 118.19 | 27.40 | 0.00 | 0.16 | 2.00 | 0.23 | 7.45 |
| San Marino | 2003 | 145.90 | 1.02 | 462.09 | 921.73 | 118.83 | 27.06 | 0.00 | 0.16 | 1.99 | 0.23 | 7.27 |
| San Marino | 2004 | 145.82 | 0.94 | 463.78 | 924.61 | 119.73 | 26.09 | 0.00 | 0.16 | 1.99 | 0.22 | 6.69 |
| San Marino | 2005 | 146.33 | 0.93 | 464.95 | 927.04 | 120.53 | 25.80 | 0.00 | 0.16 | 1.99 | 0.21 | 6.57 |
| San Marino | 2006 | 146.94 | 0.92 | 465.92 | 929.43 | 121.36 | 25.58 | 0.00 | 0.16 | 1.99 | 0.21 | 6.51 |
| San Marino | 2007 | 147.30 | 0.92 | 467.07 | 932.12 | 121.91 | 25.39 | 0.00 | 0.16 | 2.00 | 0.21 | 6.44 |
| San Marino | 2008 | 147.90 | 0.91 | 468.21 | 934.70 | 122.72 | 25.17 | 0.00 | 0.16 | 2.00 | 0.21 | 6.37 |
| San Marino | 2009 | 148.68 | 0.91 | 469.14 | 936.73 | 123.53 | 25.15 | 0.00 | 0.16 | 2.00 | 0.20 | 6.37 |
| San Marino | 2010 | 148.93 | 0.91 | 469.67 | 937.79 | 123.82 | 25.11 | 0.00 | 0.16 | 2.00 | 0.20 | 6.37 |
| San Marino | 2011 | 148.55 | 0.91 | 469.79 | 937.69 | 123.56 | 24.98 | 0.00 | 0.16 | 2.00 | 0.20 | 6.32 |
| San Marino | 2012 | 148.13 | 0.91 | 469.70 | 936.75 | 123.21 | 24.92 | 0.00 | 0.16 | 1.99 | 0.20 | 6.30 |
| San Marino | 2013 | 147.28 | 0.91 | 469.53 | 935.28 | 122.32 | 24.97 | 0.00 | 0.16 | 1.99 | 0.20 | 6.29 |
| San Marino | 2014 | 146.72 | 0.93 | 469.42 | 933.60 | 121.54 | 25.19 | 0.00 | 0.16 | 1.99 | 0.21 | 6.39 |
| San Marino | 2015 | 145.96 | 0.93 | 469.49 | 932.03 | 120.83 | 25.13 | 0.00 | 0.16 | 1.99 | 0.21 | 6.37 |
| San Marino | 2016 | 145.29 | 0.94 | 469.97 | 929.89 | 120.03 | 25.27 | 0.00 | 0.16 | 1.98 | 0.21 | 6.41 |
| San Marino | 2017 | 144.17 | 0.94 | 470.77 | 926.94 | 118.86 | 25.31 | 0.00 | 0.16 | 1.97 | 0.21 | 6.41 |
| San Marino | 2018 | 142.51 | 0.93 | 471.55 | 924.15 | 117.53 | 24.98 | 0.00 | 0.15 | 1.96 | 0.21 | 6.24 |
| San Marino | 2019 | 141.36 | 0.91 | 471.94 | 922.49 | 116.76 | 24.60 | 0.00 | 0.15 | 1.95 | 0.21 | 6.07 |
| San Marino | 2020 | 134.65 | 0.64 | 470.17 | 922.90 | 116.47 | 18.18 | 0.00 | 0.15 | 1.96 | 0.16 | 3.50 |
| San Marino | 2021 | 133.79 | 0.60 | 472.27 | 924.01 | 116.78 | 17.01 | 0.00 | 0.14 | 1.96 | 0.15 | 3.08 |
| Serbia | 1990 | 115.11 | 1.38 | 393.11 | 485.76 | 58.86 | 56.25 | 0.00 | 0.24 | 1.24 | 0.96 | 26.96 |
| Serbia | 1991 | 114.93 | 1.37 | 391.51 | 485.93 | 58.95 | 55.97 | 0.00 | 0.24 | 1.24 | 0.95 | 26.83 |
| Serbia | 1992 | 113.77 | 1.35 | 390.21 | 486.95 | 59.14 | 54.63 | 0.00 | 0.23 | 1.25 | 0.92 | 26.16 |
| Serbia | 1993 | 113.64 | 1.36 | 389.30 | 488.82 | 59.34 | 54.30 | 0.00 | 0.23 | 1.26 | 0.92 | 26.00 |
| Serbia | 1994 | 114.62 | 1.39 | 388.60 | 490.18 | 59.61 | 55.00 | 0.00 | 0.23 | 1.26 | 0.92 | 26.39 |
| Serbia | 1995 | 115.67 | 1.41 | 388.19 | 491.07 | 60.21 | 55.45 | 0.00 | 0.24 | 1.27 | 0.92 | 26.61 |
| Serbia | 1996 | 116.28 | 1.41 | 387.67 | 489.15 | 61.18 | 55.10 | 0.00 | 0.24 | 1.26 | 0.90 | 26.47 |
| Serbia | 1997 | 117.44 | 1.40 | 386.67 | 483.13 | 62.88 | 54.55 | 0.00 | 0.24 | 1.25 | 0.87 | 26.29 |
| Serbia | 1998 | 114.45 | 1.27 | 385.60 | 475.99 | 64.86 | 49.58 | 0.00 | 0.24 | 1.23 | 0.76 | 23.80 |
| Serbia | 1999 | 119.19 | 1.34 | 384.89 | 470.66 | 66.72 | 52.46 | 0.00 | 0.25 | 1.22 | 0.79 | 25.39 |
| Serbia | 2000 | 122.82 | 1.37 | 384.95 | 470.04 | 68.43 | 54.39 | 0.00 | 0.26 | 1.22 | 0.79 | 26.25 |
| Serbia | 2001 | 124.65 | 1.36 | 385.22 | 472.71 | 69.59 | 55.07 | 0.00 | 0.26 | 1.23 | 0.79 | 26.32 |
| Serbia | 2002 | 125.84 | 1.35 | 384.98 | 474.75 | 70.53 | 55.31 | 0.00 | 0.27 | 1.23 | 0.78 | 26.24 |
| Serbia | 2003 | 130.89 | 1.41 | 384.61 | 476.30 | 71.57 | 59.32 | 0.00 | 0.27 | 1.24 | 0.83 | 27.95 |
| Serbia | 2004 | 134.54 | 1.46 | 384.62 | 478.03 | 72.46 | 62.08 | 0.00 | 0.28 | 1.24 | 0.86 | 29.12 |
| Serbia | 2005 | 138.55 | 1.52 | 385.32 | 479.73 | 73.05 | 65.49 | 0.00 | 0.29 | 1.25 | 0.90 | 30.58 |
| Serbia | 2006 | 139.90 | 1.53 | 387.10 | 481.92 | 73.95 | 65.95 | 0.00 | 0.29 | 1.24 | 0.89 | 30.60 |
| Serbia | 2007 | 141.32 | 1.54 | 389.85 | 485.38 | 74.63 | 66.69 | 0.00 | 0.29 | 1.25 | 0.89 | 30.72 |
| Serbia | 2008 | 142.19 | 1.55 | 392.89 | 489.48 | 75.54 | 66.65 | 0.00 | 0.29 | 1.25 | 0.88 | 30.47 |
| Serbia | 2009 | 141.65 | 1.52 | 395.45 | 493.13 | 76.33 | 65.32 | 0.00 | 0.29 | 1.25 | 0.86 | 29.61 |
| Serbia | 2010 | 138.23 | 1.45 | 396.83 | 495.41 | 76.94 | 61.28 | 0.00 | 0.28 | 1.25 | 0.80 | 27.68 |
| Serbia | 2011 | 134.47 | 1.38 | 396.65 | 496.16 | 76.93 | 57.53 | 0.00 | 0.27 | 1.25 | 0.75 | 26.00 |
| Serbia | 2012 | 132.53 | 1.35 | 395.38 | 495.91 | 76.63 | 55.89 | 0.00 | 0.27 | 1.25 | 0.73 | 25.34 |
| Serbia | 2013 | 130.27 | 1.31 | 393.59 | 495.10 | 76.35 | 53.92 | 0.00 | 0.26 | 1.26 | 0.71 | 24.49 |
| Serbia | 2014 | 127.84 | 1.28 | 391.87 | 494.09 | 75.65 | 52.18 | 0.00 | 0.26 | 1.26 | 0.69 | 23.81 |
| Serbia | 2015 | 126.03 | 1.27 | 390.74 | 493.03 | 75.02 | 51.01 | 0.00 | 0.26 | 1.26 | 0.68 | 23.39 |
| Serbia | 2016 | 123.79 | 1.23 | 391.37 | 491.83 | 74.56 | 49.22 | 0.00 | 0.25 | 1.26 | 0.66 | 22.58 |
| Serbia | 2017 | 122.72 | 1.22 | 393.65 | 490.50 | 73.66 | 49.06 | 0.00 | 0.25 | 1.25 | 0.67 | 22.51 |
| Serbia | 2018 | 122.88 | 1.22 | 396.27 | 489.38 | 73.08 | 49.80 | 0.00 | 0.25 | 1.23 | 0.68 | 22.76 |
| Serbia | 2019 | 124.20 | 1.24 | 397.93 | 488.95 | 72.82 | 51.38 | 0.00 | 0.25 | 1.23 | 0.71 | 23.44 |
| Serbia | 2020 | 121.02 | 1.20 | 399.95 | 487.68 | 72.80 | 48.22 | 0.00 | 0.25 | 1.22 | 0.66 | 22.10 |
| Serbia | 2021 | 118.97 | 1.17 | 398.48 | 469.74 | 71.92 | 47.05 | 0.00 | 0.25 | 1.18 | 0.65 | 22.20 |
| Slovakia | 1990 | 131.15 | 0.98 | 392.00 | 731.44 | 89.46 | 41.68 | 0.00 | 0.18 | 1.87 | 0.47 | 13.10 |
| Slovakia | 1991 | 131.14 | 0.97 | 388.81 | 735.71 | 89.85 | 41.29 | 0.00 | 0.18 | 1.89 | 0.46 | 12.89 |
| Slovakia | 1992 | 130.60 | 0.94 | 385.70 | 739.71 | 90.55 | 40.05 | 0.00 | 0.18 | 1.92 | 0.44 | 12.38 |
| Slovakia | 1993 | 130.33 | 0.92 | 382.71 | 743.21 | 91.06 | 39.27 | 0.00 | 0.18 | 1.94 | 0.43 | 12.08 |
| Slovakia | 1994 | 129.97 | 0.90 | 379.87 | 745.98 | 91.63 | 38.34 | 0.00 | 0.17 | 1.96 | 0.42 | 11.70 |
| Slovakia | 1995 | 130.85 | 0.90 | 377.22 | 747.86 | 92.24 | 38.61 | 0.00 | 0.17 | 1.98 | 0.42 | 11.80 |
| Slovakia | 1996 | 131.45 | 0.89 | 374.58 | 750.24 | 93.28 | 38.17 | 0.00 | 0.18 | 2.00 | 0.41 | 11.62 |
| Slovakia | 1997 | 135.00 | 0.92 | 371.96 | 753.98 | 95.45 | 39.55 | 0.00 | 0.18 | 2.03 | 0.41 | 12.19 |
| Slovakia | 1998 | 138.09 | 0.94 | 369.63 | 758.05 | 97.70 | 40.39 | 0.00 | 0.18 | 2.05 | 0.41 | 12.53 |
| Slovakia | 1999 | 141.42 | 0.98 | 367.86 | 761.41 | 99.65 | 41.77 | 0.00 | 0.19 | 2.07 | 0.42 | 13.09 |
| Slovakia | 2000 | 142.37 | 0.97 | 366.92 | 763.07 | 100.67 | 41.71 | 0.00 | 0.19 | 2.08 | 0.41 | 13.06 |
| Slovakia | 2001 | 142.20 | 0.98 | 366.49 | 761.22 | 100.34 | 41.86 | 0.00 | 0.19 | 2.08 | 0.42 | 13.19 |
| Slovakia | 2002 | 140.57 | 0.98 | 366.17 | 756.12 | 98.92 | 41.65 | 0.00 | 0.19 | 2.06 | 0.42 | 13.18 |
| Slovakia | 2003 | 139.50 | 0.99 | 366.11 | 749.89 | 97.72 | 41.78 | 0.00 | 0.19 | 2.05 | 0.43 | 13.34 |
| Slovakia | 2004 | 137.73 | 0.98 | 366.43 | 744.66 | 96.36 | 41.37 | 0.00 | 0.18 | 2.03 | 0.43 | 13.26 |
| Slovakia | 2005 | 136.88 | 0.99 | 367.28 | 742.54 | 95.71 | 41.17 | 0.00 | 0.18 | 2.02 | 0.43 | 13.25 |
| Slovakia | 2006 | 135.85 | 0.97 | 369.37 | 742.73 | 96.04 | 39.81 | 0.00 | 0.18 | 2.01 | 0.41 | 12.76 |
| Slovakia | 2007 | 136.73 | 0.99 | 372.86 | 742.89 | 96.32 | 40.42 | 0.00 | 0.18 | 1.99 | 0.42 | 12.98 |
| Slovakia | 2008 | 136.64 | 0.98 | 377.05 | 742.92 | 96.39 | 40.25 | 0.00 | 0.18 | 1.97 | 0.42 | 12.85 |
| Slovakia | 2009 | 136.56 | 0.98 | 381.23 | 742.68 | 96.61 | 39.95 | 0.00 | 0.18 | 1.95 | 0.41 | 12.73 |
| Slovakia | 2010 | 135.11 | 0.96 | 384.67 | 742.04 | 96.43 | 38.68 | 0.00 | 0.18 | 1.93 | 0.40 | 12.22 |
| Slovakia | 2011 | 133.65 | 0.96 | 386.72 | 737.57 | 95.03 | 38.62 | 0.00 | 0.18 | 1.91 | 0.41 | 12.24 |
| Slovakia | 2012 | 129.62 | 0.95 | 387.65 | 728.08 | 91.59 | 38.03 | 0.00 | 0.18 | 1.88 | 0.42 | 12.09 |
| Slovakia | 2013 | 124.96 | 0.94 | 388.14 | 716.83 | 87.40 | 37.55 | 0.00 | 0.17 | 1.85 | 0.43 | 11.99 |
| Slovakia | 2014 | 121.42 | 0.93 | 388.87 | 707.08 | 83.92 | 37.51 | 0.00 | 0.17 | 1.82 | 0.45 | 12.04 |
| Slovakia | 2015 | 120.71 | 0.95 | 390.53 | 702.08 | 82.39 | 38.33 | 0.00 | 0.17 | 1.80 | 0.47 | 12.41 |
| Slovakia | 2016 | 119.62 | 0.92 | 392.75 | 699.41 | 82.06 | 37.56 | 0.00 | 0.17 | 1.78 | 0.46 | 12.08 |
| Slovakia | 2017 | 120.08 | 0.93 | 394.70 | 695.58 | 82.00 | 38.08 | 0.00 | 0.17 | 1.76 | 0.46 | 12.33 |
| Slovakia | 2018 | 121.07 | 0.94 | 396.33 | 692.28 | 81.94 | 39.13 | 0.00 | 0.17 | 1.75 | 0.48 | 12.74 |
| Slovakia | 2019 | 120.22 | 0.92 | 397.60 | 691.22 | 81.88 | 38.34 | 0.00 | 0.17 | 1.74 | 0.47 | 12.37 |
| Slovakia | 2020 | 118.22 | 0.88 | 398.92 | 691.51 | 82.20 | 36.02 | 0.00 | 0.17 | 1.73 | 0.44 | 11.46 |
| Slovakia | 2021 | 117.59 | 0.87 | 400.07 | 691.79 | 81.93 | 35.66 | 0.00 | 0.17 | 1.73 | 0.44 | 11.30 |
| Slovenia | 1990 | 196.25 | 3.21 | 529.32 | 843.70 | 74.67 | 121.58 | 0.01 | 0.23 | 1.59 | 1.63 | 42.54 |
| Slovenia | 1991 | 204.80 | 3.51 | 534.06 | 839.68 | 75.21 | 129.59 | 0.01 | 0.24 | 1.57 | 1.72 | 45.90 |
| Slovenia | 1992 | 201.76 | 3.45 | 538.28 | 837.08 | 76.32 | 125.45 | 0.01 | 0.24 | 1.56 | 1.64 | 44.11 |
| Slovenia | 1993 | 216.62 | 3.92 | 541.82 | 835.69 | 77.72 | 138.91 | 0.01 | 0.26 | 1.54 | 1.79 | 49.38 |
| Slovenia | 1994 | 224.66 | 4.19 | 544.57 | 835.13 | 79.78 | 144.89 | 0.01 | 0.27 | 1.53 | 1.82 | 51.50 |
| Slovenia | 1995 | 208.37 | 3.67 | 546.40 | 835.18 | 81.80 | 126.57 | 0.01 | 0.25 | 1.53 | 1.55 | 43.85 |
| Slovenia | 1996 | 211.44 | 3.74 | 547.60 | 837.95 | 85.40 | 126.04 | 0.01 | 0.25 | 1.53 | 1.48 | 43.21 |
| Slovenia | 1997 | 214.88 | 3.60 | 548.49 | 845.01 | 90.94 | 123.93 | 0.01 | 0.25 | 1.54 | 1.36 | 40.97 |
| Slovenia | 1998 | 222.55 | 3.56 | 549.08 | 855.14 | 97.38 | 125.17 | 0.01 | 0.26 | 1.56 | 1.29 | 40.04 |
| Slovenia | 1999 | 232.15 | 3.62 | 549.31 | 866.92 | 104.19 | 127.96 | 0.01 | 0.27 | 1.58 | 1.23 | 39.88 |
| Slovenia | 2000 | 234.64 | 3.44 | 549.20 | 879.17 | 110.18 | 124.46 | 0.01 | 0.27 | 1.60 | 1.13 | 37.57 |
| Slovenia | 2001 | 247.50 | 3.68 | 547.06 | 890.90 | 114.94 | 132.56 | 0.01 | 0.28 | 1.63 | 1.15 | 39.66 |
| Slovenia | 2002 | 251.64 | 3.64 | 542.42 | 901.91 | 119.46 | 132.18 | 0.01 | 0.28 | 1.66 | 1.11 | 39.00 |
| Slovenia | 2003 | 257.29 | 3.69 | 537.37 | 912.65 | 123.90 | 133.39 | 0.01 | 0.28 | 1.70 | 1.08 | 39.04 |
| Slovenia | 2004 | 259.26 | 3.66 | 533.88 | 923.34 | 128.20 | 131.07 | 0.01 | 0.28 | 1.73 | 1.02 | 38.09 |
| Slovenia | 2005 | 261.22 | 3.63 | 534.00 | 934.33 | 132.83 | 128.39 | 0.01 | 0.28 | 1.75 | 0.97 | 37.03 |
| Slovenia | 2006 | 270.13 | 3.72 | 538.43 | 948.83 | 138.33 | 131.80 | 0.01 | 0.28 | 1.76 | 0.95 | 37.40 |
| Slovenia | 2007 | 279.57 | 3.75 | 545.59 | 967.16 | 145.02 | 134.55 | 0.01 | 0.29 | 1.77 | 0.93 | 37.22 |
| Slovenia | 2008 | 282.00 | 3.71 | 554.22 | 985.85 | 152.05 | 129.95 | 0.01 | 0.29 | 1.78 | 0.85 | 35.50 |
| Slovenia | 2009 | 290.01 | 4.02 | 562.99 | 1001.15 | 157.33 | 132.68 | 0.01 | 0.29 | 1.78 | 0.84 | 36.52 |
| Slovenia | 2010 | 294.00 | 4.22 | 570.62 | 1009.53 | 160.06 | 133.94 | 0.01 | 0.29 | 1.77 | 0.84 | 37.06 |
| Slovenia | 2011 | 289.34 | 4.19 | 578.36 | 1011.22 | 160.55 | 128.78 | 0.01 | 0.29 | 1.75 | 0.80 | 35.87 |
| Slovenia | 2012 | 291.13 | 4.28 | 587.61 | 1009.34 | 159.95 | 131.18 | 0.01 | 0.29 | 1.72 | 0.82 | 36.39 |
| Slovenia | 2013 | 277.48 | 3.73 | 597.39 | 1005.29 | 158.41 | 119.07 | 0.01 | 0.28 | 1.68 | 0.75 | 32.23 |
| Slovenia | 2014 | 260.17 | 3.17 | 606.74 | 1000.48 | 156.81 | 103.36 | 0.01 | 0.26 | 1.65 | 0.66 | 27.54 |
| Slovenia | 2015 | 269.50 | 3.52 | 614.66 | 996.24 | 155.88 | 113.62 | 0.01 | 0.27 | 1.62 | 0.73 | 30.42 |
| Slovenia | 2016 | 265.90 | 3.48 | 621.00 | 979.82 | 153.32 | 112.58 | 0.01 | 0.27 | 1.58 | 0.73 | 30.33 |
| Slovenia | 2017 | 263.37 | 3.60 | 626.19 | 947.52 | 148.68 | 114.69 | 0.01 | 0.28 | 1.51 | 0.77 | 31.68 |
| Slovenia | 2018 | 263.65 | 3.79 | 630.18 | 914.64 | 143.02 | 120.63 | 0.01 | 0.29 | 1.45 | 0.84 | 34.09 |
| Slovenia | 2019 | 265.64 | 3.87 | 632.91 | 896.51 | 138.62 | 127.02 | 0.01 | 0.30 | 1.42 | 0.92 | 35.99 |
| Slovenia | 2020 | 249.50 | 3.63 | 635.15 | 892.81 | 135.50 | 114.00 | 0.01 | 0.28 | 1.41 | 0.84 | 32.83 |
| Slovenia | 2021 | 247.88 | 3.59 | 634.11 | 884.72 | 131.87 | 116.00 | 0.01 | 0.28 | 1.40 | 0.88 | 33.33 |
| Spain | 1990 | 294.08 | 2.66 | 500.63 | 1405.29 | 174.27 | 119.82 | 0.01 | 0.21 | 2.81 | 0.69 | 24.42 |
| Spain | 1991 | 323.09 | 2.89 | 505.12 | 1450.06 | 187.30 | 135.79 | 0.01 | 0.22 | 2.87 | 0.72 | 26.81 |
| Spain | 1992 | 343.85 | 3.03 | 509.63 | 1495.02 | 199.02 | 144.83 | 0.01 | 0.23 | 2.93 | 0.73 | 27.77 |
| Spain | 1993 | 355.43 | 3.06 | 513.92 | 1538.34 | 209.89 | 145.54 | 0.01 | 0.23 | 2.99 | 0.69 | 27.33 |
| Spain | 1994 | 367.14 | 3.12 | 517.74 | 1578.11 | 219.26 | 147.89 | 0.01 | 0.23 | 3.05 | 0.67 | 27.30 |
| Spain | 1995 | 377.24 | 3.20 | 520.87 | 1612.45 | 226.43 | 150.81 | 0.01 | 0.23 | 3.10 | 0.67 | 27.50 |
| Spain | 1996 | 384.24 | 3.23 | 524.11 | 1648.94 | 233.31 | 150.93 | 0.01 | 0.23 | 3.15 | 0.65 | 27.15 |
| Spain | 1997 | 385.73 | 3.14 | 528.11 | 1692.53 | 240.86 | 144.87 | 0.01 | 0.23 | 3.20 | 0.60 | 25.62 |
| Spain | 1998 | 385.62 | 3.03 | 532.30 | 1737.27 | 248.04 | 137.58 | 0.01 | 0.22 | 3.26 | 0.55 | 23.91 |
| Spain | 1999 | 383.93 | 2.88 | 536.14 | 1777.26 | 254.34 | 129.59 | 0.01 | 0.22 | 3.31 | 0.51 | 22.12 |
| Spain | 2000 | 378.74 | 2.67 | 539.06 | 1806.64 | 258.86 | 119.88 | 0.00 | 0.21 | 3.35 | 0.46 | 20.06 |
| Spain | 2001 | 375.72 | 2.60 | 541.17 | 1832.32 | 261.67 | 114.05 | 0.00 | 0.21 | 3.39 | 0.44 | 18.98 |
| Spain | 2002 | 374.35 | 2.57 | 542.85 | 1861.07 | 264.19 | 110.16 | 0.00 | 0.20 | 3.43 | 0.42 | 18.26 |
| Spain | 2003 | 375.16 | 2.57 | 543.93 | 1886.85 | 266.20 | 108.95 | 0.00 | 0.20 | 3.47 | 0.41 | 17.97 |
| Spain | 2004 | 371.99 | 2.52 | 544.28 | 1903.62 | 266.34 | 105.65 | 0.00 | 0.20 | 3.50 | 0.40 | 17.32 |
| Spain | 2005 | 366.49 | 2.45 | 543.76 | 1905.47 | 264.33 | 102.15 | 0.00 | 0.19 | 3.50 | 0.39 | 16.67 |
| Spain | 2006 | 350.42 | 2.30 | 542.11 | 1861.06 | 254.41 | 96.01 | 0.00 | 0.19 | 3.43 | 0.38 | 15.71 |
| Spain | 2007 | 328.98 | 2.25 | 539.55 | 1764.86 | 235.09 | 93.90 | 0.00 | 0.19 | 3.27 | 0.40 | 15.79 |
| Spain | 2008 | 304.56 | 2.23 | 536.59 | 1649.33 | 212.53 | 92.03 | 0.00 | 0.18 | 3.07 | 0.43 | 16.22 |
| Spain | 2009 | 279.19 | 2.14 | 533.78 | 1547.20 | 192.43 | 86.76 | 0.00 | 0.18 | 2.90 | 0.45 | 15.95 |
| Spain | 2010 | 262.14 | 2.06 | 531.62 | 1491.15 | 180.79 | 81.35 | 0.00 | 0.18 | 2.80 | 0.45 | 15.32 |
| Spain | 2011 | 253.82 | 1.99 | 529.70 | 1477.63 | 175.84 | 77.98 | 0.00 | 0.17 | 2.79 | 0.44 | 14.69 |
| Spain | 2012 | 247.84 | 1.95 | 527.33 | 1477.64 | 171.94 | 75.90 | 0.00 | 0.17 | 2.80 | 0.44 | 14.26 |
| Spain | 2013 | 241.98 | 1.92 | 524.83 | 1485.72 | 168.65 | 73.33 | 0.00 | 0.16 | 2.83 | 0.43 | 13.72 |
| Spain | 2014 | 237.99 | 1.88 | 522.44 | 1496.41 | 165.98 | 72.01 | 0.00 | 0.16 | 2.86 | 0.43 | 13.31 |
| Spain | 2015 | 237.39 | 1.90 | 520.42 | 1503.95 | 164.48 | 72.91 | 0.00 | 0.16 | 2.89 | 0.44 | 13.50 |
| Spain | 2016 | 238.14 | 1.95 | 518.66 | 1512.20 | 163.56 | 74.58 | 0.00 | 0.16 | 2.92 | 0.46 | 13.87 |
| Spain | 2017 | 240.46 | 2.01 | 516.87 | 1524.86 | 163.25 | 77.21 | 0.00 | 0.16 | 2.95 | 0.47 | 14.45 |
| Spain | 2018 | 241.67 | 2.04 | 515.08 | 1536.79 | 162.74 | 78.93 | 0.00 | 0.16 | 2.98 | 0.49 | 14.73 |
| Spain | 2019 | 241.15 | 2.02 | 513.26 | 1542.53 | 162.25 | 78.90 | 0.00 | 0.16 | 3.01 | 0.49 | 14.66 |
| Spain | 2020 | 231.21 | 1.89 | 503.81 | 1531.16 | 158.84 | 72.37 | 0.00 | 0.15 | 3.04 | 0.46 | 13.39 |
| Spain | 2021 | 235.98 | 1.96 | 504.61 | 1522.05 | 158.34 | 77.64 | 0.00 | 0.16 | 3.02 | 0.49 | 14.49 |
| Sweden | 1990 | 136.80 | 1.48 | 396.78 | 637.57 | 72.82 | 63.98 | 0.00 | 0.21 | 1.61 | 0.88 | 24.65 |
| Sweden | 1991 | 142.32 | 1.59 | 394.61 | 622.21 | 74.64 | 67.68 | 0.00 | 0.23 | 1.58 | 0.91 | 26.67 |
| Sweden | 1992 | 148.05 | 1.67 | 393.57 | 609.83 | 76.55 | 71.50 | 0.00 | 0.24 | 1.55 | 0.93 | 28.55 |
| Sweden | 1993 | 155.61 | 1.78 | 393.67 | 601.74 | 78.76 | 76.84 | 0.00 | 0.26 | 1.53 | 0.98 | 30.89 |
| Sweden | 1994 | 161.01 | 1.83 | 394.92 | 599.33 | 81.47 | 79.54 | 0.00 | 0.27 | 1.52 | 0.98 | 31.84 |
| Sweden | 1995 | 170.54 | 1.96 | 397.35 | 603.93 | 84.38 | 86.16 | 0.00 | 0.28 | 1.52 | 1.02 | 34.13 |
| Sweden | 1996 | 184.90 | 2.19 | 410.87 | 615.42 | 88.13 | 96.77 | 0.01 | 0.30 | 1.50 | 1.10 | 37.42 |
| Sweden | 1997 | 202.69 | 2.48 | 440.98 | 631.51 | 92.65 | 110.04 | 0.01 | 0.32 | 1.43 | 1.19 | 40.85 |
| Sweden | 1998 | 220.23 | 2.74 | 481.01 | 650.15 | 98.00 | 122.23 | 0.01 | 0.34 | 1.35 | 1.25 | 43.18 |
| Sweden | 1999 | 237.72 | 2.97 | 524.30 | 669.33 | 103.38 | 134.34 | 0.01 | 0.36 | 1.28 | 1.30 | 45.06 |
| Sweden | 2000 | 253.88 | 3.18 | 564.21 | 687.06 | 108.97 | 144.91 | 0.01 | 0.37 | 1.22 | 1.33 | 46.48 |
| Sweden | 2001 | 261.35 | 3.23 | 608.50 | 706.20 | 115.16 | 146.19 | 0.01 | 0.37 | 1.16 | 1.27 | 44.89 |
| Sweden | 2002 | 265.74 | 3.26 | 662.20 | 728.98 | 122.80 | 142.94 | 0.00 | 0.36 | 1.10 | 1.16 | 42.06 |
| Sweden | 2003 | 273.34 | 3.27 | 714.52 | 752.24 | 130.87 | 142.47 | 0.00 | 0.36 | 1.05 | 1.09 | 40.07 |
| Sweden | 2004 | 286.54 | 3.39 | 754.64 | 772.71 | 137.90 | 148.64 | 0.00 | 0.37 | 1.02 | 1.08 | 40.24 |
| Sweden | 2005 | 291.54 | 3.41 | 771.74 | 787.21 | 143.12 | 148.41 | 0.00 | 0.37 | 1.02 | 1.04 | 39.42 |
| Sweden | 2006 | 297.35 | 3.49 | 769.21 | 796.35 | 145.99 | 151.36 | 0.00 | 0.37 | 1.04 | 1.04 | 39.88 |
| Sweden | 2007 | 312.98 | 3.74 | 759.19 | 803.85 | 148.36 | 164.61 | 0.00 | 0.39 | 1.06 | 1.11 | 42.93 |
| Sweden | 2008 | 327.47 | 3.90 | 743.98 | 811.24 | 150.63 | 176.84 | 0.01 | 0.40 | 1.09 | 1.17 | 45.59 |
| Sweden | 2009 | 337.24 | 4.03 | 725.85 | 819.83 | 153.20 | 184.05 | 0.01 | 0.41 | 1.13 | 1.20 | 47.21 |
| Sweden | 2010 | 350.68 | 4.24 | 707.10 | 831.11 | 156.90 | 193.79 | 0.01 | 0.42 | 1.18 | 1.24 | 49.39 |
| Sweden | 2011 | 364.96 | 4.34 | 683.59 | 852.18 | 163.64 | 201.32 | 0.01 | 0.43 | 1.25 | 1.23 | 50.50 |
| Sweden | 2012 | 385.14 | 4.46 | 652.29 | 884.79 | 174.35 | 210.79 | 0.01 | 0.44 | 1.36 | 1.21 | 51.68 |
| Sweden | 2013 | 412.76 | 4.70 | 617.07 | 922.33 | 186.67 | 226.09 | 0.01 | 0.45 | 1.49 | 1.21 | 54.19 |
| Sweden | 2014 | 445.38 | 5.10 | 581.85 | 958.16 | 198.68 | 246.70 | 0.01 | 0.46 | 1.65 | 1.24 | 58.27 |
| Sweden | 2015 | 469.23 | 5.37 | 550.52 | 985.75 | 208.49 | 260.74 | 0.01 | 0.48 | 1.79 | 1.25 | 61.26 |
| Sweden | 2016 | 479.95 | 5.36 | 519.56 | 1006.92 | 217.05 | 262.91 | 0.01 | 0.48 | 1.94 | 1.21 | 61.89 |
| Sweden | 2017 | 486.81 | 5.30 | 487.94 | 1026.73 | 225.29 | 261.52 | 0.01 | 0.47 | 2.10 | 1.16 | 62.04 |
| Sweden | 2018 | 482.07 | 5.11 | 463.38 | 1043.13 | 231.57 | 250.50 | 0.01 | 0.46 | 2.25 | 1.08 | 60.34 |
| Sweden | 2019 | 472.79 | 4.82 | 453.57 | 1053.93 | 236.29 | 236.50 | 0.01 | 0.45 | 2.32 | 1.00 | 57.23 |
| Sweden | 2020 | 450.72 | 4.39 | 455.00 | 1058.37 | 236.71 | 214.01 | 0.01 | 0.43 | 2.33 | 0.90 | 52.06 |
| Sweden | 2021 | 440.88 | 4.16 | 456.12 | 1060.89 | 237.80 | 203.08 | 0.01 | 0.42 | 2.33 | 0.85 | 49.49 |
| Switzerland | 1990 | 566.46 | 4.71 | 577.58 | 1839.36 | 308.03 | 258.43 | 0.01 | 0.31 | 3.18 | 0.84 | 39.57 |
| Switzerland | 1991 | 573.14 | 4.81 | 583.57 | 1845.41 | 308.27 | 264.86 | 0.01 | 0.31 | 3.16 | 0.86 | 40.27 |
| Switzerland | 1992 | 568.94 | 4.75 | 589.06 | 1846.70 | 307.78 | 261.17 | 0.01 | 0.31 | 3.13 | 0.85 | 39.54 |
| Switzerland | 1993 | 561.22 | 4.64 | 594.10 | 1843.84 | 306.76 | 254.47 | 0.01 | 0.30 | 3.10 | 0.83 | 38.45 |
| Switzerland | 1994 | 562.07 | 4.70 | 598.69 | 1837.42 | 304.86 | 257.21 | 0.01 | 0.31 | 3.07 | 0.84 | 38.87 |
| Switzerland | 1995 | 549.29 | 4.63 | 602.88 | 1828.12 | 302.50 | 246.79 | 0.01 | 0.30 | 3.03 | 0.82 | 37.69 |
| Switzerland | 1996 | 521.97 | 4.27 | 606.28 | 1810.57 | 298.51 | 223.46 | 0.01 | 0.29 | 2.99 | 0.75 | 34.47 |
| Switzerland | 1997 | 496.20 | 3.94 | 608.77 | 1782.78 | 293.29 | 202.91 | 0.01 | 0.28 | 2.93 | 0.69 | 31.68 |
| Switzerland | 1998 | 476.89 | 3.71 | 610.92 | 1750.94 | 287.04 | 189.85 | 0.01 | 0.27 | 2.87 | 0.66 | 29.96 |
| Switzerland | 1999 | 458.70 | 3.51 | 613.23 | 1721.07 | 280.02 | 178.68 | 0.01 | 0.27 | 2.81 | 0.64 | 28.47 |
| Switzerland | 2000 | 446.03 | 3.41 | 616.22 | 1699.15 | 272.47 | 173.56 | 0.01 | 0.26 | 2.76 | 0.64 | 27.77 |
| Switzerland | 2001 | 430.23 | 3.30 | 619.66 | 1680.32 | 264.39 | 165.84 | 0.01 | 0.26 | 2.71 | 0.63 | 26.73 |
| Switzerland | 2002 | 412.54 | 3.17 | 622.59 | 1656.97 | 254.04 | 158.51 | 0.01 | 0.25 | 2.66 | 0.62 | 25.71 |
| Switzerland | 2003 | 395.99 | 3.08 | 624.46 | 1631.70 | 243.13 | 152.86 | 0.00 | 0.24 | 2.61 | 0.63 | 25.01 |
| Switzerland | 2004 | 382.64 | 3.04 | 624.75 | 1607.03 | 232.01 | 150.63 | 0.00 | 0.24 | 2.57 | 0.65 | 24.91 |
| Switzerland | 2005 | 373.65 | 3.06 | 622.91 | 1585.41 | 222.64 | 151.01 | 0.00 | 0.24 | 2.55 | 0.68 | 25.28 |
| Switzerland | 2006 | 362.56 | 3.03 | 616.73 | 1564.06 | 213.52 | 149.04 | 0.00 | 0.23 | 2.54 | 0.70 | 25.32 |
| Switzerland | 2007 | 350.28 | 2.98 | 605.99 | 1540.19 | 204.21 | 146.08 | 0.00 | 0.23 | 2.54 | 0.72 | 25.32 |
| Switzerland | 2008 | 339.00 | 2.97 | 593.10 | 1516.91 | 194.79 | 144.21 | 0.00 | 0.22 | 2.56 | 0.74 | 25.60 |
| Switzerland | 2009 | 326.00 | 2.90 | 580.47 | 1497.16 | 186.66 | 139.34 | 0.00 | 0.22 | 2.58 | 0.75 | 25.30 |
| Switzerland | 2010 | 310.07 | 2.75 | 570.50 | 1483.89 | 181.49 | 128.58 | 0.00 | 0.21 | 2.60 | 0.71 | 23.70 |
| Switzerland | 2011 | 301.41 | 2.67 | 562.40 | 1475.48 | 177.70 | 123.72 | 0.00 | 0.20 | 2.62 | 0.70 | 23.04 |
| Switzerland | 2012 | 292.48 | 2.57 | 554.17 | 1468.47 | 174.19 | 118.29 | 0.00 | 0.20 | 2.65 | 0.68 | 22.16 |
| Switzerland | 2013 | 288.19 | 2.54 | 546.39 | 1463.26 | 171.87 | 116.32 | 0.00 | 0.20 | 2.68 | 0.68 | 22.02 |
| Switzerland | 2014 | 282.60 | 2.48 | 539.66 | 1460.30 | 170.14 | 112.46 | 0.00 | 0.19 | 2.71 | 0.66 | 21.40 |
| Switzerland | 2015 | 280.99 | 2.47 | 534.61 | 1460.07 | 169.38 | 111.60 | 0.00 | 0.19 | 2.73 | 0.66 | 21.33 |
| Switzerland | 2016 | 281.89 | 2.49 | 528.19 | 1443.14 | 169.37 | 112.52 | 0.00 | 0.20 | 2.73 | 0.66 | 21.84 |
| Switzerland | 2017 | 284.41 | 2.58 | 519.38 | 1404.49 | 168.89 | 115.52 | 0.00 | 0.20 | 2.70 | 0.68 | 23.15 |
| Switzerland | 2018 | 285.72 | 2.60 | 511.87 | 1365.84 | 168.80 | 116.93 | 0.01 | 0.21 | 2.67 | 0.69 | 24.00 |
| Switzerland | 2019 | 280.76 | 2.49 | 509.32 | 1349.03 | 168.51 | 112.25 | 0.00 | 0.21 | 2.65 | 0.67 | 23.14 |
| Switzerland | 2020 | 272.27 | 2.31 | 508.66 | 1351.18 | 168.51 | 103.76 | 0.00 | 0.20 | 2.66 | 0.62 | 21.15 |
| Switzerland | 2021 | 277.89 | 2.39 | 506.41 | 1351.04 | 168.70 | 109.19 | 0.00 | 0.21 | 2.67 | 0.65 | 22.32 |
| Ukraine | 1990 | 256.96 | 2.57 | 343.75 | 715.97 | 143.68 | 113.27 | 0.01 | 0.36 | 2.08 | 0.79 | 41.05 |
| Ukraine | 1991 | 273.46 | 2.87 | 344.78 | 724.29 | 145.91 | 127.55 | 0.01 | 0.38 | 2.10 | 0.87 | 45.52 |
| Ukraine | 1992 | 290.76 | 3.20 | 345.37 | 731.99 | 148.08 | 142.68 | 0.01 | 0.40 | 2.12 | 0.96 | 50.25 |
| Ukraine | 1993 | 303.15 | 3.43 | 345.51 | 738.98 | 150.14 | 153.00 | 0.01 | 0.41 | 2.14 | 1.02 | 53.41 |
| Ukraine | 1994 | 316.26 | 3.65 | 345.22 | 745.19 | 152.69 | 163.57 | 0.01 | 0.42 | 2.16 | 1.07 | 56.58 |
| Ukraine | 1995 | 333.90 | 3.99 | 344.52 | 750.49 | 155.03 | 178.87 | 0.01 | 0.44 | 2.18 | 1.15 | 61.45 |
| Ukraine | 1996 | 330.34 | 3.86 | 342.85 | 755.45 | 157.77 | 172.56 | 0.01 | 0.44 | 2.20 | 1.09 | 59.32 |
| Ukraine | 1997 | 320.71 | 3.59 | 340.19 | 760.47 | 161.51 | 159.21 | 0.01 | 0.42 | 2.24 | 0.99 | 55.02 |
| Ukraine | 1998 | 310.16 | 3.27 | 337.31 | 765.10 | 165.74 | 144.42 | 0.01 | 0.41 | 2.27 | 0.87 | 50.26 |
| Ukraine | 1999 | 315.23 | 3.31 | 334.96 | 768.96 | 169.01 | 146.22 | 0.01 | 0.41 | 2.30 | 0.87 | 50.89 |
| Ukraine | 2000 | 322.57 | 3.43 | 333.92 | 771.57 | 170.87 | 151.70 | 0.01 | 0.42 | 2.31 | 0.89 | 52.64 |
| Ukraine | 2001 | 322.38 | 3.42 | 333.69 | 770.52 | 170.60 | 151.78 | 0.01 | 0.42 | 2.31 | 0.89 | 52.64 |
| Ukraine | 2002 | 324.79 | 3.50 | 333.40 | 765.16 | 169.01 | 155.78 | 0.01 | 0.42 | 2.29 | 0.92 | 54.08 |
| Ukraine | 2003 | 323.40 | 3.51 | 333.24 | 757.72 | 165.95 | 157.46 | 0.01 | 0.43 | 2.27 | 0.95 | 54.78 |
| Ukraine | 2004 | 333.75 | 3.73 | 333.41 | 750.52 | 163.50 | 170.24 | 0.01 | 0.44 | 2.25 | 1.04 | 58.83 |
| Ukraine | 2005 | 347.82 | 3.96 | 334.10 | 745.81 | 161.77 | 186.05 | 0.01 | 0.47 | 2.23 | 1.15 | 63.47 |
| Ukraine | 2006 | 345.38 | 3.84 | 336.83 | 745.99 | 161.37 | 184.01 | 0.01 | 0.46 | 2.21 | 1.14 | 62.08 |
| Ukraine | 2007 | 358.09 | 4.01 | 342.03 | 750.58 | 162.77 | 195.32 | 0.01 | 0.48 | 2.19 | 1.20 | 64.67 |
| Ukraine | 2008 | 355.60 | 3.90 | 348.19 | 757.61 | 164.70 | 190.90 | 0.01 | 0.47 | 2.18 | 1.16 | 62.41 |
| Ukraine | 2009 | 318.73 | 3.11 | 353.80 | 765.22 | 167.23 | 151.50 | 0.01 | 0.42 | 2.16 | 0.91 | 49.69 |
| Ukraine | 2010 | 301.18 | 2.73 | 357.35 | 771.45 | 169.23 | 131.95 | 0.01 | 0.39 | 2.16 | 0.78 | 43.44 |
| Ukraine | 2011 | 292.02 | 2.52 | 359.12 | 776.03 | 170.52 | 121.50 | 0.01 | 0.38 | 2.16 | 0.71 | 40.09 |
| Ukraine | 2012 | 290.77 | 2.46 | 360.48 | 780.35 | 172.04 | 118.73 | 0.01 | 0.37 | 2.16 | 0.69 | 39.09 |
| Ukraine | 2013 | 288.17 | 2.36 | 361.60 | 784.94 | 173.53 | 114.64 | 0.01 | 0.37 | 2.17 | 0.66 | 37.58 |
| Ukraine | 2014 | 287.88 | 2.30 | 362.65 | 790.33 | 175.71 | 112.17 | 0.01 | 0.36 | 2.18 | 0.64 | 36.63 |
| Ukraine | 2015 | 276.49 | 2.01 | 363.80 | 797.08 | 178.60 | 97.90 | 0.01 | 0.35 | 2.19 | 0.55 | 32.30 |
| Ukraine | 2016 | 274.79 | 1.92 | 364.76 | 804.68 | 181.80 | 92.99 | 0.01 | 0.34 | 2.21 | 0.51 | 30.77 |
| Ukraine | 2017 | 280.25 | 1.96 | 365.34 | 812.03 | 185.73 | 94.52 | 0.01 | 0.35 | 2.22 | 0.51 | 31.20 |
| Ukraine | 2018 | 289.31 | 2.06 | 365.86 | 819.10 | 189.39 | 99.92 | 0.01 | 0.35 | 2.24 | 0.53 | 32.61 |
| Ukraine | 2019 | 294.97 | 2.10 | 366.68 | 825.77 | 192.64 | 102.33 | 0.01 | 0.36 | 2.25 | 0.53 | 33.18 |
| Ukraine | 2020 | 295.00 | 2.04 | 369.14 | 832.26 | 195.04 | 99.95 | 0.01 | 0.35 | 2.25 | 0.51 | 32.24 |
| Ukraine | 2021 | 296.55 | 2.07 | 369.61 | 833.14 | 194.64 | 101.90 | 0.01 | 0.36 | 2.25 | 0.52 | 32.69 |
| United Kingdom | 1990 | 267.21 | 2.15 | 480.36 | 1599.88 | 173.20 | 94.02 | 0.00 | 0.17 | 3.33 | 0.54 | 17.35 |
| United Kingdom | 1991 | 288.13 | 2.29 | 483.05 | 1648.42 | 187.28 | 100.86 | 0.00 | 0.17 | 3.41 | 0.54 | 18.34 |
| United Kingdom | 1992 | 308.13 | 2.41 | 485.77 | 1692.85 | 200.38 | 107.75 | 0.00 | 0.18 | 3.48 | 0.54 | 19.32 |
| United Kingdom | 1993 | 332.30 | 2.67 | 488.37 | 1730.98 | 212.03 | 120.27 | 0.01 | 0.19 | 3.54 | 0.57 | 21.50 |
| United Kingdom | 1994 | 354.94 | 2.88 | 490.69 | 1760.63 | 221.56 | 133.38 | 0.01 | 0.20 | 3.59 | 0.60 | 23.59 |
| United Kingdom | 1995 | 377.33 | 3.17 | 492.57 | 1779.65 | 228.41 | 148.92 | 0.01 | 0.21 | 3.61 | 0.65 | 26.28 |
| United Kingdom | 1996 | 397.71 | 3.48 | 494.42 | 1790.14 | 233.31 | 164.41 | 0.01 | 0.22 | 3.62 | 0.70 | 29.09 |
| United Kingdom | 1997 | 412.00 | 3.64 | 496.69 | 1797.37 | 237.62 | 174.38 | 0.01 | 0.23 | 3.62 | 0.73 | 30.66 |
| United Kingdom | 1998 | 426.89 | 3.82 | 499.29 | 1803.93 | 241.73 | 185.16 | 0.01 | 0.24 | 3.61 | 0.77 | 32.35 |
| United Kingdom | 1999 | 442.66 | 4.04 | 502.12 | 1812.36 | 246.00 | 196.66 | 0.01 | 0.24 | 3.61 | 0.80 | 34.25 |
| United Kingdom | 2000 | 450.77 | 4.07 | 505.09 | 1825.15 | 250.79 | 199.98 | 0.01 | 0.25 | 3.61 | 0.80 | 34.43 |
| United Kingdom | 2001 | 461.90 | 4.07 | 508.94 | 1847.08 | 257.68 | 204.22 | 0.01 | 0.25 | 3.63 | 0.79 | 34.40 |
| United Kingdom | 2002 | 469.48 | 4.02 | 513.86 | 1877.72 | 266.61 | 202.87 | 0.01 | 0.25 | 3.65 | 0.76 | 33.55 |
| United Kingdom | 2003 | 474.43 | 3.95 | 519.04 | 1911.96 | 276.31 | 198.13 | 0.01 | 0.25 | 3.68 | 0.72 | 32.27 |
| United Kingdom | 2004 | 481.33 | 3.95 | 523.68 | 1944.75 | 285.09 | 196.23 | 0.01 | 0.25 | 3.71 | 0.69 | 31.62 |
| United Kingdom | 2005 | 490.98 | 4.02 | 526.99 | 1971.01 | 291.49 | 199.50 | 0.01 | 0.25 | 3.74 | 0.68 | 31.86 |
| United Kingdom | 2006 | 507.03 | 4.25 | 528.89 | 1991.86 | 294.76 | 212.27 | 0.01 | 0.25 | 3.77 | 0.72 | 33.65 |
| United Kingdom | 2007 | 520.65 | 4.47 | 530.00 | 2011.07 | 296.07 | 224.57 | 0.01 | 0.26 | 3.79 | 0.76 | 35.41 |
| United Kingdom | 2008 | 529.26 | 4.64 | 530.57 | 2027.98 | 296.21 | 233.05 | 0.01 | 0.26 | 3.82 | 0.79 | 36.65 |
| United Kingdom | 2009 | 526.56 | 4.63 | 530.88 | 2041.86 | 296.18 | 230.39 | 0.01 | 0.26 | 3.85 | 0.78 | 36.25 |
| United Kingdom | 2010 | 520.41 | 4.55 | 531.19 | 2052.03 | 296.96 | 223.45 | 0.01 | 0.25 | 3.86 | 0.75 | 35.19 |
| United Kingdom | 2011 | 514.64 | 4.46 | 531.48 | 2060.69 | 298.71 | 215.93 | 0.01 | 0.25 | 3.88 | 0.72 | 34.12 |
| United Kingdom | 2012 | 516.92 | 4.54 | 531.62 | 2069.56 | 300.84 | 216.09 | 0.01 | 0.25 | 3.89 | 0.72 | 34.35 |
| United Kingdom | 2013 | 533.72 | 4.94 | 531.70 | 2077.40 | 302.94 | 230.77 | 0.01 | 0.26 | 3.91 | 0.76 | 37.13 |
| United Kingdom | 2014 | 550.95 | 5.30 | 531.80 | 2082.91 | 304.79 | 246.16 | 0.01 | 0.26 | 3.92 | 0.81 | 39.82 |
| United Kingdom | 2015 | 572.59 | 5.77 | 531.99 | 2084.77 | 305.96 | 266.63 | 0.01 | 0.27 | 3.92 | 0.87 | 43.39 |
| United Kingdom | 2016 | 594.78 | 6.30 | 531.90 | 2077.49 | 306.31 | 288.48 | 0.01 | 0.29 | 3.91 | 0.94 | 47.44 |
| United Kingdom | 2017 | 609.32 | 6.69 | 531.44 | 2060.96 | 306.07 | 303.25 | 0.01 | 0.30 | 3.88 | 0.99 | 50.43 |
| United Kingdom | 2018 | 630.85 | 7.15 | 531.00 | 2041.98 | 305.37 | 325.48 | 0.01 | 0.31 | 3.85 | 1.07 | 54.39 |
| United Kingdom | 2019 | 628.89 | 7.06 | 530.96 | 2027.19 | 304.52 | 324.37 | 0.01 | 0.31 | 3.82 | 1.07 | 54.11 |
| United Kingdom | 2020 | 575.87 | 6.31 | 521.03 | 1932.59 | 290.56 | 285.31 | 0.01 | 0.30 | 3.71 | 0.98 | 49.47 |
| United Kingdom | 2021 | 588.75 | 6.50 | 518.89 | 1931.36 | 291.65 | 297.10 | 0.01 | 0.30 | 3.72 | 1.02 | 51.38 |

Note: Values are retained to two decimal places.
