# Supplementary material for: Pharmacovigilance-related events, disease burden and overall efficiency of care in european countries, 1990-2021
Source: Front Pharmacol. 2025 Jun 27;16:1592957. doi: 10.3389/fphar.2025.1592957 (PMC12245805; doi:10.3389/fphar.2025.1592957)
Supplement: Supplementary file 4 [file Supplementaryfile2.docx]

**Appendix 2 Table 1.**Original data of Input and output evaluation indicators.

| **QCI** | **Country** | **DOC** | **PHAR** | **NUR** | **GOV** | **MED** | **EDU** | **UMCTIME** |
| --- | --- | --- | --- | --- | --- | --- | --- | --- |
| 27.37 | Albania | 18.80 | 10.90 | 58.30 | 449.13 | 1131.67 | 60.32 | 2020 |
| 35.48 | Austria | 54.30 | 7.61 | 109.30 | 5690.00 | 7272.00 | 93.94 | 1991 |
| 47.27 | Belarus | 44.70 | 4.34 | 105.23 | 1031.00 | 1389.00 | 73.96 | 2006 |
| 44.98 | Belgium | 32.40 | 13.07 | 205.00 | 5058.62 | 6519.20 | 82.69 | 1977 |
| 47.89 | Bosnia and Herzegovina | 23.20 | 1.38 | 64.00 | 1156.00 | 1693.00 | 43.58 | 2019 |
| 15.37 | Bulgaria | 42.90 | 8.86 | 46.59 | 1502.00 | 2391.00 | 74.03 | 1975 |
| 22.25 | Croatia | 36.10 | 8.23 | 76.80 | 2273.00 | 2703.00 | 72.33 | 1992 |
| 34.13 | Cyprus | 35.50 | 13.30 | 46.30 | 3562.00 | 4206.00 | 96.54 | 2000 |
| 13.68 | Czechia | 42.50 | 7.15 | 93.60 | 3661.93 | 4249.36 | 69.11 | 1992 |
| 49.37 | Denmark | 43.80 | 5.56 | 106.40 | 6001.00 | 7043.00 | 83.98 | 1971 |
| 41.54 | Estonia | 34.38 | 7.13 | 68.70 | 2481.98 | 3258.78 | 73.15 | 1998 |
| 58.81 | Finland | 43.80 | 20.30 | 522.00 | 4603.00 | 5613.00 | 100.87 | 1974 |
| 33.81 | France | 33.43 | 9.61 | 93.80 | 4787.01 | 6330.47 | 68.97 | 1986 |
| 34.43 | Germany | 45.20 | 6.72 | 123.00 | 6013.25 | 7606.96 | 75.67 | 1968 |
| 60.73 | Greece | 63.70 | 10.93 | 40.90 | 1693.81 | 2860.61 | 150.20 | 1990 |
| 15.71 | Hungary | 33.00 | 8.14 | 55.10 | 1955.00 | 2706.00 | 56.52 | 1990 |
| 41.59 | Ireland | 40.62 | 11.10 | 137.27 | 5569.74 | 7196.31 | 78.78 | 1968 |
| 48.56 | Israel | 36.54 | 8.43 | 59.10 | 2499.00 | 3665.00 | 58.99 | 1973 |
| 11.99 | Italy | 40.97 | 12.80 | 64.90 | 3300.00 | 4372.00 | 71.29 | 1975 |
| 33.83 | Latvia | 33.77 | 8.81 | 44.20 | 2186.00 | 3153.00 | 93.48 | 2002 |
| 42.43 | Lithuania | 45.00 | 10.25 | 82.40 | 2305.00 | 3432.00 | 71.91 | 2005 |
| 18.50 | Netherlands | 39.10 | 2.18 | 116.60 | 5006.00 | 7179.00 | 88.95 | 1968 |
| 14.14 | North Macedonia | 29.64 | 5.24 | 48.30 | 836.00 | 1535.00 | 40.55 | 2000 |
| 45.45 | Norway | 51.70 | 9.14 | 188.90 | 7108.00 | 8275.00 | 93.92 | 1971 |
| 19.72 | Poland | 33.90 | 7.42 | 63.40 | 1771.00 | 2456.00 | 70.93 | 1972 |
| 31.34 | Portugal | 57.70 | 9.84 | 77.90 | 2582.00 | 4091.00 | 71.87 | 1993 |
| 26.43 | Republic of Moldova | 32.50 | 3.95 | 61.18 | 787.00 | 1207.00 | 61.30 | 2003 |
| 32.66 | Romania | 34.70 | 11.10 | 80.70 | 1770.00 | 2340.00 | 55.27 | 1976 |
| 53.34 | Russian Federation | 38.30 | 0.40 | 62.40 | 1800.00 | 2530.00 | 57.13 | 1998 |
| 22.20 | Serbia | 28.37 | 1.48 | 59.33 | 1347.51 | 2155.16 | 69.29 | 2000 |
| 33.33 | Slovenia | 33.30 | 7.37 | 105.90 | 3049.00 | 4165.00 | 82.24 | 2010 |
| 14.49 | Spain | 44.80 | 12.50 | 65.40 | 3127.38 | 4367.58 | 94.59 | 1984 |
| 49.49 | Sweden | 71.50 | 16.20 | 217.00 | 5829.00 | 6784.00 | 85.94 | 1968 |
| 22.32 | Switzerland | 44.40 | 6.62 | 187.60 | 3258.63 | 8998.39 | 71.89 | 1991 |
| 32.69 | Ukraine | 29.90 | 0.34 | 66.60 | 552.00 | 1082.00 | 75.91 | 2002 |
| 51.38 | United Kingdom | 31.70 | 8.45 | 91.70 | 5155.13 | 6159.80 | 77.01 | 1968 |

Note: Values are retained to two decimal places.

**Appendix 2 Table 2.** Descriptive analysis of relevant inputs in each country

| item | Sample size | average value | standard deviation |
| --- | --- | --- | --- |
| UMCTIME | 36 | 1988.22 | 15.39 |
| DOC | 36 | 39.61 | 10.62 |
| PHAR | 36 | 8.25 | 4.31 |
| NUR | 36 | 102.66 | 85.28 |
| GOV | 36 | 3104.36 | 1851.43 |
| EDU | 36 | 76.31 | 19.09 |
| QCI | 36 | 34.13 | 13.97 |

Note: Values are retained to two decimal places.

The time of inclusion of Uppsala Monitoring Center (UMC) in this study was an inverse indicator, i.e., the earlier the UMC was added, the longer the pharmacovigilance history in the country, so the indicator needed to be reversed.

$$X’=\frac{X_{\max}-X}{X_{\max}-X_{\min}}$$

The correlation coefficient and variance inflation factor were calculated to perform collinearity analysis for all input indicators.

$$r_{\mathrm{xy}}=\frac{Cov（X,Y）}{\sigma_{x}\sigma_{y}}$$

Where, Cov (X,Y): covariance of variables X and Y, $\sigma_{x}$standard deviation of variable X, standard $\sigma_{y}$deviation of variable Y.

The formula for calculating VIF is:

$$\mathrm{VIF}_{i}=\frac{1}{1-R_{i}^{2}}$$

$R_{i}^{2}$: The coefficient of determination obtained when the independent variable is used as the dependent variable for linear regression and all the remaining independent variables are used for prediction$X_{i}$

Combined with the correlation coefficient and VIF value, it is judged whether it has collinearity.

**Appendix 2 Table 3.** Pearson correlation coefficients

|  | **DOC** | **PHAR** | **NUR** | **GOV** | **MED** | **EDU** | **UMCTIME** |
| --- | --- | --- | --- | --- | --- | --- | --- |
| **DOC** | 1 |  |  |  |  |  |  |
| **PHAR** | 0.381* | 1 |  |  |  |  |  |
| **NUR** | 0.281 | 0.529** | 1 |  |  |  |  |
| **GOV** | 0.456** | 0.370* | 0.491** | 1 |  |  |  |
| **MED** | 0.458** | 0.312 | 0.493** | 0.929** | 1 |  |  |
| **EDU** | 0.588** | 0.409* | 0.264 | 0.375* | 0.355* | 1 |  |
| **UMCTIME** | -0.415* | -0.373* | -0.362* | -0.685** | -0.632** | -0.255 | 1 |

**Appendix 2 Table 4.** Colinearity diagnosis

| **Item** | **VIF value** | **Tolerance** |
| --- | --- | --- |
| DOC | 1.800 | 0.556 |
| PHAR | 1.688 | 0.592 |
| NUR | 1.675 | 0.597 |
| GOV | **8.606** | **0.116** |
| MED | **7.799** | **0.128** |
| EDU | 1.674 | 0.598 |
| UMCTIME | 2.010 | 0.498 |

Among them, the GOV and MED VIF values are greater than 5 and the tolerance is less than 0.2, indicating that there is a collinearity problem, and the personal medical expenditure is moved out of the model, and after collinearity analysis is removed, there is no collinearity problem among the remaining indicators.
