# Supplementary material for: Pharmacovigilance-related events, disease burden and overall efficiency of care in european countries, 1990-2021
Source: Front Pharmacol. 2025 Jun 27;16:1592957. doi: 10.3389/fphar.2025.1592957 (PMC12245805; doi:10.3389/fphar.2025.1592957)
Supplement: Supplementary file 5 [file Supplementaryfile4.docx]

**Appendix 4 Table 1.** Invest in redundancy analysis

Effectiveness analysis

- [Line chart](javascript:void(0);)
- [data](javascript:void(0);)



Created with Highcharts 8.2.0 Effectiveness Analysis: 1.000.521.000.701.000.990.710.960.610.620.821.000.670.561.000.880.571.000.741.000.720.601.000.460.810.690.940.761.001.000.650.710.550.591.001.001.000.770.960.971.000.330.500.770.310.960.880.650.730.741.000.370.880.950.280.770.880.390.400.950.450.680.750.711.000.460.750.330.950.481.000.791.000.400.960.671.000.330.360.740.190.600.730.650.490.411.000.330.500.950.210.770.630.240.400.440.360.470.700.541.000.460.480.240.520.281.000.79Technical BenefitsEconomies of ScaleComprehensive BenefitsAlbaniaAustriaBelarusBelgiumBosnia and HerzegovinaBulgariaCroatiaCyprusCzechiaDenmarkEstoniaFinlandFranceGermanyGreeceHungaryIrelandIsraelItalyLatviaLithuaniaNetherlandsNorth MacedoniaNorwayPolandPortugalRepublic of MoldovaRomaniaRussian FederationSerbiaSloveniaSpainSwedenSwitzerlandUkraineUnited Kingdom010.250.50.751.25

[Copy and download the Hide Tab style](javascript:void(0);)

| item | Relaxation variable S-analysis | | | | | | | Invest in redundancy rates | | | | | |
| --- | --- | --- | --- | --- | --- | --- | --- | --- | --- | --- | --- | --- | --- |
|  | NMMS_umctime | doc | phar | nur | gov | edu | Summary | NMMS_umctime | doc | phar | nur | gov | edu |
| Albania | 0.000 | 0.000 | 0.000 | 0.000 | 0.000 | 0.000 | 0.000 | null | 0.000 | 0.000 | 0.000 | 0.000 | 0.000 |
| Austria | 0.080 | 0.000 | 2.061 | 0.000 | 1268.624 | 0.000 | 1270.765 | 0.143 | 0.000 | 0.271 | 0.000 | 0.223 | 0.000 |
| Belarus | 0.118 | 13.033 | 0.000 | 22.648 | 0.000 | 0.000 | 35.799 | 0.437 | 0.292 | 0.000 | 0.215 | 0.000 | 0.000 |
| Belgium | 0.538 | 0.000 | 7.493 | 77.751 | 2316.112 | 14.680 | 2416.574 | 0.651 | 0.000 | 0.573 | 0.379 | 0.458 | 0.178 |
| Bosnia and Herzegovina | 0.000 | 0.000 | 0.000 | 0.000 | 0.000 | 0.000 | 0.000 | 0.000 | 0.000 | 0.000 | 0.000 | 0.000 | 0.000 |
| Bulgaria | 0.152 | 1.166 | 1.829 | 0.000 | 4.917 | 0.000 | 8.064 | 0.176 | 0.027 | 0.206 | 0.000 | 0.003 | 0.000 |
| Croatia | 0.147 | 0.000 | 1.774 | 0.000 | 254.529 | 0.000 | 256.451 | 0.274 | 0.000 | 0.216 | 0.000 | 0.112 | 0.000 |
| Cyprus | 0.114 | 0.000 | 6.247 | 0.000 | 1743.937 | 13.417 | 1763.715 | 0.297 | 0.000 | 0.470 | 0.000 | 0.490 | 0.139 |
| Czechia | 0.068 | 0.531 | 1.034 | 0.000 | 325.504 | 0.000 | 327.138 | 0.127 | 0.012 | 0.145 | 0.000 | 0.089 | 0.000 |
| Denmark | 0.499 | 0.000 | 1.466 | 0.000 | 2361.607 | 0.000 | 2363.572 | 0.530 | 0.000 | 0.264 | 0.000 | 0.394 | 0.000 |
| Estonia | 0.213 | 0.000 | 2.685 | 0.000 | 766.055 | 1.922 | 770.874 | 0.502 | 0.000 | 0.377 | 0.000 | 0.309 | 0.026 |
| Finland | 0.552 | 0.000 | 11.508 | 260.912 | 1574.265 | 12.095 | 1859.332 | 0.624 | 0.000 | 0.567 | 0.500 | 0.342 | 0.120 |
| France | 0.307 | 0.000 | 3.734 | 0.774 | 1529.368 | 3.029 | 1537.212 | 0.469 | 0.000 | 0.389 | 0.008 | 0.319 | 0.044 |
| Germany | 0.400 | 2.036 | 1.790 | 4.916 | 1658.414 | 0.000 | 1667.556 | 0.400 | 0.045 | 0.266 | 0.040 | 0.276 | 0.000 |
| Greece | 0.000 | 0.000 | 0.000 | 0.000 | 0.000 | 0.000 | 0.000 | 0.000 | 0.000 | 0.000 | 0.000 | 0.000 | 0.000 |
| Hungary | 0.095 | 0.000 | 2.171 | 0.000 | 165.912 | 0.000 | 168.178 | 0.164 | 0.000 | 0.267 | 0.000 | 0.085 | 0.000 |
| Ireland | 0.479 | 0.000 | 4.307 | 12.506 | 1758.624 | 1.230 | 1777.146 | 0.479 | 0.000 | 0.388 | 0.091 | 0.316 | 0.016 |
| Israel | 0.511 | 0.000 | 6.905 | 0.000 | 807.949 | 0.000 | 815.365 | 0.565 | 0.000 | 0.819 | 0.000 | 0.323 | 0.000 |
| Italy | 0.103 | 0.000 | 2.222 | 0.000 | 322.254 | 0.000 | 324.580 | 0.119 | 0.000 | 0.174 | 0.000 | 0.098 | 0.000 |
| Latvia | 0.098 | 0.000 | 3.234 | 0.000 | 799.978 | 14.571 | 817.881 | 0.284 | 0.000 | 0.367 | 0.000 | 0.366 | 0.156 |
| Lithuania | 0.000 | 2.048 | 5.292 | 0.000 | 237.654 | 0.000 | 244.995 | 0.000 | 0.046 | 0.516 | 0.000 | 0.103 | 0.000 |
| Netherlands | 0.220 | 0.000 | 0.000 | 2.843 | 720.725 | 3.916 | 727.705 | 0.220 | 0.000 | 0.000 | 0.024 | 0.144 | 0.044 |
| North Macedonia | 0.126 | 3.906 | 1.583 | 0.000 | 0.000 | 0.000 | 5.615 | 0.327 | 0.132 | 0.302 | 0.000 | 0.000 | 0.000 |
| Norway | 0.397 | 0.747 | 2.715 | 22.439 | 2032.750 | 0.000 | 2059.047 | 0.421 | 0.014 | 0.297 | 0.119 | 0.286 | 0.000 |
| Poland | 0.283 | 0.000 | 1.387 | 0.000 | 150.186 | 0.158 | 152.014 | 0.307 | 0.000 | 0.187 | 0.000 | 0.085 | 0.002 |
| Portugal | 0.000 | 4.703 | 4.337 | 0.000 | 164.449 | 0.000 | 173.490 | 0.000 | 0.082 | 0.441 | 0.000 | 0.064 | 0.000 |
| Republic of Moldova | 0.084 | 4.148 | 2.061 | 0.000 | 0.000 | 0.000 | 6.294 | 0.258 | 0.128 | 0.522 | 0.000 | 0.000 | 0.000 |
| Romania | 0.439 | 2.755 | 5.061 | 0.000 | 160.654 | 0.000 | 168.908 | 0.518 | 0.079 | 0.456 | 0.000 | 0.091 | 0.000 |
| Russian Federation | 0.000 | 0.000 | 0.000 | 0.000 | 0.000 | 0.000 | 0.000 | 0.000 | 0.000 | 0.000 | 0.000 | 0.000 | 0.000 |
| Serbia | 0.104 | 0.000 | 0.000 | 0.000 | 16.436 | 8.765 | 25.304 | 0.269 | 0.000 | 0.000 | 0.000 | 0.012 | 0.126 |
| Slovenia | 0.080 | 0.000 | 2.613 | 6.807 | 673.880 | 9.548 | 692.928 | 0.415 | 0.000 | 0.355 | 0.064 | 0.221 | 0.116 |
| Spain | 0.095 | 0.000 | 1.698 | 0.000 | 351.285 | 0.000 | 353.078 | 0.137 | 0.000 | 0.136 | 0.000 | 0.112 | 0.000 |
| Sweden | 0.504 | 13.489 | 7.062 | 47.564 | 1859.550 | 0.000 | 1928.168 | 0.504 | 0.189 | 0.436 | 0.219 | 0.319 | 0.000 |
| Switzerland | 0.149 | 1.730 | 1.227 | 23.168 | 381.773 | 0.000 | 408.046 | 0.266 | 0.039 | 0.185 | 0.123 | 0.117 | 0.000 |
| Ukraine | 0.000 | 0.000 | 0.000 | 0.000 | 0.000 | 0.000 | 0.000 | 0.000 | 0.000 | 0.000 | 0.000 | 0.000 | 0.000 |
| United Kingdom | 0.765 | 0.000 | 5.154 | 3.338 | 2807.350 | 13.716 | 2830.323 | 0.765 | 0.000 | 0.610 | 0.036 | 0.545 | 0.178 |

**Table description**: the technical efficiency reflects the efficiency brought by the technical factors, and the value is equal to 1 to indicate that the element is used reasonably, otherwise the value is less than1. Explain that there is still room for improvement in the technical efficiency of the elements; The value of 1 indicates that the return of scale remains unchanged (optimal), the value less than 1 indicates that the return of scale is increasing (the scale is too small to expand the scale and increase the benefit), and the value greater than 1 indicates that the return of scale is decreasing (the scale is too large to reduce the scale and increase the benefit); The comprehensive benefit reflects the efficiency of the DMU element of the decision-making unit, which is less than or equal to 1 = technical efficiency * scale efficiency;

The meaning of the relaxation variable S- is 'to achieve the target efficiency when the input is reduced', and the meaning of the relaxation variable S+ is to be 'to achieve the target efficiency when the output is increased'; combined with the comprehensive benefit index, S- and S+ have a total of three indicators, the DEA effectiveness can be judged, if the comprehensive benefit = 1 and S- and S+ are both 0, then 'DEA is strong and effective', if the comprehensive benefit is 1 but S- or S+ is greater than 0, then 'DEA is weakly effective', and if the comprehensive benefit is <1, it is 'non-DEA effective'.

**Appendix 4 Table 2.** Output shortfall analysis

| item | S+ analysis of the relaxation variable | | Underoutput rate |
| --- | --- | --- | --- |
|  | QCI | Summary | QCI |
| Albania | 0.000 | 0.000 | 0.000 |
| Austria | 0.000 | 0.000 | 0.000 |
| Belarus | 0.000 | 0.000 | 0.000 |
| Belgium | 0.000 | 0.000 | 0.000 |
| Bosnia and Herzegovina | 0.000 | 0.000 | 0.000 |
| Bulgaria | 0.000 | 0.000 | 0.000 |
| Croatia | 0.000 | 0.000 | 0.000 |
| Cyprus | 0.000 | 0.000 | 0.000 |
| Czechia | 0.000 | 0.000 | 0.000 |
| Denmark | 0.000 | 0.000 | 0.000 |
| Estonia | 0.000 | 0.000 | 0.000 |
| Finland | 0.000 | 0.000 | 0.000 |
| France | 0.000 | 0.000 | 0.000 |
| Germany | 0.000 | 0.000 | 0.000 |
| Greece | 0.000 | 0.000 | 0.000 |
| Hungary | 0.000 | 0.000 | 0.000 |
| Ireland | 0.000 | 0.000 | 0.000 |
| Israel | 0.000 | 0.000 | 0.000 |
| Italy | 0.000 | 0.000 | 0.000 |
| Latvia | 0.000 | 0.000 | 0.000 |
| Lithuania | 0.000 | 0.000 | 0.000 |
| Netherlands | 0.000 | 0.000 | 0.000 |
| North Macedonia | 0.000 | 0.000 | 0.000 |
| Norway | 0.000 | 0.000 | 0.000 |
| Poland | 0.000 | 0.000 | 0.000 |
| Portugal | 0.000 | 0.000 | 0.000 |
| Republic of Moldova | 0.000 | 0.000 | 0.000 |
| Romania | 0.000 | 0.000 | 0.000 |
| Russian Federation | 0.000 | 0.000 | 0.000 |
| Serbia | 0.000 | 0.000 | 0.000 |
| Slovenia | 0.000 | 0.000 | 0.000 |
| Spain | 0.000 | 0.000 | 0.000 |
| Sweden | 0.000 | 0.000 | 0.000 |
| Switzerland | 0.000 | 0.000 | 0.000 |
| Ukraine | 0.000 | 0.000 | 0.000 |
| United Kingdom | 0.000 | 0.000 | 0.000 |

Note: Input redundancy analysis, the relaxation variable S- of specific input factors; The relaxation variable S- means 'how much input is reduced to achieve the target efficiency', and the input redundancy rate refers to the ratio of 'too much input' to what has been invested, and the higher the value, the more 'too much input'.

Output deficiency analysis of the relaxation variable S+ of specific input factors; The relaxation variable S+ means 'how much output is added to achieve the target efficiency'; The product deficit rate is the ratio of 'insufficient output' to what has been produced, and the higher the value, the more 'insufficient output'.
